# Supplementary material for: Integrative analysis of neuroblastoma by single-cell RNA sequencing identifies the NECTIN2-TIGIT axis as a target for immunotherapy
Source: Cancer Cell. 2024 Feb 12;42(2):283–300.e8. doi: 10.1016/j.ccell.2023.12.008 (PMC10864003; doi:10.1016/j.ccell.2023.12.008)
Supplement: Document S1. Figures S1–S9 [file mmc1.pdf]

Cancer Cell, Volume *42*

Supplemental information

Integrative analysis of neuroblastoma by single-cell RNA sequencing identifies the NECTIN2-TIGIT axis as a target for immunotherapy

Judith Wienke, Lindy L. Visser, Waleed M. Kholosy, Kaylee M. Keller, Marta Barisa, Evon Poon, Sophie Munnings-Tomes, Courtney Himsworth, Elizabeth Calton, Ana Rodriguez, Ronald Bernardi, Femke van den Ham, Sander R. van Hooff, Yvette A.H. Matser, Michelle L. Tas, Karin P.S. Langenberg, Philip Lijnzaad, Anne L. Borst, Elisa Zappa, Francisca J. Bergsma, Josephine G.M. Strijker, Bronte M. Verhoeven, Shenglin Mei, Amira Kramdi, Restuadi Restuadi, Alvaro Sanchez-Bernabeu, Annelisa M. Cornel, Frank C.P. Holstege, Juliet C. Gray, Godelieve A.M. Tytgat, Marijn A. Scheijde-Vermeulen, Marc H.W.A. Wijnen, Miranda P. Dierselhuis, Karin Straathof, Sam Behjati, Wei Wu, Albert J.R. Heck, Jan Koster, Stefan Nierkens, Isabelle Janoueix-Lerosey, Ronald R. de Krijger, Ninib Baryawno, Louis Chesler, John Anderson, Hubert N. Caron, Thanasis Margaritis, Max M. van Noesel, and Jan J. Molenaar

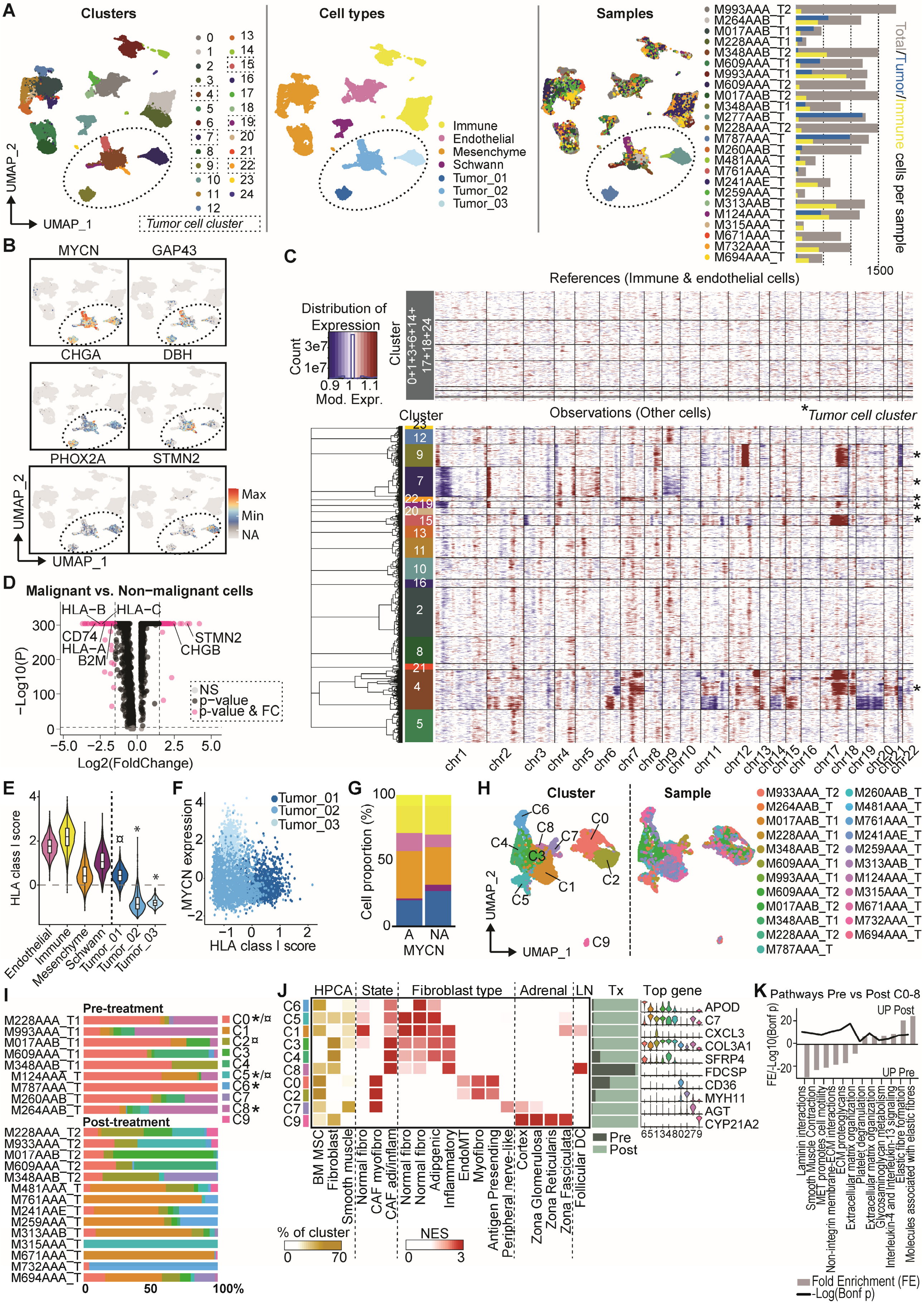

A
Clusters
Cell types
Samples
Immune
Endothelial
Mesenchyme
Schwann
Tumor_01
Tumor_02
Tumor_03
Tumor cell cluster
UMAP_1
UMAP_2
M993AAA_T2
M264AAB_T
M017AAB_T1
M228AAA_T1
M348AAB_T2
M609AAA_T1
M993AAA_T1
M609AAA_T2
M017AAB_T2
M348AAB_T1
M277AAB_T
M228AAA_T2
M787AAA_T
M260AAB_T
M481AAA_T
M761AAA_T
M241AAE_T
M259AAA_T
M313AAB_T
M124AAA_T
M315AAA_T
M671AAA_T
M732AAA_T
M694AAA_T
1500
Total/Tumor/Immune cells per sample
B
MYCN
GAP43
CHGA
DBH
PHOX2A
STMN2
Max
Min
NA
C
References (Immune & endothelial cells)
Distribution of Expression
Count
3e7
1e7
0.9 1 1.1
Mod. Expr.
Cluster
0+1+3+6+14+17+18+24
Observations (Other cells)
*Tumor cell cluster
chr1 chr2 chr3 chr4 chr5 chr6 chr7 chr8 chr9 chr10 chr11 chr12 chr13 chr14 chr15 chr16 chr17 chr18 chr19 chr20 chr21 chr22
D
Malignant vs. Non−malignant cells
HLA−B
HLA−C
CD74
HLA−A
B2M
STMN2
CHGB
NS
p−value
p−value & FC
−Log10(P)
Log2(FoldChange)
E
HLA class I score
Endothelial
Immune
Mesenchyme
Schwann
Tumor_01
Tumor_02
Tumor_03
F
MYCN expression
HLA class I score
G
Cell proportion (%)
A
NA
MYCN
H
Cluster
Sample
C0 C1 C2 C3 C4 C5 C6 C7 C8 C9
M933AAA_T2
M264AAB_T
M017AAB_T1
M228AAA_T1
M348AAB_T2
M609AAA_T1
M993AAA_T1
M609AAA_T2
M017AAB_T2
M348AAB_T1
M228AAA_T2
M787AAA_T
M260AAB_T
M481AAA_T
M761AAA_T
M241AAE_T
M259AAA_T
M313AAB_T
M124AAA_T
M315AAA_T
M671AAA_T
M732AAA_T
M694AAA_T
I
Pre-treatment
M228AAA_T1
M993AAA_T1
M017AAB_T1
M609AAA_T1
M348AAB_T1
M124AAA_T
M787AAA_T
M260AAB_T
M264AAB_T
Post-treatment
M228AAA_T2
M933AAA_T2
M017AAB_T2
M609AAA_T2
M348AAB_T2
M481AAA_T
M761AAA_T
M241AAE_T
M259AAA_T
M313AAB_T
M315AAA_T
M671AAA_T
M732AAA_T
M694AAA_T
0 50 100%
C0 */¤
C1
C2 ¤
C3
C4
C5 */¤
C6 *
C7
C8 *
C9
J
HPCA
State
Fibroblast type
Adrenal
LN
Tx
Top gene
APOD
C7
CXCL3
COL3A1
SFRP4
FDCSP
CD36
MYH11
AGT
CYP21A2
6513480279
Pre
Post
BM MSC
Fibroblast
Smooth muscle
Normal fibro
CAF myofibro
CAF adi/inflam
Normal fibro
Normal fibro
Adipogenic
Inflammatory
EndoMT
Myofibro
Antigen Presending
Peripheral nerve-like
Cortex
Zona Glomerulosa
Zona Reticularis
Zona Fasciculata
Follicular DC
% of cluster
0 70
NES
0 3
K
Pathways Pre vs Post C0-8
UP Post
UP Pre
FE/-Log10(Bonf p)
Laminin interactions
Smooth Muscle Contraction
MET promotes cell motility
Non-integrin membrane-ECM interactions
ECM proteoglycans
Extracellular matrix organization
Platelet degranulation
Extracellular matrix organization
Glycosaminoglycan metabolism
Interleukin-4 and Interleukin-13 signaling
Elastic fibre formation
Molecules associated with elastic fibres
Fold Enrichment (FE)
-Log(Bonf p)

Figure S1. The single-cell landscape of neuroblastoma, related to figure 1. (A) UMAP of identified cell clusters, main cell types and sample distribution. Dotted circles highlight the tumor cell clusters. Right panel: number of total cells, tumor cells and immune cells per sample. **(B)** UMAP with expression of typical neuroblastoma genes (extension of Figure 1D). **(C)** Copy number profile of the samples, generated using *inferCNV*, to confirm tumor cell identity. The identified cell clusters (Figure S1A) were used as cell groups, in which immune and endothelial cell clusters were used as reference. *Clusters annotated as tumor cells based on gene expression profile. **(D)** Volcanoplot of differentially expressed genes (padj<0.05) between tumor cells (malignant) and all other cells (non-malignant). **(E)** Violin plot showing the modulescore of HLA class I genes *HLA-A*, *HLA-B*, *HLA-C*, *HLA-E*, *HLA-F* and *B2M* in the main cell types. *Kruskall-Wallis + Dunn's. °p<0.0001 versus all non-malignant clusters except mesenchyme, which was not signficant. *p<0.0001 versus all non-malignant clusters.* **(F)** Correlation between *MYCN* expression and HLA class I gene score for the three tumor clusters. **(G)** Cell proportion in MYCN-A versus MYCN-NA samples. **(H)** UMAP of mesenchyme clusters and sample distribution**. (I)** Proportion of different mesenchymal clusters per sample. "T1" and "T2" refer to paired samples before and after treatment, respectively. *Proportion significantly differs pre vs post treatment over all samples (p<0.05; Mann-Whitney U test). ¤Proportion significantly differs pre- vs post-treatment in paired samples (p<0.05; Wilcoxon matched-ranks test). **(J)** Annotation of mesenchyme clusters with signatures from human protein/cell atlas (HPCA), GSEA using signatures of CAF states and fibroblast types[1], signatures of adrenal cell types[2], and signatures of follicular dendritic cells (FDC)[3]. Distribution pre- vs post-treatment (Tx) per cluster and top differentially expressed gene per cluster. *NES = normalized enrichment score.* **(K)** Pathway analysis (Reactome; Bonferroni p-value<0.05) of combined clusters C0-8 of genes which were significantly upregulated (p<0.05) pre-treatment vs post-treatment using all samples.

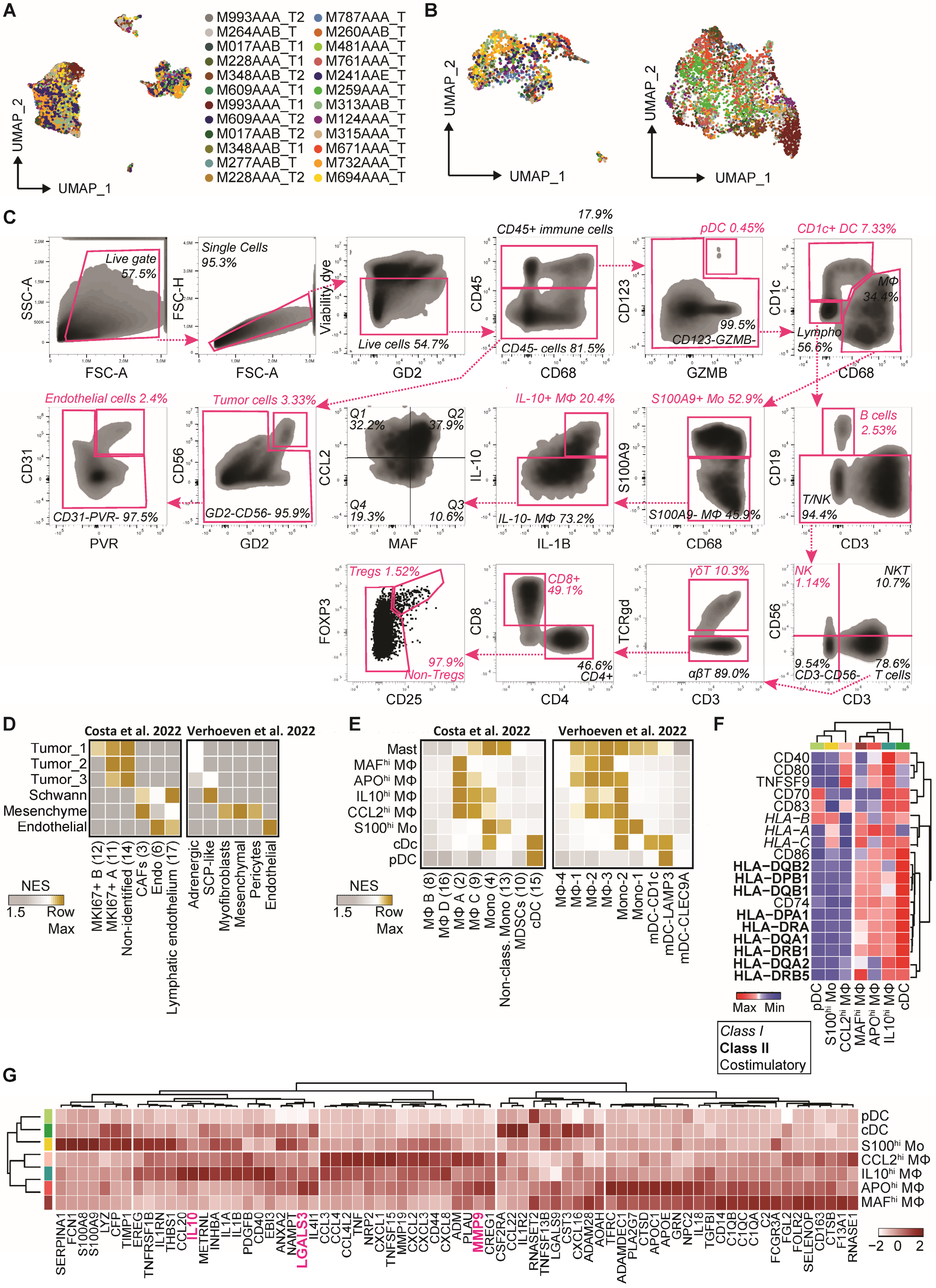

A
B
C
D
E
F
G
UMAP_1
UMAP_2
Live gate 57.5%
Single Cells 95.3%
Live cells 54.7%
17.9% CD45+ immune cells
CD45- cells 81.5%
pDC 0.45%
99.5% CD123-GZMB-
CD1c+ DC 7.33%
MΦ 34.4%
Lympho 56.6%
Endothelial cells 2.4%
CD31-PVR- 97.5%
Tumor cells 3.33%
GD2-CD56- 95.9%
IL-10+ MΦ 20.4%
IL-10- MΦ 73.2%
S100A9+ Mo 52.9%
S100A9- MΦ 45.9%
B cells 2.53%
T/NK 94.4%
Tregs 1.52%
97.9% Non-Tregs
CD8+ 49.1%
46.6% CD4+
γδT 10.3%
αβT 89.0%
NK 1.14%
NKT 10.7%
9.54% CD3-CD56-
78.6% T cells
Costa et al. 2022
Verhoeven et al. 2022
NES
1.5
Row Max
Class I
Class II
Costimulatory
Max Min

Figure S2. The immune environment of neuroblastoma, related to figure 2. (A) UMAP of sample distribution in immune compartment. **(B)** UMAP of sample distribution in myeloid cell (*left*) and lymphoid cell (*right*) compartments. **(C)** Flow cytometry gating strategy to identify cell populations in the tumor microenvironment. Merged data from 5 individual tumor samples are shown. **(D-E)** Gene set enrichment analysis comparing the identified non-immune **(D)** and myeloid **(E)** populations in our dataset to identified populations in the datasets of Costa et al. and Verhoeven et al.[4,5]**.** Gene signatures were derived from FindAllMarkers analysis in Seurat (genes with padj<0.05). NES=normalized enrichment score. **(F)** Heatmap of genes representing antigen presenting/co-stimulatory capacity. Genes are included in module score shown in Figure 2C. **(G)** Heatmap with secreted factors which were 1) specifically upregulated in myeloid cells compared to all other cells (padj<0.05) and 2) among the differentially expressed genes between the myeloid clusters (padj<0.05).

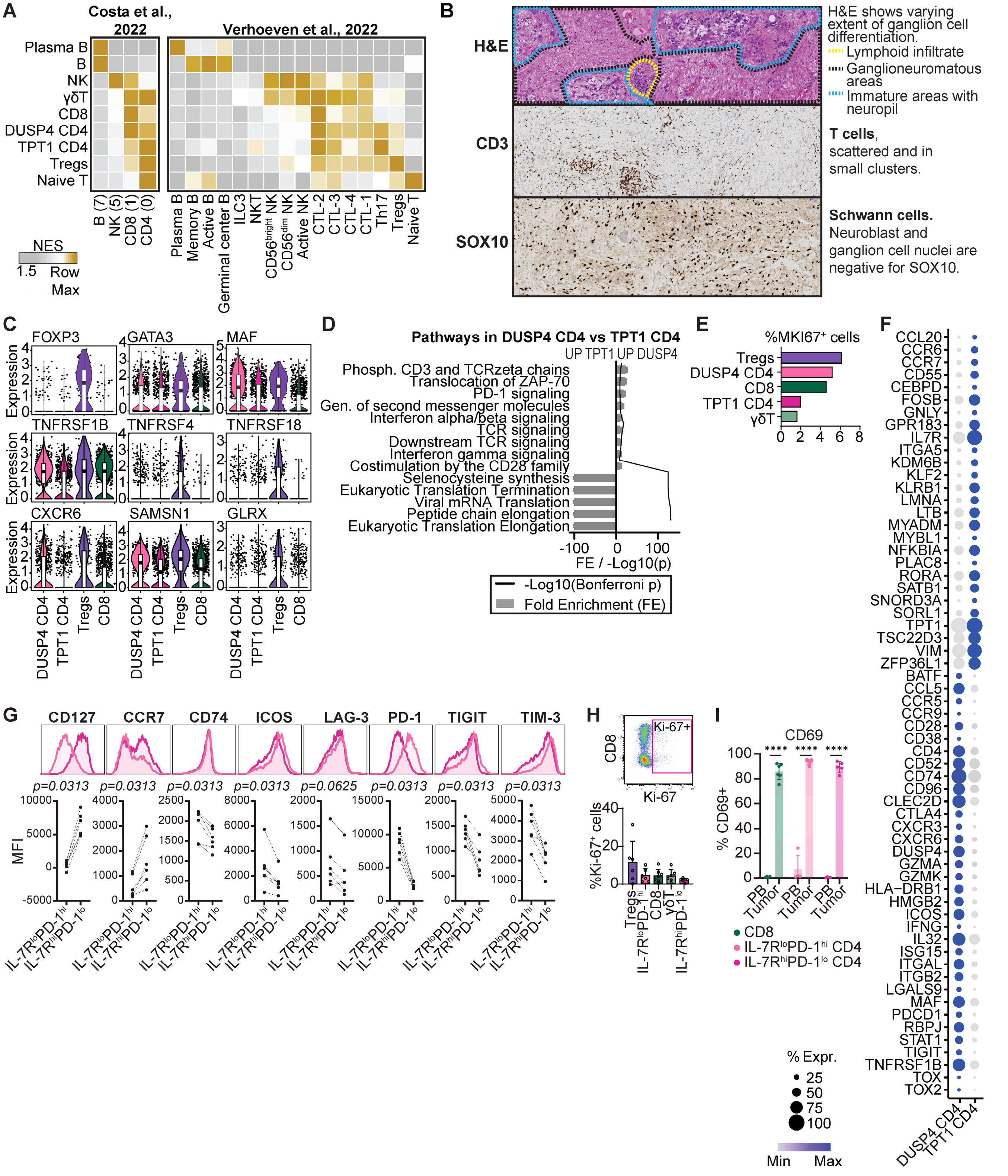

Figure S3. Neuroblastoma is characterized by immunosuppressive and dysfunctional lymphoid populations, related to figure 3. (A) Gene set enrichment analysis comparing the identified lymphoid populations in our dataset to identified lymphoid populations in the datasets of Costa et al. and Verhoeven et al.[4,5]**.** Gene signatures were derived from FindAllMarkers analysis in Seurat (genes with padj<0.05). NES=normalized enrichment score. **(B)** Immunohistochemical staining of hematoxylin & eosin (H&E), CD3 and SOX10 showing T cell infiltration in a representative neuroblastoma lesion. **(C)** Expression of effector Treg marker genes which are

significantly upregulated (*padj<0.05 in FindAllMarkers analysis among lymphocytes*) in neuroblastoma-infiltrating Tregs. **(D)** Pathway analysis (Reactome; Bonferroni-corrected p-value<0.05) of differentially expressed genes (padj<0.05) between the *DUSP4*^{hi} and *TPT1*^{hi} CD4^{+} T cell populations. **(E)** Percentage of T cells expressing proliferation marker *MKI67*. **(F)** Dotplot with selected differentially expressed genes (*padj<0.05 in FindMarkers analysis*) between the two CD4^{+} T effector populations. **(G)** Flow cytometric analysis of the two neuroblastoma-infiltrating CD4^{+} T cell subsets showing memory, activation and exhaustion markers. *Mann-Whitney U test*; *MFI = median fluorescent intensity.* **(H)** Flow cytometric analysis of proliferation marker Ki-67 in neuroblastoma-infiltrating T cell subsets. **(I)** Flow cytometric analysis of tissue-resident T cell marker CD69 in neuroblastoma-infiltrating T cell subsets compared to their counterparts in healthy donor peripheral blood. TPT1^{hi} CD4 were gated as IL-7R^{hi}PD-1^{lo} CD4^{+} cells and DUSP4^{hi} CD4 were gated as IL-7R^{lo}PD-1^{hi} CD4^{+} cells, as shown in figure 3F. 2-way ANOVA. ****P<0.0001.

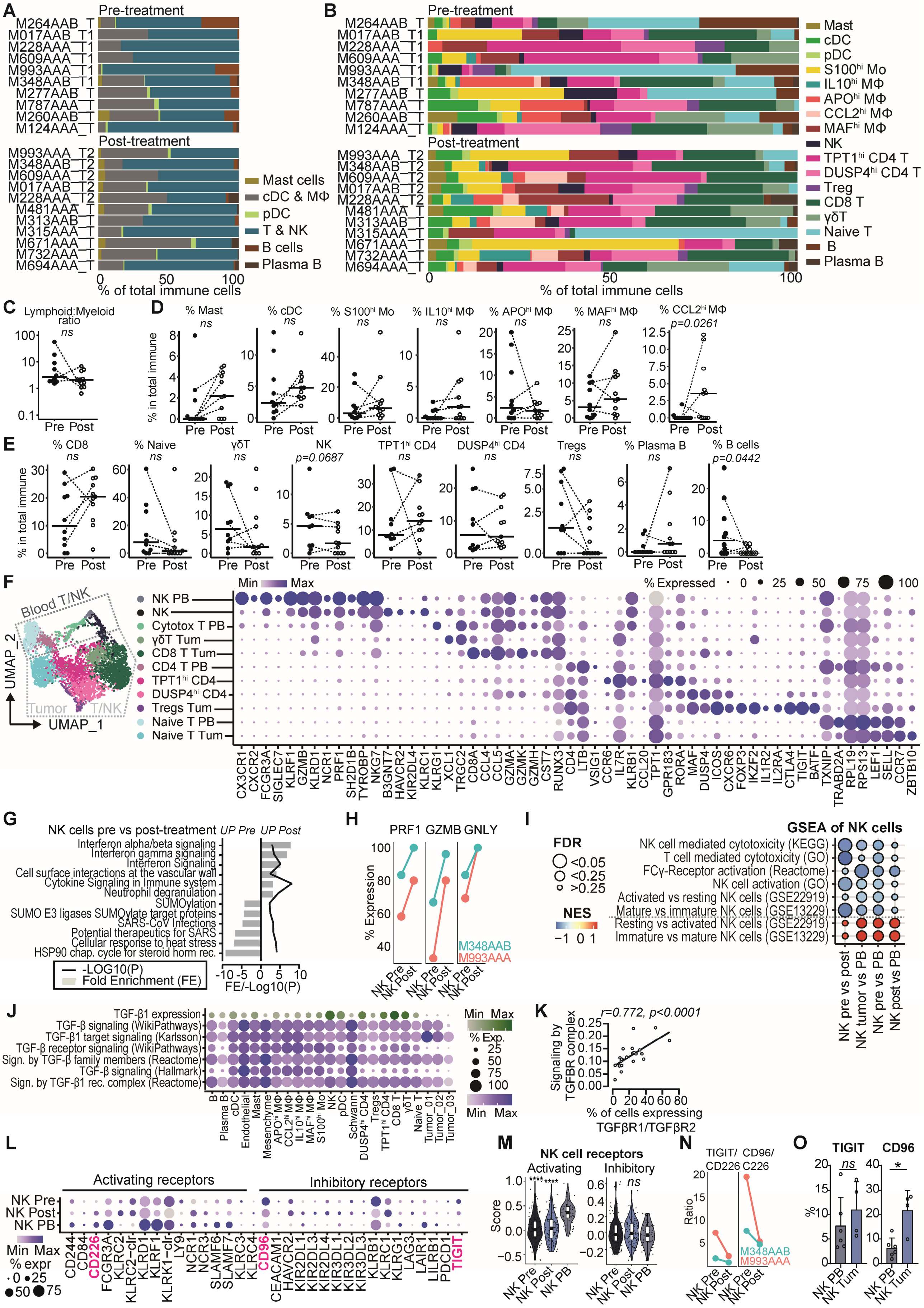

A
Pre-treatment
M264AAB_T
M017AAB_T1
M228AAA_T1
M609AAA_T1
M993AAA_T1
M348AAB_T1
M277AAB_T
M787AAA_T
M260AAB_T
M124AAA_T
Post-treatment
M993AAA_T2
M348AAB_T2
M609AAA_T2
M017AAB_T2
M228AAA_T2
M481AAA_T
M313AAB_T
M315AAA_T
M671AAA_T
M732AAA_T
M694AAA_T
0 50 100
% of total immune cells
Mast cells
cDC & MΦ
pDC
T & NK
B cells
Plasma B
B
Pre-treatment
Post-treatment
0 50 100
% of total immune cells
Mast
cDC
pDC
S100hi Mo
IL10hi MΦ
APOhi MΦ
CCL2hi MΦ
MAFhi MΦ
NK
TPT1hi CD4 T
DUSP4hi CD4 T
Treg
CD8 T
γδT
Naive T
B
Plasma B
C
Lymphoid:Myeloid ratio
ns
Pre Post
D
% in total immune
% Mast ns
% cDC ns
% S100hi Mo ns
% IL10hi MΦ ns
% APOhi MΦ ns
% MAFhi MΦ ns
% CCL2hi MΦ p=0.0261
Pre Post
E
% in total immune
% CD8 ns
% Naive ns
γδT ns
NK p=0.0687
TPT1hi CD4 ns
DUSP4hi CD4 ns
Tregs ns
% Plasma B ns
% B cells p=0.0442
Pre Post
F
Blood T/NK
Tumor T/NK
UMAP_2
UMAP_1
Min Max
% Expressed 0 25 50 75 100
NK PB
NK
Cytotox T PB
γδT Tum
CD8 T Tum
CD4 T PB
TPT1hi CD4
DUSP4hi CD4
Tregs Tum
Naive T PB
Naive T Tum
CX3CR1 CXCR2 FCGR3A SIGLEC7 KLRF1 GZMB KLRD1 NCR1 PRF1 SH2D1B TYROBP NKG7 B3GNT7 HAVCR2 KIR2DL4 KLRC1 KLRG1 XCL1 TRGC2 CD8A CCL4 CCL5 GZMA GZMK GZMH CST7 RUNX3 CD4 LTB VSIG1 CCR6 IL7R KLRB1 CCL20 TPT1 GPR183 RORA MAF DUSP4 ICOS CXCR6 FOXP3 IKZF2 IL1R2 IL2RA CTLA4 TIGIT BATF TXNIP TRABD2A RPL19 RPS13 LEF1 SELL CCR7 ZBTB10
G
NK cells pre vs post-treatment
UP Pre UP Post
Interferon alpha/beta signaling
Interferon gamma signaling
Interferon Signaling
Cell surface interactions at the vascular wall
Cytokine Signaling in Immune system
Neutrophil degranulation
SUMOylation
SUMO E3 ligases SUMOylate target proteins
SARS-CoV Infections
Potential therapeutics for SARS
Cellular response to heat stress
HSP90 chap. cycle for steroid horm rec.
-LOG10(P)
Fold Enrichment (FE)
-10 -5 0 5 10
FE/-Log10(P)
H
PRF1 GZMB GNLY
% Expression
M348AAB
M993AAA
NK Pre NK Post
I
GSEA of NK cells
FDR
<0.05
<0.25
>0.25
NES
-1 0 1
NK cell mediated cytotoxicity (KEGG)
T cell mediated cytotoxicity (GO)
FCγ-Receptor activation (Reactome)
NK cell activation (GO)
Activated vs resting NK cells (GSE22919)
Mature vs immature NK cells (GSE13229)
Resting vs activated NK cells (GSE22919)
Immature vs mature NK cells (GSE13229)
NK pre vs post
NK tumor vs PB
NK pre vs PB
NK post vs PB
J
TGF-β1 expression
TGF-β signaling (WikiPathways)
TGF-β1 target signaling (Karlsson)
TGF-β receptor signaling (WikiPathways)
Sign. by TGF-β family members (Reactome)
TGF-β signaling (Hallmark)
Sign. by TGF-β1 rec. complex (Reactome)
B Plasma B cDC Endothelial Mast Mesenchyme APOhi MΦ CCL2hi MΦ IL10hi MΦ MAFhi MΦ S100hi Mo NK pDC Schwann DUSP4hi CD4 Tregs TPT1hi CD4 CD8 T γδT Naive T Tumor_01 Tumor_02 Tumor_03
Min Max
% Exp.
25 50 75 100
Min Max
K
r=0.772, p<0.0001
Signaling by TGFBR complex
% of cells expressing TGFβR1/TGFβR2
L
Activating receptors
Inhibitory receptors
NK Pre
NK Post
NK PB
CD244 CD84 CD226 FCGR3A KLRC2 KLRC2-clr KLRD1 KLRF1 KLRK1-clr LY9 NCR1 NCR3 SLAMF6 SLAMF7 KLRC3 KLRC4 CD96 CEACAM1 HAVCR2 KIR2DL1 KIR2DL3 KIR2DL4 KIR3DL1 KIR3DL2 KIR3DL3 KLRB1 KLRC1 KLRG1 LAG3 LAIR1 LILRB1 PDCD1 TIGIT
Min Max
% expr
0 25 50 75
M
NK cell receptors
Activating
Inhibitory
Score
**** ****
ns
NK Pre NK Post NK PB
N
TIGIT/CD226
CD96/C226
Ratio
M348AAB
M993AAA
NK Pre NK Post
O
TIGIT
CD96
%
ns
*
NK PB NK Tum

Figure S4. The immune cell composition and function in neuroblastoma before and after induction chemotherapy, related to figure 4. (A) Composition of overall immune cells per patient before and after induction chemotherapy. “T1” and “T2” indicate paired samples before and after treatment. **(B)** Composition of detailed immune cell clusters per patient before and after induction chemotherapy. “T1” and “T2” indicate paired samples before and after treatment. **(C)** Lymphoid:myeloid ratio before and after chemotherapy. Dotted lines indicate paired samples. *Mann-Whitney U test.* **(D)** Myeloid cell percentages before and after induction chemotherapy. Dotted lines indicate paired samples. *Mann-Whitney U test.* **(E)** Lymphoid cell percentages before and after induction chemotherapy. Dotted lines indicate paired samples. *Mann-Whitney U test.* **(F)** UMAP and dotplot of integrated T/NK subsets from both tumor and reference peripheral blood (PB) of adult healthy donors showing a selection of their differentially expressed and marker genes. **(G)** Pathway analysis of NK cells with differentially expressed genes pre- versus post-treatment (nominal p<0.05; Reactome, Bonferroni-corrected p-values<0.05). **(H)** PRF1, GZMB and GNLY expression in two paired samples before and after treatment. The other 3 paired samples did not have sufficient NK cells for a reliable analysis. **(I)** Gene set enrichment analysis of NK cells in pre-treatment tumors compared to NK cells from post-treatment tumors and blood. *NK tumor = pre- and post-treatment NK cells combined. NES=normalized enrichment score*. **(J)** DotPlot of TGF-β1 expression and *modulescores* of curated signatures for TGF-β downstream signaling in the neuroblastoma TME[6]**. (K)** Pearson correlation between expression of signature for downstream signaling by the TGF-β receptor (TGFBR) complex (Reactome) in each population shown in figure S4J and the percentage of cells expressing a *modulescore* of TGF-β receptor 1/2 in those respective populations. **(L)** Dotplot of NK cell activating and inhibitory receptor expression. **(M)** Modulescore of NK cell activating and inhibitory receptors shown in Figure S4L. *Kruskall-Wallis with Dunn’s, ****P<0.0001 vs NK PB, ns=not significant*. **(N)** The TIGIT/CD226 and CD96/CD226 gene expression ratio in two paired samples before and after treatment. The other 3 paired samples did not have sufficient NK cells for a reliable analysis. **(O)** Flow cytometric analysis of TIGIT and CD96 expression in neuroblastoma-infiltrating NK cells compared to blood NK cells. *Mann-Whitney U test; ns=not significant.*

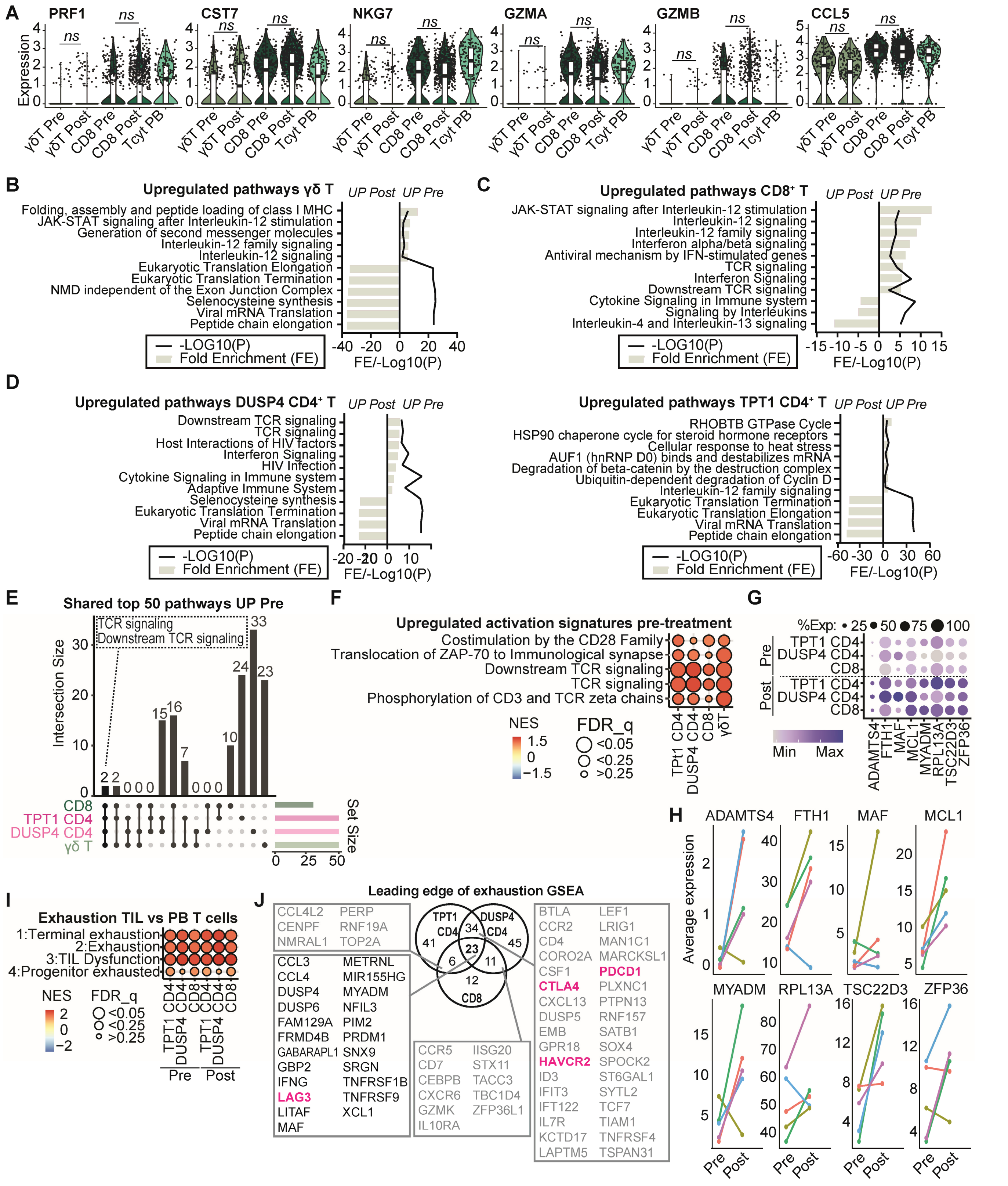

Figure S5. T cells in neuroblastoma show increased dysfunctionality post-treatment, related to figure 4. (A) Violin plots of genes associated with cytotoxicity in γδ T and CD8 T cells pre- and post-treatment compared to cytotoxic T cells in reference blood (Tcyt PB). *ns=not significant.* **(B-D)** Pathway analysis of differentially expressed genes (padj<0.05) in γδ T **(B)**, CD8 T **(C)** and CD4 T **(D)** pre- versus post-treatment (Reactome, Bonferroni-corrected p-values<0.05). **(E)** UpsetR 'Venn' diagram of top 50 upregulated pathways pre-treatment compared to post-treatment in the T cell subsets. **(F)** Gene set enrichment analysis (GSEA) with gene signatures for

TCR signaling in T cells pre-treatment vs post-treatment (all Reactome signatures). **(G)** Dotplot of shared upregulated genes comparing expression in CD4 and CD8 T cells pre- and post-treatment. **(H)** Analysis of gene expression of the eight shared upregulated genes post-treatment in αβ T cells in five paired samples pre-treatment and post-treatment. **(I)** GSEA of exhaustion signatures (terminal & progenitor: GSE84105, exhaustion: [7], dysfunction: [8]) in indicated CD4 and CD8 T cell clusters versus their respective counterparts from reference blood. **(J)** Venn diagram of shared upregulated genes post-treatment versus pre-treatment, which are present in leading edges (core enriched) of GSEA analysis with exhaustion/dysfunction signatures from Figure 4N.

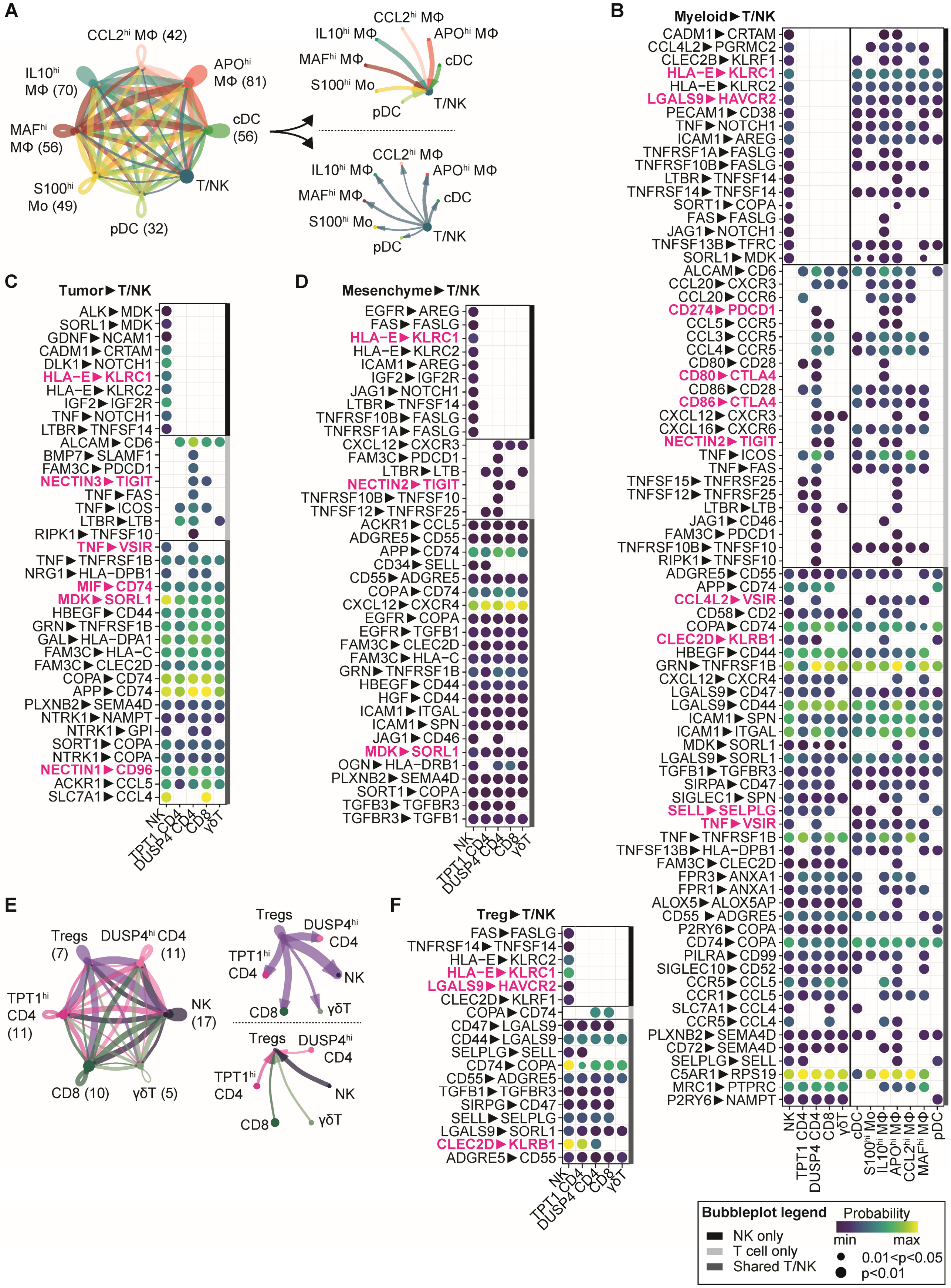
A
CCL2hi MΦ (42)
APOhi MΦ (81)
IL10hi MΦ (70)
MAFhi MΦ (56)
cDC (56)
S100hi Mo (49)
pDC (32)
T/NK
CCL2hi MΦ
IL10hi MΦ
APOhi MΦ
MAFhi MΦ
cDC
S100hi Mo
pDC
T/NK
B
Myeloid ▶ T/NK
CADM1 ▶ CRTAM
CCL4L2 ▶ PGRMC2
CLEC2B ▶ KLRF1
HLA−E ▶ KLRC1
HLA−E ▶ KLRC2
LGALS9 ▶ HAVCR2
PECAM1 ▶ CD38
TNF ▶ NOTCH1
ICAM1 ▶ AREG
TNFRSF1A ▶ FASLG
TNFRSF10B ▶ FASLG
LTBR ▶ TNFSF14
TNFRSF14 ▶ TNFSF14
SORT1 ▶ COPA
FAS ▶ FASLG
JAG1 ▶ NOTCH1
TNFSF13B ▶ TFRC
SORL1 ▶ MDK
ALCAM ▶ CD6
CCL20 ▶ CXCR3
CCL20 ▶ CCR6
CD274 ▶ PDCD1
CCL5 ▶ CCR5
CCL3 ▶ CCR5
CCL4 ▶ CCR5
CD80 ▶ CD28
CD80 ▶ CTLA4
CD86 ▶ CD28
CD86 ▶ CTLA4
CXCL12 ▶ CXCR3
CXCL16 ▶ CXCR6
NECTIN2 ▶ TIGIT
TNF ▶ ICOS
TNF ▶ FAS
TNFSF15 ▶ TNFRSF25
TNFSF12 ▶ TNFRSF25
LTBR ▶ LTB
JAG1 ▶ CD46
FAM3C ▶ PDCD1
TNFRSF10B ▶ TNFSF10
RIPK1 ▶ TNFSF10
ADGRE5 ▶ CD55
APP ▶ CD74
CCL4L2 ▶ VSIR
CD58 ▶ CD2
COPA ▶ CD74
CLEC2D ▶ KLRB1
HBEGF ▶ CD44
GRN ▶ TNFRSF1B
CXCL12 ▶ CXCR4
LGALS9 ▶ CD47
LGALS9 ▶ CD44
ICAM1 ▶ SPN
ICAM1 ▶ ITGAL
MDK ▶ SORL1
LGALS9 ▶ SORL1
TGFB1 ▶ TGFBR3
SIRPA ▶ CD47
SIGLEC1 ▶ SPN
SELL ▶ SELPLG
TNF ▶ VSIR
TNF ▶ TNFRSF1B
TNFSF13B ▶ HLA−DPB1
FAM3C ▶ CLEC2D
FPR3 ▶ ANXA1
FPR1 ▶ ANXA1
ALOX5 ▶ ALOX5AP
CD55 ▶ ADGRE5
P2RY6 ▶ COPA
CD74 ▶ COPA
PILRA ▶ CD99
SIGLEC10 ▶ CD52
CCR5 ▶ CCL5
CCR1 ▶ CCL5
SLC7A1 ▶ CCL4
CCR5 ▶ CCL4
PLXNB2 ▶ SEMA4D
CD72 ▶ SEMA4D
SELPLG ▶ SELL
C5AR1 ▶ RPS19
MRC1 ▶ PTPRC
P2RY6 ▶ NAMPT
NK
TPT1 CD4
DUSP4 CD4
CD8
γδT
cDC
S100hi Mo
IL10hi MΦ
APOhi MΦ
CCL2hi MΦ
MAFhi MΦ
pDC
C
Tumor ▶ T/NK
ALK ▶ MDK
SORL1 ▶ MDK
GDNF ▶ NCAM1
CADM1 ▶ CRTAM
DLK1 ▶ NOTCH1
HLA−E ▶ KLRC1
HLA−E ▶ KLRC2
IGF2 ▶ IGF2R
TNF ▶ NOTCH1
LTBR ▶ TNFSF14
ALCAM ▶ CD6
BMP7 ▶ SLAMF1
FAM3C ▶ PDCD1
NECTIN3 ▶ TIGIT
TNF ▶ FAS
TNF ▶ ICOS
LTBR ▶ LTB
RIPK1 ▶ TNFSF10
TNF ▶ VSIR
TNF ▶ TNFRSF1B
NRG1 ▶ HLA−DPB1
MIF ▶ CD74
MDK ▶ SORL1
HBEGF ▶ CD44
GRN ▶ TNFRSF1B
GAL ▶ HLA−DPA1
FAM3C ▶ HLA−C
FAM3C ▶ CLEC2D
COPA ▶ CD74
APP ▶ CD74
PLXNB2 ▶ SEMA4D
NTRK1 ▶ NAMPT
NTRK1 ▶ GPI
SORT1 ▶ COPA
NTRK1 ▶ COPA
NECTIN1 ▶ CD96
ACKR1 ▶ CCL5
SLC7A1 ▶ CCL4
NK
TPT1 CD4
DUSP4 CD4
CD8
γδT
D
Mesenchyme ▶ T/NK
EGFR ▶ AREG
FAS ▶ FASLG
HLA−E ▶ KLRC1
HLA−E ▶ KLRC2
ICAM1 ▶ AREG
IGF2 ▶ IGF2R
JAG1 ▶ NOTCH1
LTBR ▶ TNFSF14
TNFRSF10B ▶ FASLG
TNFRSF1A ▶ FASLG
CXCL12 ▶ CXCR3
FAM3C ▶ PDCD1
LTBR ▶ LTB
NECTIN2 ▶ TIGIT
TNFRSF10B ▶ TNFSF10
TNFSF12 ▶ TNFRSF25
ACKR1 ▶ CCL5
ADGRE5 ▶ CD55
APP ▶ CD74
CD34 ▶ SELL
CD55 ▶ ADGRE5
COPA ▶ CD74
CXCL12 ▶ CXCR4
EGFR ▶ COPA
EGFR ▶ TGFB1
FAM3C ▶ CLEC2D
FAM3C ▶ HLA−C
GRN ▶ TNFRSF1B
HBEGF ▶ CD44
HGF ▶ CD44
ICAM1 ▶ ITGAL
ICAM1 ▶ SPN
JAG1 ▶ CD46
MDK ▶ SORL1
OGN ▶ HLA−DRB1
PLXNB2 ▶ SEMA4D
SORT1 ▶ COPA
TGFB3 ▶ TGFBR3
TGFBR3 ▶ TGFB1
NK
TPT1 CD4
DUSP4 CD4
CD8
γδT
E
Tregs (7)
DUSP4hi CD4 (11)
TPT1hi CD4 (11)
NK (17)
CD8 (10)
γδT (5)
Tregs
DUSP4hi CD4
TPT1hi CD4
NK
CD8
γδT
F
Treg ▶ T/NK
FAS ▶ FASLG
TNFRSF14 ▶ TNFSF14
HLA−E ▶ KLRC2
HLA−E ▶ KLRC1
LGALS9 ▶ HAVCR2
CLEC2D ▶ KLRF1
COPA ▶ CD74
CD47 ▶ LGALS9
CD44 ▶ LGALS9
SELPLG ▶ SELL
CD74 ▶ COPA
CD55 ▶ ADGRE5
TGFB1 ▶ TGFBR3
SIRPG ▶ CD47
SELL ▶ SELPLG
LGALS9 ▶ SORL1
CLEC2D ▶ KLRB1
ADGRE5 ▶ CD55
NK
TPT1 CD4
DUSP4 CD4
CD8
γδT
Bubbleplot legend
NK only
T cell only
Shared T/NK
Probability
min
max
0.01<p<0.05
p<0.01

Figure S6. Immunoregulatory interactions in the neuroblastoma tumor-microenvironment, related to figure 5. (A) Interaction network involving interactions between myeloid cells and T/NK cells constructed with CellChat[9]. **(B)** Bubbleplot of predicted interactions between myeloid cells and indicated T/NK subsets. Interactions with each specific T/NK subset and each specific myeloid subset were evaluated and subsequently merged, with the highest probability of each interaction pair depicted in the plot (*left: myeloid merged; right: T/NK merged*) **(C)** Bubbleplot of predicted interactions between tumor cells and indicated T/NK subsets. **(D)** Bubbleplot of predicted interactions between mesenchymal cells and indicated T/NK subsets. **(E)** Interaction network of interactions among Tregs and all other T/NK cell subsets. **(F)** Bubbleplot involving interactions between Tregs and indicated T/NK cell subsets.

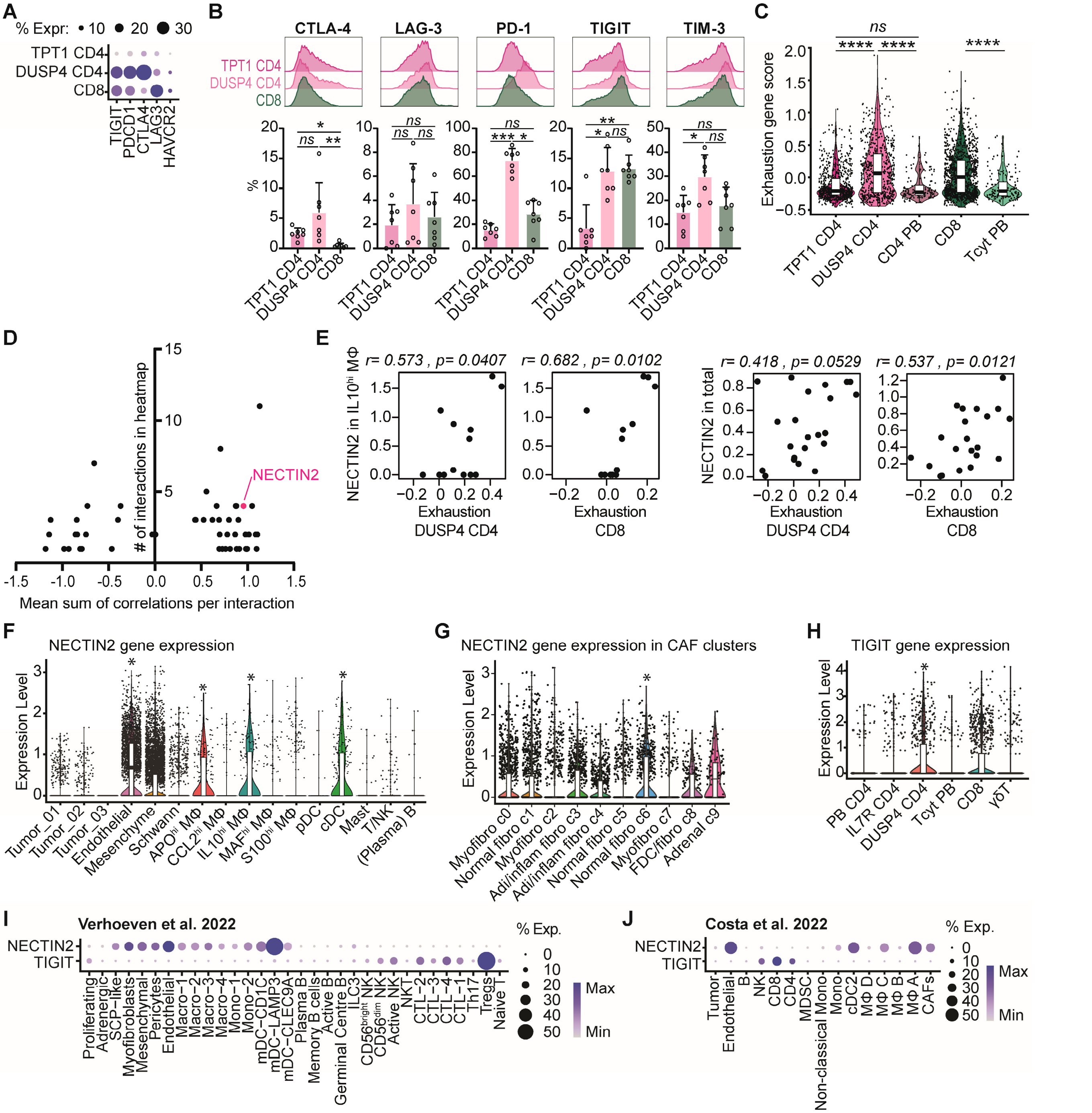

Figure S7. The *NECTIN2-TIGIT* axis is associated with T cell dysfunction, related to figure 6. (A) Dotplot with expression of dysfunction/exhaustion markers *CTLA4*, *LAG3*, *TIGIT*, *PDCD1* and *HAVCR2* in the indicated T cell subsets. **(B)** Flow cytometric analysis of CTLA-4, LAG-3, TIGIT, PD-1 and TIM-3 expression on neuroblastoma-infiltrating T cell subsets. TPT1^{hi} CD4 were gated as IL-7R^{hi}PD-1^{lo} CD4^{+} cells and DUSP4^{hi} CD4 were gated as IL-7R^{lo}PD-1^{hi} CD4^{+} cells, as shown in figure 3F. *Kruskall Wallis + Dunn's. *P<0.05, **P<0.01, ***P<0.001, ns=not significant.* **(C)** Modulescore of the dysfunction/exhaustion genes in the indicated T cell subsets. *Kruskall Wallis + Dunn's. ****P<0.0001*. **(D)** NECTIN2 'ranking' among genes "B" from figure 6B which correlate positively with dysfunction score of T cells, showing the mean sum of correlations of all interactions involving gene "B" in figure 6B, and the total number of interactions involving gene "B" in figure 6B. **(E)** Correlation between *NECTIN2* expression in IL10hi Mϕ and total cells in the TME with exhaustion scores of *DUSP4*^{hi} CD4 and CD8 T cells. **(F)** NECTIN2 gene expression in neuroblastoma TME. **P<0.0001* from *Findallmarkers* analysis. **(G)** NECTIN2 gene expression in detailed mesenchyme clusters. **P<0.0001* from *Findallmarkers* analysis. **(H)** TIGIT gene expressing on T cells infiltrating neuroblastoma compared to T cells from healthy donor blood. **P<0.0001* from *Findallmarkers* analysis. **(I)** TIGIT and NECTIN2 expression in dataset of Verhoeven et al.[5] **(J)** TIGIT and NECTIN2 expression in dataset of Costa et al.[4]

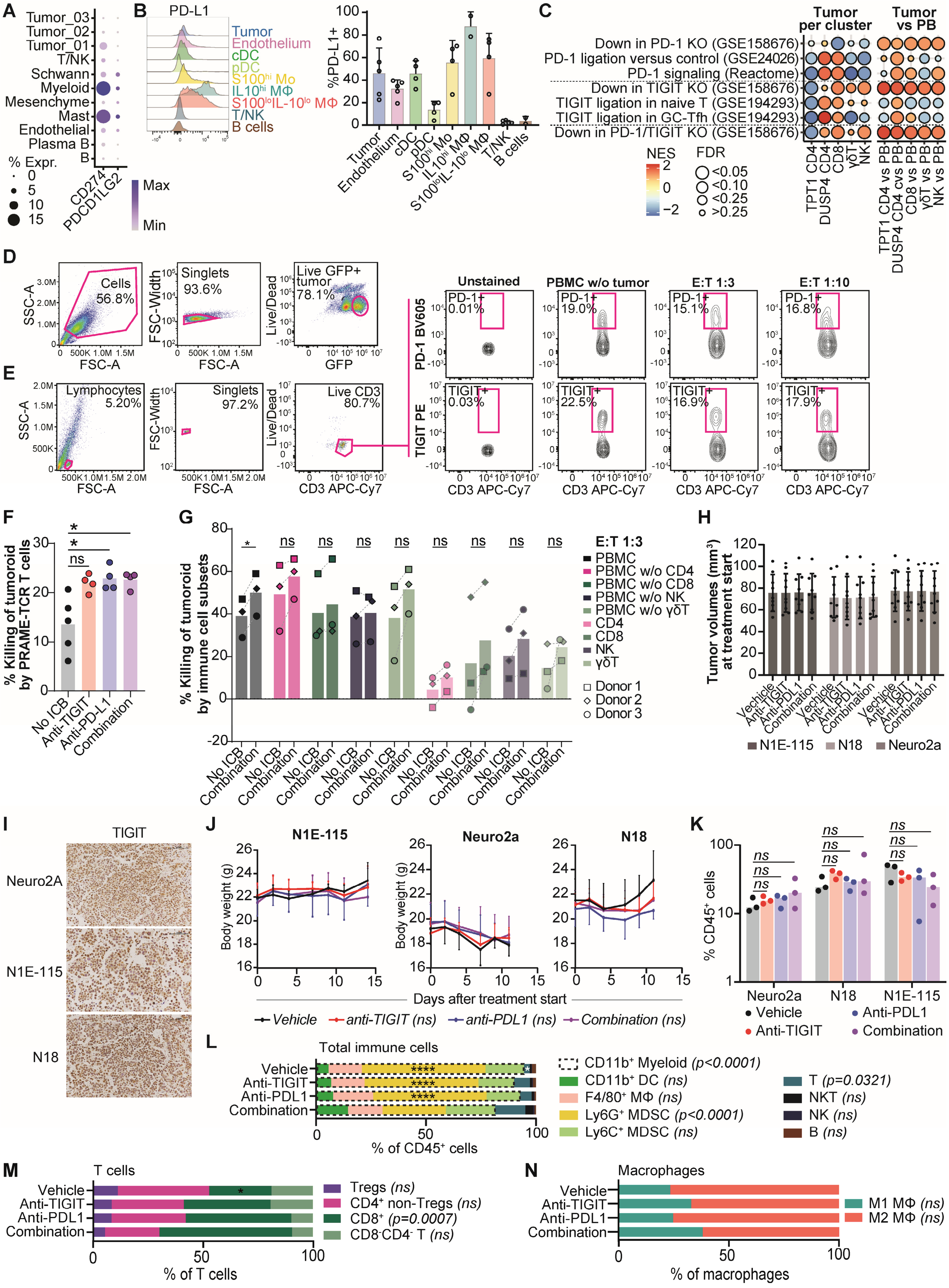

A
Tumor_03
Tumor_02
Tumor_01
T/NK
Schwann
Myeloid
Mesenchyme
Mast
Endothelial
Plasma B
B
% Expr.
0
5
10
15
CD274
PDCD1LG2
Max
Min
B
PD-L1
Tumor
Endothelium
cDC
pDC
S100hi Mo
IL10hi MΦ
S100loIL-10lo MΦ
T/NK
B cells
%PD-L1+
C
Tumor per cluster
Tumor vs PB
Down in PD-1 KO (GSE158676)
PD-1 ligation versus control (GSE24026)
PD-1 signaling (Reactome)
Down in TIGIT KO (GSE158676)
TIGIT ligation in naive T (GSE194293)
TIGIT ligation in GC-Tfh (GSE194293)
Down in PD-1/TIGIT KO (GSE158676)
NES
FDR
<0.05
<0.10
<0.25
>0.25
TPT1 CD4
DUSP4 CD4
CD8
γδT
NK
TPT1 CD4 vs PB
DUSP4 CD4 cvs PB
CD8 vs PB
γδT vs PB
NK vs PB
D
Cells 56.8%
Singlets 93.6%
Live GFP+ tumor 78.1%
SSC-A
FSC-A
FSC-Width
Live/Dead
GFP
Unstained
PBMC w/o tumor
E:T 1:3
E:T 1:10
PD-1+ 0.01%
PD-1+ 19.0%
PD-1+ 15.1%
PD-1+ 16.8%
PD-1 BV605
E
Lymphocytes 5.20%
Singlets 97.2%
Live CD3 80.7%
CD3 APC-Cy7
TIGIT+ 0.03%
TIGIT+ 22.5%
TIGIT+ 16.9%
TIGIT+ 17.9%
TIGIT PE
F
% Killing of tumoroid by PRAME-TCR T cells
ns
No ICB
Anti-TIGIT
Anti-PD-L1
Combination
G
% Killing of tumoroid by immune cell subsets
E:T 1:3
PBMC
PBMC w/o CD4
PBMC w/o CD8
PBMC w/o NK
PBMC w/o γδT
CD4
CD8
NK
γδT
Donor 1
Donor 2
Donor 3
No ICB
Combination
H
Tumor volumes (mm³) at treatment start
Vechicle
Anti-TIGIT
Anti-PDL1
Combination
N1E-115
N18
Neuro2a
I
TIGIT
Neuro2A
N1E-115
N18
J
N1E-115
Neuro2a
N18
Body weight (g)
Days after treatment start
Vehicle
anti-TIGIT (ns)
anti-PDL1 (ns)
Combination (ns)
K
% CD45+ cells
Neuro2a
N18
N1E-115
Vehicle
Anti-TIGIT
Anti-PDL1
Combination
L
Total immune cells
Vehicle
Anti-TIGIT
Anti-PDL1
Combination
% of CD45+ cells
CD11b+ Myeloid (p<0.0001)
CD11b+ DC (ns)
F4/80+ MΦ (ns)
Ly6G+ MDSC (p<0.0001)
Ly6C+ MDSC (ns)
T (p=0.0321)
NKT (ns)
NK (ns)
B (ns)
M
T cells
Tregs (ns)
CD4+ non-Tregs (ns)
CD8+ (p=0.0007)
CD8-CD4- T (ns)
% of T cells
N
Macrophages
M1 MΦ (ns)
M2 MΦ (ns)
% of macrophages

Figure S8. Combined TIGIT/PD-L1 blockade enhances immune responses against neuroblastoma *in vitro* and *in vivo*, related to figure 7. (A) DotPlot with expression of PD-1 ligands *CD274* (PD-L1) and *PDCD1LG2* (PD-L2) in neuroblastoma. **(B)** Flow cytometric analysis of PD-L1 expression in the neuroblastoma tumor-microenvironment. **(C)** Gene set enrichment analysis of selected signatures for PD-1 and/or TIGIT downstream signaling. KO=knock-out, GC-Tfh=germinal center follicular helper T cells. NES=normalized enrichment score. **(D)** Gating strategy for flow cytometric expression analysis of Nectin-2 and PD-L1 expression on GFP-positive tumoroids as shown in Figure 7C. **(E)** Flow cytometric analysis of PD-1 and TIGIT expression on healthy donor PBMC cocultured with neuroblastoma tumoroids for 6 days. 'PBMC w/o tumor' was measured at timepoint 0 before coculture. **(F)** Percentage of tumoroid (AMC691T) killing (=100-normalized luminescence) *in vitro* after 6 days of co-culture with PRAME-TCR transduced T cells at E:T ratio 1:300. Luminescence values were normalized against condition with untreated tumoroids only. n=4-5 technical replicates. *Kruskall-Wallis with Dunn's.*$p<0.05$; ns=not significant.* **(G)** Percentage of tumoroid (AMC691T) killing (=100-normalized luminescence) *in vitro* after 6 days of co-culture with FACS-sorted immune subsets from healthy donor peripheral blood at E:T ratio 1:3. Luminescence values were normalized against condition with untreated tumoroids only. n=3 donors. *Two-way ANOVA with Sidak*. *$p<0.05$; ns=not significant. w/o=without.* **(H)** Tumor volumes at treatment start per condition for the three *in vivo* models. **(I)** Immunohistochemical staining of TIGIT, PD-1 and PD-L1 in the three *in vivo* models (vehicle condition). **(J)** Body weight of N1E-115, Neuro2a and N18 mouse models (n=6 per group) treated with anti-TIGIT and/or anti-PD-L1 up to day 15 of treatment. **(K-N)** Flow cytometric analysis of the TME *in vivo* in n=3 mice per treatment condition, treated with anti-TIGIT and/or anti-PD-L1 for 7 days. **(K)** Percentage of CD45+ immune cells of total live cells. **(L)** Total immune cell composition across treatments. Models were pooled. **$P<0.05$ versus combination, ****$P<0.0001$ versus combination. 2-Way ANOVA.* **(M)** T cell composition across treatments. Models were pooled. **$P<0.05$ versus combination. 2-Way ANOVA.* **(N)** Macrophage composition across treatments. Models were pooled. *2-Way ANOVA.*

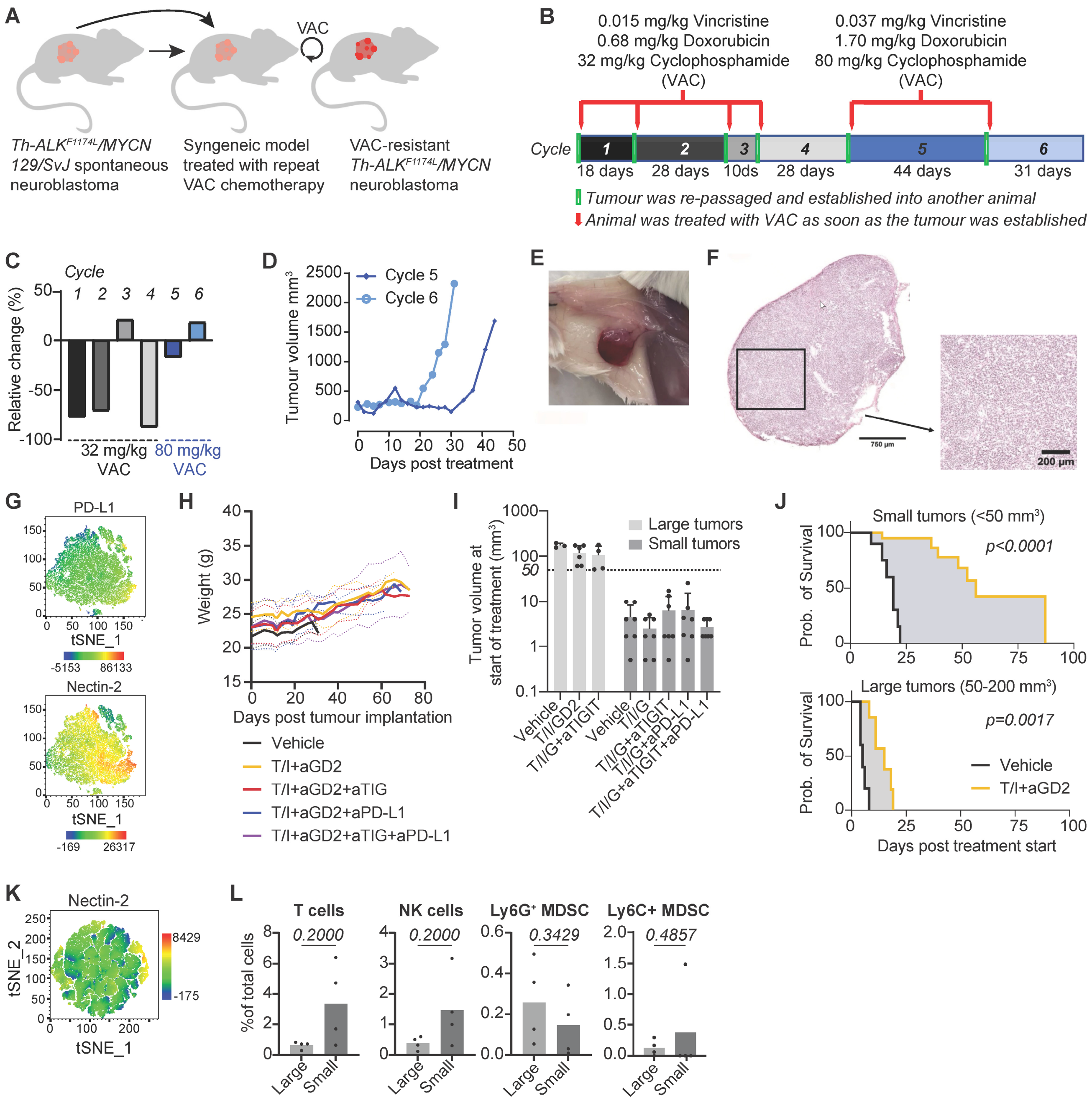

Figure S9. TIGIT blockade improves survival in a chemotherapy-resistant neuroblastoma model, related to figure 8. (A) Generation of chemotherapy-resistant neuroblastoma model from *Th-ALK*^{F1174L}/*MYCN* 129/SvJ models repeatedly treated with VAC (Vincristine, Adriamycin/Doxorubicin, Cyclophosphamide). **(B)** Summary of dose escalation treatment schedule to achieve resistance. Red arrows indicate when single dose VAC was administered. The number of days taken for the tumor to relapse is indicated below each cycle. At the end of each cycle, the allograft tumor was re-passaged into another animal before treatment started. **(C)** Percentage relative tumor volume change 7 days after treatment with single dose of VAC at 32 mg/kg or 80 mg/kg. **(D)** Time taken for allograft tumor to relapse after treatment with single high dose of VAC (80 mg/kg VAC) in Cycle 5 and Cycle 6. Tumor cells after Cycle 6 were used to establish allografts for therapeutic studies. **(E)** Macroscopic confirmation of tumor presence for small tumors. **(F)** Histological confirmation of tumor cell presence for small tumors. **(G)** TSNE representation of flow cytometric analysis of PD-L1 and Nectin-2 expression in the TME of small tumors (vehicle condition). **(H)** Body weight of mice with small tumors (n=7 per group) treated with TEM/IRI, anti-GD2 and/or anti-TIGIT and/or anti-PD-L1 up to day 80 of treatment. **(I)** Tumor volumes at start of treatment per treatment condition for mice with large and small tumors. **(J)** Survival analysis comparing response to TEM/IRI (T/I) +anti-GD2 (aGD2) in small and large tumors. **(K)** TSNE representation of flow cytometric analysis of Nectin-2 expression in the TME of large tumors (vehicle condition). **(L)** Flow cytometric analysis of immune cell subsets as a fraction of total live cells in the tumor. *Mann Whitney U test. N=4 animals per condition.*

References

1. Luo, H., Xia, X., Huang, L.-B., An, H., Cao, M., Kim, G.D., Chen, H.-N., Zhang, W.-H., Shu, Y., Kong, X., et al. (2022). Pan-cancer single-cell analysis reveals the heterogeneity and plasticity of cancer-associated fibroblasts in the tumor microenvironment. Nat. Commun. *13*, 6619. 10.1038/s41467-022-34395-2.
2. Bedoya-Reina, O.C., Li, W., Arceo, M., Plescher, M., Bullova, P., Pui, H., Kaucka, M., Kharchenko, P., Martinsson, T., Holmberg, J., et al. (2021). Single-nuclei transcriptomes from human adrenal gland reveal distinct cellular identities of low and high-risk neuroblastoma tumors. Nat. Commun. *12*, 5309. 10.1038/s41467-021-24870-7.
3. Abe, Y., Sakata-Yanagimoto, M., Fujisawa, M., Miyoshi, H., Suehara, Y., Hattori, K., Kusakabe, M., Sakamoto, T., Nishikii, H., Nguyen, T.B., et al. (2022). A single-cell atlas of non-haematopoietic cells in human lymph nodes and lymphoma reveals a landscape of stromal remodelling. Nat. Cell Biol. *24*, 565–578. 10.1038/s41556-022-00866-3.
4. Costa, A., Thirant, C., Kramdi, A., Pierre-Eugène, C., Louis-Brennetot, C., Blanchard, O., Surdez, D., Gruel, N., Lapouble, E., Pierron, G., et al. (2022). Single-cell transcriptomics reveals shared immunosuppressive landscapes of mouse and human neuroblastoma. J. Immunother. cancer *10*. 10.1136/jitc-2022-004807.
5. Verhoeven, B.M., Mei, S., Olsen, T.K., Gustafsson, K., Valind, A., Lindström, A., Gisselsson, D., Fard, S.S., Hagerling, C., Kharchenko, P. V, et al. (2022). The immune cell atlas of human neuroblastoma. Cell reports. Med. *3*, 100657. 10.1016/j.xcrm.2022.100657.
6. Karlsson, G., Liu, Y., Larsson, J., Goumans, M.-J., Lee, J.-S., Thorgeirsson, S.S., Ringnér, M., and Karlsson, S. (2005). Gene expression profiling demonstrates that TGF-beta1 signals exclusively through receptor complexes involving Alk5 and identifies targets of TGF-beta signaling. Physiol. Genomics *21*, 396–403. 10.1152/physiolgenomics.00303.2004.
7. Man, K., Gabriel, S.S., Liao, Y., Gloury, R., Preston, S., Henstridge, D.C., Pellegrini, M., Zehn, D., Berberich-Siebelt, F., Febbraio, M.A., et al. (2017). Transcription Factor IRF4 Promotes CD8(+) T Cell Exhaustion and Limits the Development of Memory-like T Cells during Chronic Infection. Immunity *47*, 1129-1141.e5. 10.1016/j.immuni.2017.11.021.
8. Li, H., van der Leun, A.M., Yofe, I., Lubling, Y., Gelbard-Solodkin, D., van Akkooi, A.C.J., van den Braber, M., Rozeman, E.A., Haanen, J.B.A.G., Blank, C.U., et al. (2019). Dysfunctional CD8 T Cells Form a Proliferative, Dynamically Regulated Compartment within Human Melanoma. Cell *176*, 775-789.e18. 10.1016/j.cell.2018.11.043.
9. Jin, S., Guerrero-Juarez, C.F., Zhang, L., Chang, I., Ramos, R., Kuan, C.-H., Myung, P., Plikus, M. V, and Nie, Q. (2021). Inference and analysis of cell-cell communication using CellChat. Nat. Commun. *12*, 1088. 10.1038/s41467-021-21246-9.
