# Supplementary material for: Integrative analysis of neuroblastoma by single-cell RNA sequencing identifies the NECTIN2-TIGIT axis as a target for immunotherapy
Source: Cancer Cell. 2024 Feb 12;42(2):283–300.e8. doi: 10.1016/j.ccell.2023.12.008 (PMC10864003; doi:10.1016/j.ccell.2023.12.008)
Supplement: Document S2. Article plus supplemental information [file mmc4.pdf]

Cancer Cell

Article

# Integrative analysis of neuroblastoma by single-cell RNA sequencing identifies the NECTIN2-TIGIT axis as a target for immunotherapy

## Graphical abstract

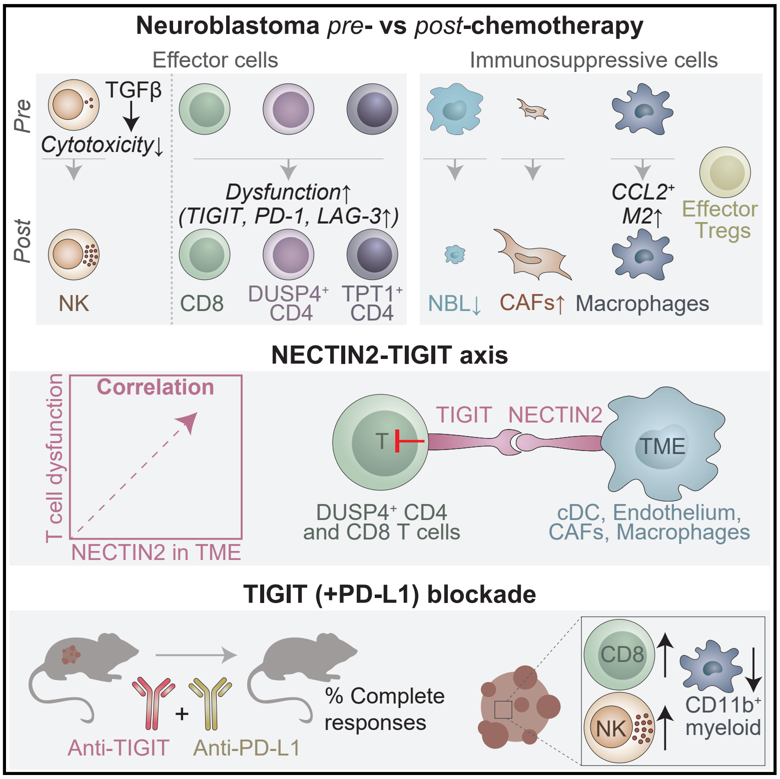


## Highlights

- Single-cell RNAseq identifies 17 distinct immune subsets infiltrating neuroblastoma
- Dysfunctionality of T and NK cells alternates throughout the course of chemotherapy
- Interaction analysis identifies the NECTIN2-TIGIT axis as crucial immune checkpoint
- TIGIT (+PD-L1) blockade enhances immune responses against neuroblastoma *in vivo*

## Authors

Judith Wienke, Lindy L. Visser, Waleed M. Kholosy, ..., Thanasis Margaritis, Max M. van Noesel, Jan J. Molenaar

## Correspondence

j.wienke-4@prinsesmaximacentrum.nl

## In brief

Wienke et al. analyze the immune landscape of neuroblastoma pre- and post-chemotherapy and identify the NECTIN2-TIGIT axis as a crucial immune checkpoint, which correlates with dysfunction of T/NK cells. TIGIT (+PD-L1) blockade induces numerous complete responses (CR) *in vivo,* even against chemotherapy-resistant neuroblastoma, highlighting TIGIT blockade as promising immunotherapy for neuroblastoma.

Wienke et al., 2024, Cancer Cell *42*, 283–300
February 12, 2024 © 2023 The Authors. Published by Elsevier Inc.
https://doi.org/10.1016/j.ccell.2023.12.008

CellPress

Cancer Cell

CellPress
OPEN ACCESS

Article

# Integrative analysis of neuroblastoma by single-cell RNA sequencing identifies the NECTIN2-TIGIT axis as a target for immunotherapy

Judith Wienke,[1,29,*] Lindy L. Visser,[1,28] Waleed M. Kholosy,[1,28] Kaylee M. Keller,[1,28] Marta Barisa,[2] Evon Poon,[3] Sophie Munnings-Tomes,[2] Courtney Himsworth,[2] Elizabeth Calton,[3] Ana Rodriguez,[4] Ronald Bernardi,[5] Femke van den Ham,[1] Sander R. van Hooff,[1] Yvette A.H. Matser,[1] Michelle L. Tas,[1] Karin P.S. Langenberg,[1] Philip Lijnzaad,[1] Anne L. Borst,[1] Elisa Zappa,[1] Francisca J. Bergsma,[1] Josephine G.M. Strijker,[1] Bronte M. Verhoeven,[6] Shenglin Mei,[7] Amira Kramdi,[8,9] Restuadi Restuadi,[10,11] Alvaro Sanchez-Bernabeu,[12,13] Annelisa M. Cornel,[1,14] Frank C.P. Holstege,[1] Juliet C. Gray,[15] Godelieve A.M. Tytgat,[1] Marijn A. Scheijde-Vermeulen,[1] Marc H.W.A. Wijnen,[1] Miranda P. Dierselhuis,[1] Karin Straathof,[16,17] Sam Behjati,[18,19,20] Wei Wu,[12,13,21,22] Albert J.R. Heck,[12,13] Jan Koster,[23] Stefan Nierkens,[1,14] Isabelle Janoueix-Lerosey,[8,9] Ronald R. de Krijger,[1,24] Ninib Baryawno,[6] Louis Chesler,[3] John Anderson,[2,25] Hubert N. Caron,[4] Thanasis Margaritis,[1] Max M. van Noesel,[1,26] and Jan J. Molenaar[1,27]
[1]Princess Máxima Center for Pediatric Oncology, Utrecht, the Netherlands
[2]Cancer Section, Developmental Biology and Cancer Programme, UCL Great Ormond Street Institute of Child Health, London, UK
[3]Division of Clinical Studies, The Institute of Cancer Research, London, UK
[4]Hoffman-La Roche, Basel, Switzerland
[5]Genentech, A Member of the Roche Group, South San Francisco, CA, USA
[6]Childhood Cancer Research Unit, Department of Women's and Children's Health, Karolinska Institutet, Stockholm, Sweden
[7]Department of Biomedical Informatics, Harvard Medical School, Boston, MA 02115, USA
[8]Institut Curie, Inserm U830, PSL Research University, Diversity and Plasticity of Childhood Tumors Lab, Paris, France
[9]SIREDO: Care, Innovation and Research for Children, Adolescents and Young Adults with Cancer, Institut Curie, Paris, France
[10]Infection, Immunity and Inflammation Research and Teaching Department, UCL Great Ormond Street Institute of Child Health, London, UK
[11]NIHR Biomedical Research Centre, Great Ormond Street Hospital, London, UK
[12]Biomolecular Mass Spectrometry and Proteomics, Bijvoet Center for Biomolecular Research and Utrecht Institute for Pharmaceutical Sciences, Utrecht University, Utrecht, the Netherlands
[13]Netherlands Proteomics Centre, Utrecht University, Utrecht, the Netherlands
[14]Center for Translational Immunology, University Medical Center Utrecht, Utrecht, the Netherlands
[15]Centre for Cancer Immunology, University of Southampton, Southampton, UK
[16]University College London (UCL) Great Ormond Street Institute of Child Health, London, UK
[17]UCL Cancer Institute, London, UK
[18]Wellcome Sanger Institute, Hinxton, UK
[19]Cambridge University Hospitals NHS Foundation Trust, Cambridge, UK
[20]Department of Paediatrics, University of Cambridge, Cambridge, UK
[21]Singapore Immunology Network (SIgN), Agency for Science, Technology and Research (A*STAR), Singapore, Singapore
[22]Department of Pharmacy, National University of Singapore, Singapore, Singapore
[23]Amsterdam UMC location University of Amsterdam, Center for Experimental and Molecular Medicine, Amsterdam, the Netherlands
[24]Department of Pathology, University Medical Center Utrecht, Utrecht, the Netherlands
[25]Department of Oncology, Great Ormond Street Hospital for Children NHS Foundation Trust, London, England, UK
[26]Division Imaging & Cancer, University Medical Center Utrecht, Utrecht, the Netherlands
[27]Department of Pharmaceutical Sciences, Utrecht University, Utrecht, the Netherlands
[28]These authors contributed equally
[29]Lead contact

## SUMMARY

Pediatric patients with high-risk neuroblastoma have poor survival rates and urgently need more effective treatment options with less side effects. Since novel and improved immunotherapies may fill this need, we dissect the immunoregulatory interactions in neuroblastoma by single-cell RNA-sequencing of 24 tumors (10 pre- and 14 post-chemotherapy, including 5 pairs) to identify strategies for optimizing immunotherapy efficacy. Neuroblastomas are infiltrated by natural killer (NK), T and B cells, and immunosuppressive myeloid populations. NK cells show reduced cytotoxicity and T cells have a dysfunctional profile. Interaction analysis reveals a vast immunoregulatory network and identifies NECTIN2-TIGIT as a crucial immune checkpoint. Combined blockade of TIGIT and PD-L1 significantly reduces neuroblastoma growth, with complete responses (CR) *in vivo*. Moreover, addition of TIGIT+PD-L1 blockade to standard relapse treatment in a chemotherapy-resistant *Th-ALK*$^{F1174L}$/*MYCN* 129/SvJ syngeneic model induces CR. In conclusion, our integrative analysis provides promising targets and a rationale for immunotherapeutic combination strategies.


This is an open access article under the CC BY license (http://creativecommons.org/licenses/by/4.0/).

## INTRODUCTION

Immunotherapy has revolutionized cancer treatment in adults and holds great promise for pediatric solid tumors.[1] Its potential is exemplified by the increased survival of patients with high-risk neuroblastoma following implementation of anti-GD2 antibody therapy into standard care.[2,3] Neuroblastoma, the most common extracranial pediatric solid tumor, accounts for 10% of pediatric cancer-related deaths.[4] Patients are stratified into risk groups based on disease presentation and genomic alterations like *MYCN* amplification, which is an important driver of poor prognosis.[5] High-risk neuroblastoma patients receive an intense multimodal treatment regimen, consisting of induction chemotherapy, surgical tumor resection, consolidation with high-dose chemotherapy followed by autologous stem cell transplantation, radiotherapy and anti-GD2 immunotherapy. Introduction of anti-GD2 has improved high-risk neuroblastoma event-free survival rates by ~15%.[2,3] Still, overall 5-year survival rates are below 60%, particularly due to the high relapse rate.[3] Chemoimmunotherapy with temozolomide, irinotecan and anti-GD2 is likely to be selected as the backbone treatment for relapse/refractory neuroblastoma patients in Europe.[6] Due to the intense treatment, the majority of survivors suffer of debilitating (long-term) side effects.[7] Taken together, there is an urgent need for more effective treatments with less side effects.

The success of anti-GD2 therapy has provided a clear rationale for immunotherapy in neuroblastoma treatment. Yet, as the survival benefit is still modest, improving immunotherapy efficacy will be crucial. T cells and natural killer (NK) cells are considered essential effectors in the context of immunotherapy, but their function is compromised in many cancers.[8,9] Efforts to improve immunotherapy efficacy focus on reinvigorating T and NK cell function, e.g., with immune checkpoint inhibition or by introducing cellular immunotherapies like chimeric antigen receptor (CAR) T cells.[1,10] Until recently, these approaches showed relatively limited efficacy in clinical trials for neuroblastoma, but the recent CAR-T trial by Del Bufalo et al. provided promising results.[11–20] The overall limited success may be due to intra-tumoral factors hampering efficacy, such as immunosuppressive cells and a plethora of immune checkpoints.[10,21] Efforts to optimize immunotherapies for neuroblastoma are currently hampered by a lack of information on these intra-tumoral factors. The detailed composition and function of T and NK cells in the neuroblastoma tumor-microenvironment (TME) is still largely unexplored and only few immunosuppressive factors have so far been identified.[22] To guide further immunotherapy development—and enhancement—it is essential to gain better insights into neuroblastoma's immune environment and the immunoregulatory mechanisms at play. Moreover, such insights may fuel an educated approach to design combination immunotherapy strategies overcoming immune resistance.[22]

To unravel the immune environment of neuroblastoma and identify targets for immunotherapy enhancement, we generated a single-cell transcriptomic atlas of 24 tumors, with samples taken before and after induction chemotherapy. We provide a detailed view of neuroblastoma's immune landscape pre- and post-chemotherapy and reveal a vast immunoregulatory interaction network. Subsequent functional validation experiments identified the NECTIN2-TIGIT axis as promising target for neuroblastoma immunotherapy.

## RESULTS

### The single-cell landscape of neuroblastoma

To provide an in-depth view of neuroblastoma's TME, tumor samples were analyzed by single-cell RNA sequencing. Fresh material was processed from 24 tumors of 19 patients, 10 taken pre-treatment and 14 after induction chemotherapy, including five paired samples. Seventeen patients had high-risk neuroblastoma and two had intermediate risk neuroblastoma. Six out of 19 patients had *MYCN* amplified (*MYCN*-A) tumors (Figure 1A; Table S1). Single-cell RNA sequencing using the Cel-Seq2 protocol yielded 22,418 high-quality cells. We identified 24 cell clusters, which were annotated as five main cell types, i.e., tumor, immune cells, endothelium, mesenchyme, and Schwann cell precursor cells (Figures 1B, 1C, and S1A), confirming previous findings.[23,24] The three tumor clusters highly expressed *CDK4*, *CCND1*, and *MYCN,* respectively (Figure 1C). Cluster 1 contained *CDK4* amplified cells and cluster 3 contained *MYCN*-A cells (Table S1). In addition, the tumor clusters expressed previously described neuroblastoma markers genes, including *CHGB*, *PHOX2B*, and *TH* (Figures 1D and S1B).[25] Tumor cell identity was confirmed by copy number variation inference, showing typical chromosomal aberrations (e.g., 1p loss, 11q loss, and 17q gain; Figure S1C).[25] HLA class I expression was significantly lower in tumor than non-tumor cells, particularly in those with high *MYCN* expression (Figures S1D–S1F), highlighting neuroblastoma's low immunogenicity.[26] Nonetheless, the cellular (immune) fractions of *MYCN*-A tumors did not significantly differ from *MYCN*-non amplified tumors (Figure S1G). Distinct changes in the cellular composition were observed upon induction chemotherapy (Figures 1E–1G). The tumor content decreased from 47% (range 9–96%) pre-treatment to 3% (0–20%) post-treatment, while the mesenchymal fraction increased from 11% (0–43%) to 49% (1–87%) (both $p < 0.05$, Figures 1F and 1G). The mesenchymal fraction consisted of (cancer-associated) fibroblasts (CAF) with phenotypes ranging from myofibroblasts to inflammatory/adipogenic fibroblasts, one population enriched for follicular dendritic cells (FDC) derived from lymph node biopsies (C8), and one population of healthy adrenal cells (C9; Figures S1H–S1J). CAFs displayed functional and compositional differences before and after treatment (Figures S1I–S1K). C0 and C8, containing myofibroblasts and inflammatory CAF/FDC, were the most prominent CAF clusters pre-treatment, while clusters C2, C5, and C6 were more enriched post-treatment. The other main cell fractions, including immune cells, did not change upon treatment (Figure 1F).

### Neuroblastoma is populated by immunosuppressive macrophages

To unravel the composition and function of neuroblastoma-infiltrating immune cells we performed an in-depth analysis of the *PTPRC*$^{+}$ (CD45$^{+}$) immune clusters. Among a total of 6,012 immune cells, we identified myeloid and lymphoid populations (Figures 2A, S2A, and S2B). The myeloid cells, key regulators of anti-tumor immunity due to their roles in antigen presentation, T cell polarization and immunosuppression,[27] consisted of mast cells, plasmacytoid dendritic cells (pDC), conventional DC (cDC), *S100A8/A9*$^{hi}$

CellPress
OPEN ACCESS

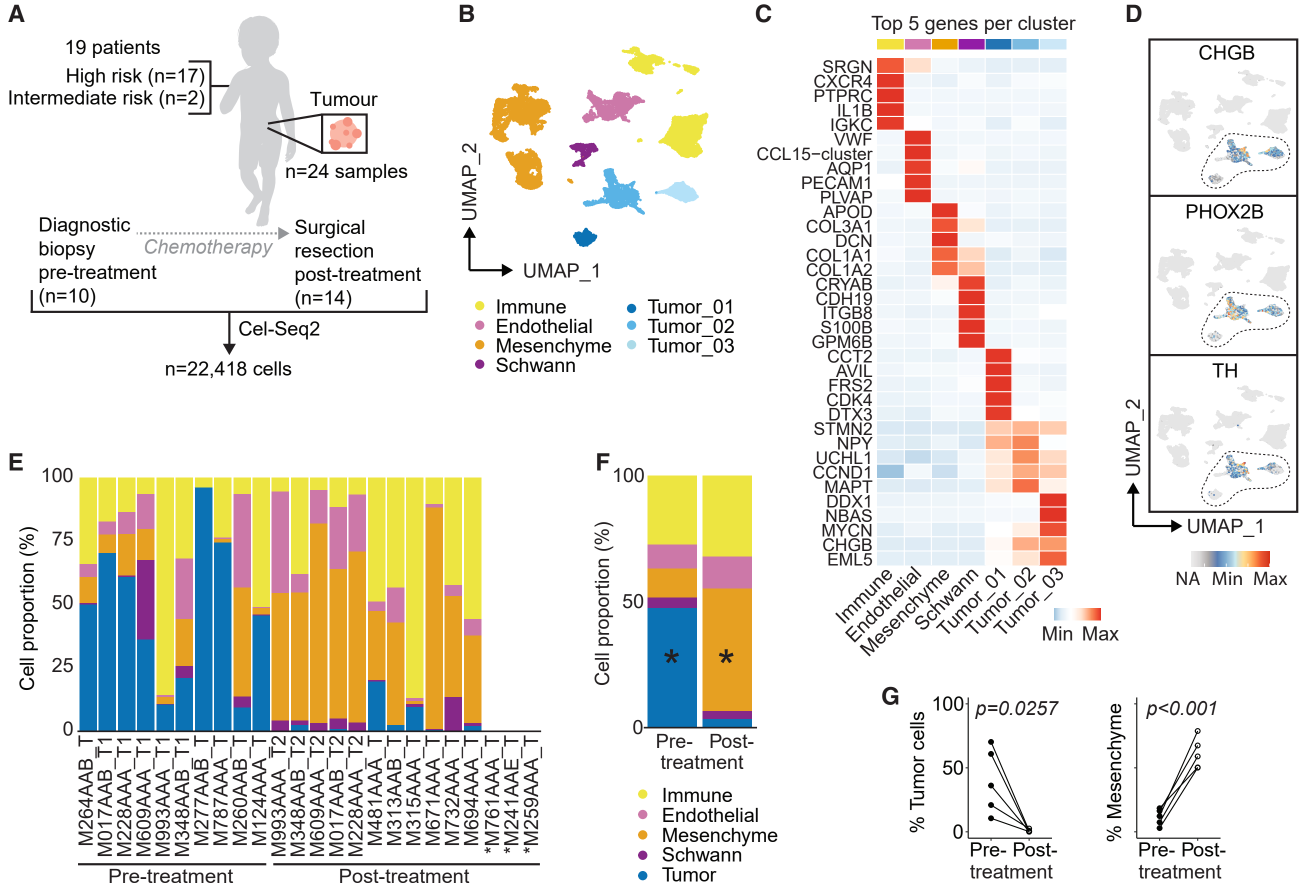


**Figure 1. The single-cell landscape of neuroblastoma**

(A) Overview of patients and samples.

(B) UMAP of main cell types.

(C) Heatmap of top 5 differentially expressed genes per main cell type.

(D) UMAP showing expression of neuroblastoma-associated genes.

(E) Proportion of different cell types per sample. *Only cells which were sorted with an unbiased FACS sorting strategy were included, which led to exclusion of M761AAA_T, M241AAE_T, and M259AAA_T. "T1" and "T2" refer to paired samples before and after treatment, respectively.

(F) Average cellular composition of samples before and after induction chemotherapy. *Mixed-effects analysis with Sidak's multiple comparisons test.* *$p < 0.005$.

(G) Proportion of mesenchymal and tumor cells in five paired pre- and post-treatment samples. *Mann-Whitney U-test.* Also see Figure S1.

undifferentiated monocytes (Mo), and four differentiated macrophage populations (*IL10*hi, *CCL2*hi, *APO*hi, and *MAF*hi macrophages [Mφ; Figure 2B]). We confirmed their presence by flow cytometry and established their similarity with previously identified populations in neuroblastoma (Figures S2C–S2E).[23,24]

cDC, and *IL10*hi, *APO*hi and *MAF*hi Mφ had the highest expression of HLA and costimulatory molecules ($p < 0.0001$ versus *S100*hi Mo), rendering them the most potent interaction partners for T cell co-stimulation (Figures 2C and S2F). Compared to the undifferentiated *S100*hi Mo, all macrophage populations displayed an M2-like signature, associated with immunosuppressive and pro-tumorigenic properties ($p < 0.0001$; Figure 2D),[28,29] which was confirmed by protein expression of M2-associated marker CD163 (Figure 2E). The *IL10*hi and *CCL2*hi Mφ populations had a mixed profile, expressing M1-like (pro-inflammatory) features next to M2-like characteristics ($p < 0.0001$; Figure 2D). The immunoregulatory nature of the myeloid populations was further substantiated by their expression of soluble factors with acknowledged immunosuppressive roles in tumors, such as *IL10*, *LGALS3*, and *MMP9* (Figure S2G).[30–32] These results indicate, and confirm previous reports,[23,24] that macrophages in neuroblastoma have immunosuppressive features which may tune lymphoid responses.

## Lymphocyte subsets in neuroblastoma display features of dysfunctionality

The lymphoid compartment consisted of NK cells, T cells, and (plasma) B cells (Figure 3A). NK cells expressed genes encoding cytotoxic molecules (*GNLY*, *GZMA*, *GZMB*, and *PRF1*) and γδT cells expressed their T cell receptors (TCR; *TRDC*, *TRGC1/2*). Among αβT cells, we identified CD8$^+$, CD4$^+$, CD4$^+$FOXP3$^+$ regulatory T cells (Tregs), and naive(-like) T cells. These populations were largely equivalent to previously identified clusters in neuroblastoma (Figure S3A).[23,24] Histological assessment revealed that T cells were present in tumor-rich areas (Figure S3B). CD8$^+$ T cells expressed *CD8A*, cytotoxic effector molecules

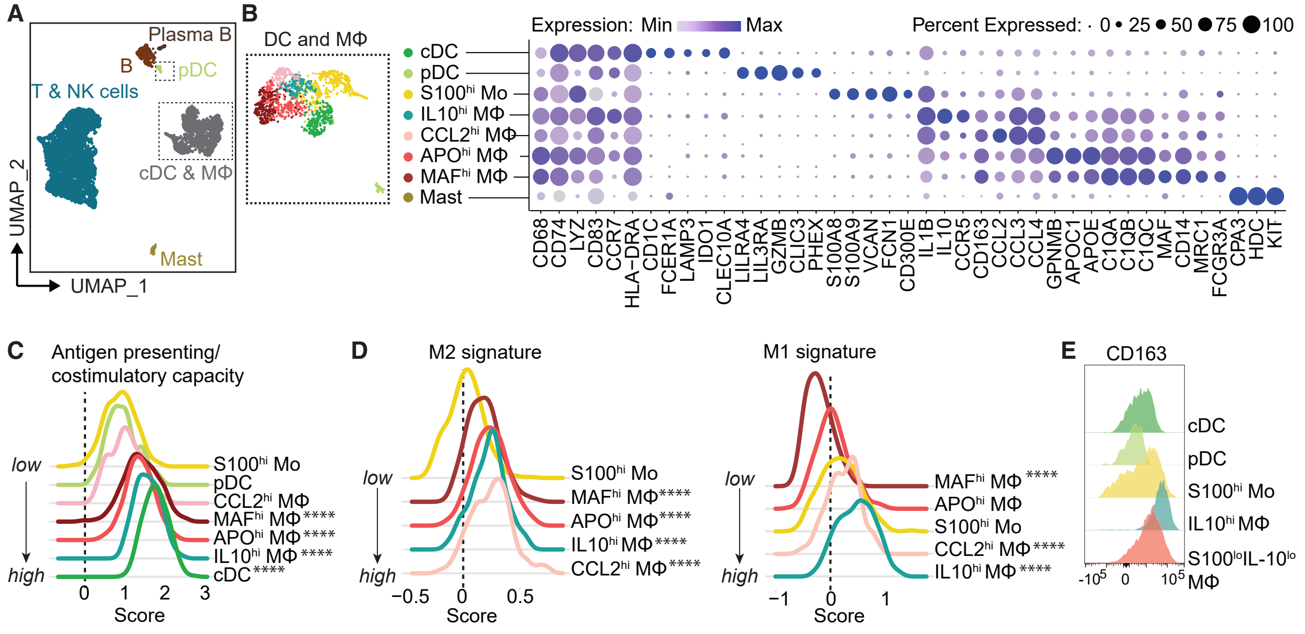


**Figure 2. Neuroblastoma is populated by immunosuppressive macrophages**

(A) UMAP of immune cells.

(B) UMAP and dotplot of myeloid compartment with conventional dendritic cell (cDC), plasmacytoid dendritic cell (pDC), undifferentiated monocyte (Mo), and differentiated macrophage (Mφ) populations showing a selection of their marker genes.

(C) Antigen presenting/co-stimulatory capacity score as constructed with genes in Figure S2F.

(D) M1-like and M2-like macrophage signature score in monocytes and macrophages.[28] ****p < *0.0001* versus *S100$^{hi}$ Mo, Kruskal Wallis with Dunn's*.

(E) Flow cytometry of CD163 in myeloid populations. Also see Figure S2.

(*PRF1*, *GZMA*, and *GZMB*), and significantly increased levels of *PDCD1* (encoding PD-1) and *LAG3* compared to other T/NK cell clusters (Figures 3A and 3B). Since PD-1 and LAG-3 are typically regarded as markers of T cell dysfunction/exhaustion, their expression indicated the presence of a dysfunctional cell fraction among CD8$^+$ cells.[33,34] Tregs expressed high levels of their signature genes *FOXP3*, *CTLA4*, *TIGIT*, and *IL2RA*, in addition to transcription factors *IKZF2*, *BATF*, *PRDM1*, and *MAF* and checkpoint receptors *TNFRSF1B* (TNFR2), *TNFRSF4* (OX-40), and *TNFRSF18* (GITR) (Figures 3A, 3C, and S3C). Expression of these transcription factors and checkpoint receptors is suggestive of an activated and effector Treg profile, which is typically identified in tumor-infiltrating Tregs and associated with enhanced suppressive capacity.[35–39]

Among non-Treg CD4$^+$ T cells we identified two distinct subsets with functional differences. One subset highly expressed *DUSP4*, a signal repressor preventing T cells from over-activation, which has been related to premature T cell aging causing senescence/exhaustion (Figure 3D).[40] DUSP4$^{hi}$ CD4 indeed had a mixed profile of activation and regulation, with on the one hand TCR signaling and expression of proliferation marker *MKI67,* and on the other hand expression of co-inhibitory receptors *PDCD1*, *TIGIT*, *CTLA4* and high PD-1 signaling (Figures 3D and S3D–S3F). Expression of these co-inhibitory receptors by non-Treg T cells is associated with T cell dysfunction.[34,41] Indeed, the *DUSP4*$^{hi}$ CD4$^+$ cluster had significantly increased expression of a published signature for dysfunctional tumor-infiltrating T cells (Figure 3E).[33] In contrast, *TPT1*$^{hi}$ CD4$^+$ T cells had higher translational activity and expressed receptors *IL7R* and *CCR7,* cytotoxic markers (*KLRB1, GNLY*), as well as *CCR6* and *RORA,* associated with Th17 polarization (Figures 3D and S3F). We confirmed the presence of these distinct CD4$^+$ T cells in neuroblastoma by flow cytometry (Figure 3F), including high expression of co-inhibitory receptors and Ki-67 in the *DUSP4*$^{hi}$ cluster (Figures S3G and S3H). These results suggest that *DUSP4*$^{hi}$ CD4 contained a higher proportion of tumor-reactive cells, whereas *TPT1*$^{hi}$ CD4 likely consisted of tissue-resident bystander cells (Figure S3I). This premise was further supported by gene set enrichment analysis (GSEA) utilizing published gene signatures specific for either tumor-infiltrating T cells or blood/normal tissue-associated T cells.[42] GSEA showed that *DUSP4*$^{hi}$ CD4 corresponded largely with previously identified tumor-infiltrating T cell clusters—including those with exhausted profiles—whereas *TPT1*$^{hi}$ CD4 corresponded more with blood/normal tissue-associated Th17 cell clusters (Figure 3G). Bystander *TPT1*$^{hi}$ CD4 may have been attracted to tumors by CCR6 ligand CCL20, which was highly expressed by *IL10*$^{hi}$ Mφ (Figure S2F). Taken together, the neuroblastoma immune environment is characterized by lymphocyte subsets with various features of dysfunctionality, and highly immunosuppressive effector Tregs.

## NK cells in pre-treatment neuroblastoma tumors are dysfunctional

Nearly all annotated immune subsets were detected in each individual tumor sample, albeit in varying proportions (Figures S4A and S4B). To assess the effect of induction chemotherapy on tumor immunity we compared the immune composition before (at diagnosis) and after induction chemotherapy (at surgical resection) (Figure 4A). While the total proportion of immune cells was

CellPress
OPEN ACCESS

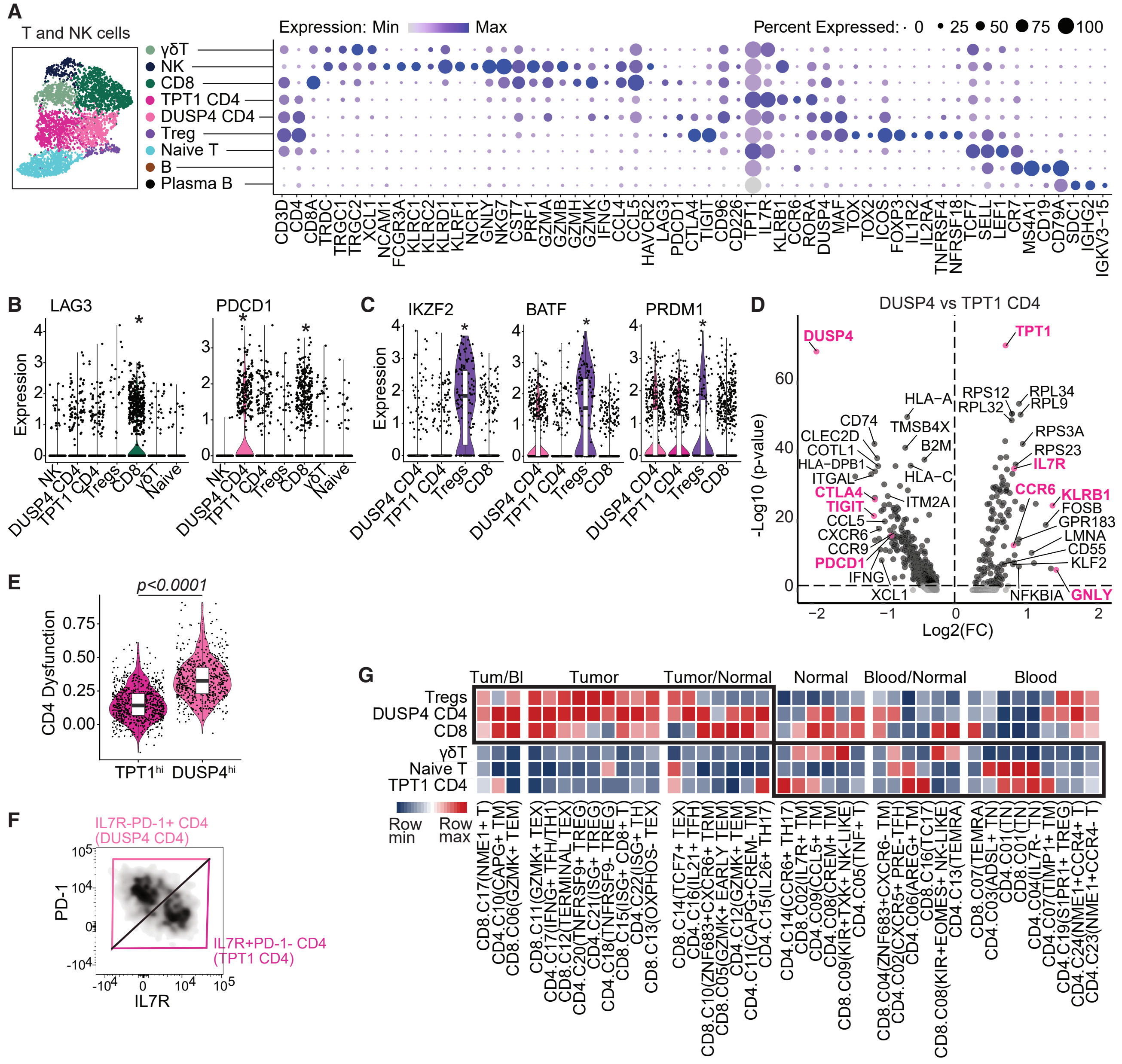


**Figure 3. Neuroblastomas are characterized by lymphoid populations with differing degrees of dysfunctionality**

(A) UMAP and dotplot of T and natural killer (NK) cell subclusters with a selection of their marker genes.

(B) Expression of *LAG3* and *PDCD1* in T/NK cell clusters. **padj < 0.0001 in FindAllMarkers analysis among lymphocytes*.

(C) Transcription factors associated with effector Treg profile. **padj < 0.0001 in FindAllMarkers analysis among lymphocytes*.

(D) Volcano plot of differentially expressed genes between the two CD4 T cell populations.

(E) Expression of a previously published signature for CD4 T cell dysfunction in melanoma. Mann-Whitney U test.[33]

(F) Flow cytometric identification of two CD4 T cell populations by expression of PD-1 and IL-7R.

(G) GSEA comparing neuroblastoma immune cell clusters (in rows) to previously published gene signatures which identify T cell clusters isolated from either blood, healthy tissues (“normal”) or tumors (in columns).[42] The color scale indicates for each neuroblastoma cluster the degree of similarity (NES) with published signatures. Also see Figure S3.

constant (Figure 1F), we observed a trend of a decreased lymphoid/myeloid ratio upon treatment, suggesting a reduction of lymphoid or an influx/expansion of myeloid cells ($p = 0.13$; Figures 4B and S4A–S4C). The strongly M2-differentiated *CCL2*$^{hi}$ Mφ significantly increased after treatment, while B cells decreased (both $p < 0.05$; Figures 4C, 4D, S4D, and S4E). We also observed a trend of NK cell reduction after treatment ($p < 0.1$; Figures 4D and S4E).

Since NK cells are considered essential cytotoxic effectors in neuroblastoma,[43] we assessed whether their functionality changed upon treatment, and compared them to reference NK cells from healthy donor blood (Figures S4F and S4G). Since NK

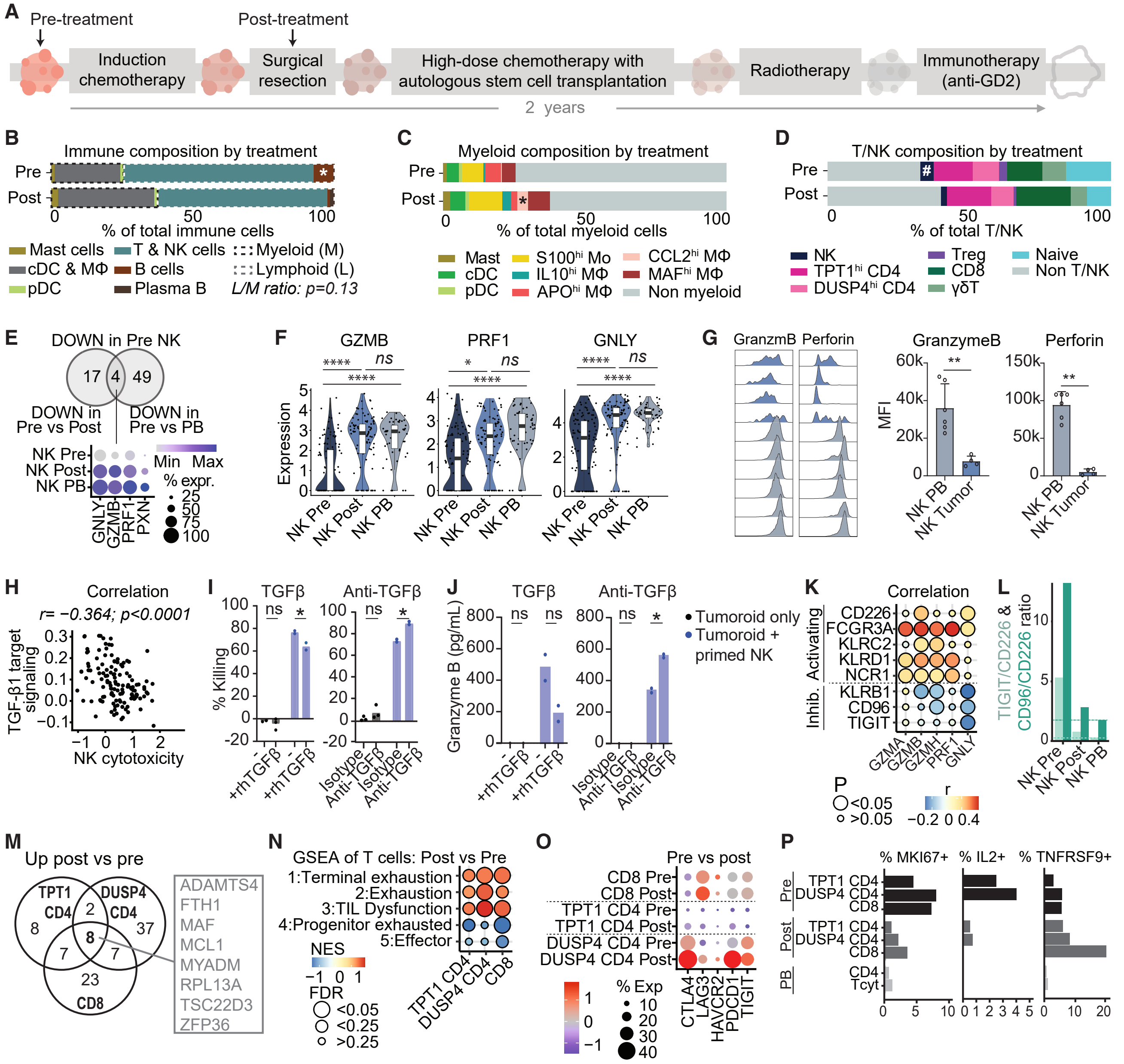


**Figure 4. The immune cell composition and functional profile before and after induction chemotherapy**

(A) Schematic illustration of the high-risk neuroblastoma treatment plan. Arrows indicate sampling timepoints for single-cell RNA sequencing.

(B–D) Average immune (B), myeloid (C), and lymphoid (D) cell composition before and after induction chemotherapy. #*0.05 < p < 0.1 pre* versus *post;* *p < *0.05 pre* versus *post*; *Mann-Whitney U test*.

(E) Downregulated genes in NK cells in pre-treatment tumors compared to either NK cells post-treatment or reference peripheral blood (PB) NK cells (padj < 0.05).

(F) Expression of cytotoxic genes by NK cells in tumors and peripheral blood (PB). *****padj < 0.0001, *padj < 0.05*.

(G) Flow cytometric analysis of granzyme B and perforin expression in neuroblastoma-infiltrating NK cells compared to reference blood NK cells (PB). **p < *0.01; Mann-Whitney U-test, mean ± SD*.

(H) Pearson correlation of TGF-β1 downstream signaling[45] and tumor-infiltrating NK cell cytotoxicity (*modulescore* of *GZMA*, *GZMB*, *PRF1*, *GNLY*, *NKG7*, *CST7*, *CCL5*, and *IFNG*).

(I and J) 24-h killing assay of luciferase-transduced neuroblastoma tumoroid AMC691B by IL-2/IL-15 primed healthy donor blood-derived NK cells with or without rhTGFβ or anti-TGFβ antibody. *Two-way ANOVA,* *p < *0.05.* (I) %Killing = 100-normalized luciferase signal (normalized to tumoroid only). (J) Multiplex immunoassay on supernatant.

(K) Pearson correlation of cytotoxic gene expression with expression of activating and inhibitory receptors in tumor-infiltrating NK cells.

(L) *TIGIT/CD226* and *CD96/CD226* gene expression ratios in NK cells from pre-/post-treatment tumors and from reference blood (PB). Dashed lines indicate ratios in NK PB.

*(legend continued on next page)*

cell function could be affected by micro-environmental effects induced by tumor cells, which were most abundant pre-treatment (Figure 1F), we primarily focused on NK cell function pre-treatment. Compared to NK cells post-treatment or reference NK cells, NK cells in pre-treatment tumors had significantly reduced expression of essential cytotoxic effector genes (*GNLY*, *GZMB*, *PRF1*; Figures 4E, 4F, and S4H). We confirmed low granzyme B and perforin expression, suggesting an impaired cytotoxic function, by flow cytometry (Figure 4G). GSEA further confirmed their reduced cytotoxicity and indicated a dysfunctional immature, resting state in pre-treatment NK cells (Figure S4I), as also observed in other tumor types.[44] Signaling by TGF-β1, a well-known immunosuppressive factor, correlated negatively with NK cell cytotoxicity (Figure 4H).[45] TGF-β downstream signaling was observed in all TME cell types and correlated strongly with TGF-β receptor expression, supporting the presence of active TGF-β in neuroblastoma (Figures S4J and S4K). Intriguingly, NK cells themselves expressed the highest levels of TGF-β1 (Figure S4J). Addition of recombinant TGF-β1 to *in vitro* co-cultures of neuroblastoma tumoroids and primed healthy donor NK cells significantly reduced tumor killing and granzyme B production by NK cells, whereas blockade of TGF-β increased cytotoxicity (Figures 4I and 4J). These observations strongly implicate TGF-β1 signaling in NK cell dysfunction in neuroblastoma.

Since NK cell activity is additionally regulated by a delicate balance of activating and inhibitory receptors, we assessed expression of these receptors and evaluated their correlation with NK cell cytotoxicity (Figure S4L). Overall, activating receptor expression was lower in tumor than blood NK cells (Figure S4M). Expression of activating receptors *CD226* (DNAM-1), *FCGR3A* (CD16), *KLRC2* (NKG2C), *KLRD1* (CD94) and *NCR1* (CD335) positively correlated with cytotoxic genes, while the inhibitory *KLRB1* (CD161), *CD96* and *TIGIT* negatively correlated with cytotoxic genes (Figure 4K). The activating *CD226* and inhibitory *CD96* and *TIGIT* belong to the same checkpoint receptor family competing for ligands. *TIGIT*/*CD226* and *CD96*/*CD226* gene expression ratios were substantially increased in tumor-infiltrating NK cells pre-treatment (Figures 4L and S4N), indicating a disturbed balance shifted toward NK cell inhibition. We confirmed significantly increased CD96 expression, and a trend of increased TIGIT expression in neuroblastoma-infiltrating NK cells by flow cytometry (Figure S4O). These results implicate TGF-β1 and the inhibitory checkpoint receptors KLRB1, TIGIT, and CD96 in NK cell dysfunction in neuroblastoma.

## T cells in post-treatment neuroblastoma tumors are dysfunctional

In contrast to NK cells, T cells did not have reduced cytotoxicity pre-treatment (Figure S5A). Pathway analysis showed that γδ and CD8$^+$ T cells had higher IL-12 signaling pre-treatment than post-treatment (Figures S5B and S5C), suggesting activation. CD8$^+$ and *DUSP4*$^{hi}$ CD4$^+$ T cells additionally had increased interferon and TCR signaling pre-treatment (Figures S5C and S5D), indicating that at least a fraction of those cells may be tumor-reactive, corroborating our findings in Figure 3G. All four effector T cell subsets shared pathways related to TCR signaling in the top 50 of upregulated pathways pre-treatment (Figure S4E). GSEA confirmed increased TCR signaling in all T cell subsets pre-treatment compared to post-treatment (Figure S4F). After treatment however, αβ (CD8$^+$ and CD4$^+$) T cell subsets shared a significantly increased expression of eight genes (Figures 4M, S5G, and S5H), of which transcription factors *MAF* and *TSC33D3*, as well as *MYADM* and *ZFP36* were previously associated with dysfunction of T cells in tumor-microenvironments and/or repression of T cell activation.[33,46–50] GSEA confirmed a significant enrichment of gene signatures for dysfunction in post-treatment compared to pre-treatment αβ T cells, whereas αβ T cells in pre-treatment samples had a higher effector signature (Figure 4N). αβ T cells post-treatment were particularly enriched for signatures associated with "terminal exhaustion" as opposed to "progenitor exhaustion," the latter being associated with some level of retained functionality and tumor control.[51] However, compared to healthy donor blood-derived T cells, both pre- and post-treatment tumor-infiltrating T cells showed a significant enrichment of dysfunction/exhaustion signatures (Figure S5I). The co-inhibitory receptors *LAG3* (LAG-3), *CTLA4* (CTLA-4), *PDCD1* (PD-1) and *HAVCR2* (TIM-3), extensively linked to T cell exhaustion/dysfunction,[33,34] were among the core enriched upregulated genes post-treatment (Figures 4O and S5J). Lower fractions of *MKI67* (Ki-67) and *IL2* (IL-2) expressing cells and increased fractions of *TNFRSF9* (4-1BB) expressing cells confirmed reduced (proliferative) activity and suggested prolonged antigen stimulation post-treatment (Figure 4P).[53,54] Taken together, these results imply that a fraction of T cells is tumor-reactive and becomes more exhausted/dysfunctional after treatment, possibly due to prolonged antigen stimulation. The high expression of co-inhibitory receptors in combination with overall low *TOX*/*TOX2* expression (Figure 3A) however suggests potentially retained responsiveness to immune checkpoint blockade (ICB).

## Immunoregulatory interactions in neuroblastoma

Lymphocyte dysfunction in tumors may result from prolonged antigen stimulation, but also from suppressive microenvironmental cues. To unravel these cues, we analyzed the interactions between T/NK cells and other cells in the tumor using the prediction tool CellChat.[52] The combined population of T/NK cells (defined in Figure 2A) displayed a multitude of interactions with other cells, of which those with myeloid cells were most abundant (Figures 5A, S6A, and S6B). To specifically identify therapeutically targetable immunomodulatory interactions, we focused on interactions between T/NK cells and four immunoregulatory interactions partners, i.e., myeloid cells, tumor cells, mesenchymal cells and Tregs. Interactions with these partners included, among others, interactions involved in cellular adhesion, chemoattraction and immune regulation (Figures 5B and S6A–S6F). Among these we identified a number of cellular interactions with a known immunosuppressive role

(M) Venn diagram of shared upregulated genes (*padj* < 0.05) in CD4 and CD8 T cells post-treatment versus pre-treatment.
(N) GSEA of exhaustion and effector signatures (1 + 4: GSE84105, 2 + 5,[78] 3[33]) in CD4 and CD8 T cells pre-/post-treatment. *NES = normalized enrichment score*.
(O) Dotplot of immune checkpoint receptor genes in CD4 and CD8 T cells.
(P) Fraction of cells expressing proliferation marker *MKI67* (Ki-67), cytokine *IL2* (IL-2) and antigen-stimulated T cell marker *TNFRSF9* (4-1BB). Also see Figures S4 and S5.

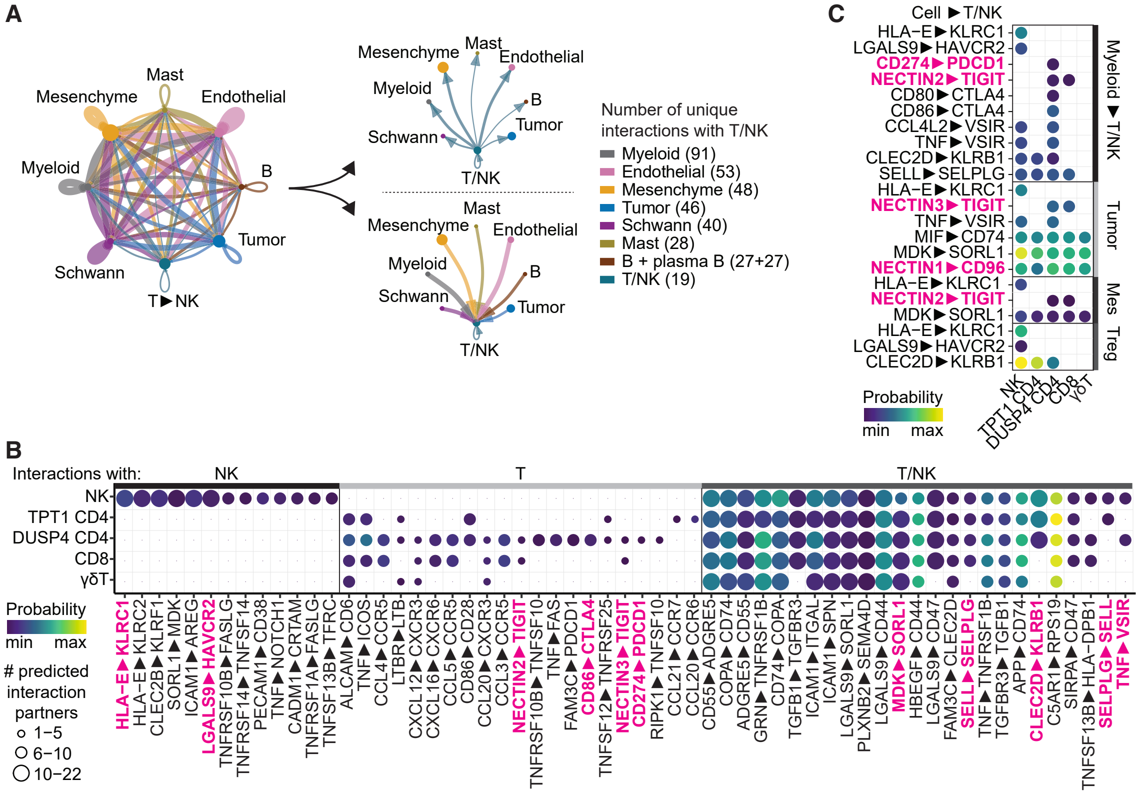


**Figure 5. Immunoregulatory interactions in neuroblastoma**

(A) Interaction network of main cell types in neuroblastoma constructed with CellChat.[52]

(B) Bubbleplot of most frequent predicted interactions between T/NK subsets and all other cells in the TME. Interactions with >5 partners for NK cells, >2 partners for T cells, and >5 partners for T/NK cells (in at least one subset) are shown. The highest probability per interaction per T/NK cluster is indicated. Interactions shown in Figure 5C are highlighted.

(C) Bubbleplot of selected, predicted immunosuppressive interactions with T/NK subsets. Interactions with each specific myeloid subset were evaluated and subsequently merged, with the highest probability of each interaction pair depicted in the plot. Also see Figure S6.

and potential for therapeutic intervention, including *CLEC2D—KLRB1*, *LGALS9—HAVCR2*, *CD274—PDCD1*, *NECTIN2—TIGIT and CD80/CD86—CTLA4* and *NECTIN1—CD96* (Figure 5C). This multitude of immunoregulatory interactions highlights a rationale for ICB combination therapy as opposed to ICB monotherapy.

## Immunosuppressive interactions with potential for therapeutic intervention

ICB has revolutionized cancer therapy and may restore activity of exhausted/dysfunctional T cells.[1] We therefore sought to identify specifically which interactions with tumor-reactive T cells were associated with disturbed T cell function, to therapeutically target exactly those interactions. We assessed the correlation between T cell dysfunction and all predicted interactions (not selected for known immunosuppressive interactions) with an unbiased approach outlined in Figure 6A, using a dysfunction score with established markers (Figures S7A–S7C).[55] We focused our analysis on CD8$^{+}$ and DUSP4 CD4$^{+}$ T cells since these proved to contain most tumor-reactive cells and had the highest dysfunction scores (Figure S7C). The analysis revealed multiple targets which positively correlated with T cell dysfunction (Figure 6B). Among the most frequent targets and those with the highest correlations with T cell dysfunction, we identified *NECTIN2*, a well-known interaction partner of the co-inhibitory receptor TIGIT (Figures 6B, S7D, and S7E). *NECTIN2* on cDC, endothelium, mesenchyme, *IL10*$^{hi}$ Mφ and *APO*$^{hi}$ Mφ was predicted to interact with *TIGIT* on CD8 and *DUSP4*$^{hi}$ CD4 T cells (Figures 6C, S7F, and S7G). We confirmed expression of NECTIN2 by flow cytometry (Figure 6D). Expression of TIGIT was increased in tumor-infiltrating T cells, especially in *DUSP4*$^{hi}$ CD4 and CD8 T cells, compared to reference T cells from blood (Figures 6E and S7H). We confirmed expression of *NECTIN2* and *TIGIT* by these cells in previously published neuroblastoma datasets (Figures S7I and S7J), and confirmed the positive correlation between *NECTIN2* expression and T cell dysfunction in bulk-RNAseq data of 498 neuroblastomas (SEQC cohort GSE49710; Figure 6F).[56] Taken together, the NECTIN2-TIGIT axis may regulate T cell function in neuroblastoma and represents a promising target for therapeutic intervention.

## Combined TIGIT/PD-L1 blockade enhances immune responses against neuroblastoma

To investigate the therapeutic potential of TIGIT blockade in neuroblastoma, we explored its functional role in tumor killing. To consider the possible benefit of ICB combination therapy—based on the abundance of immunoregulatory interactions including

CellPress
OPEN ACCESS

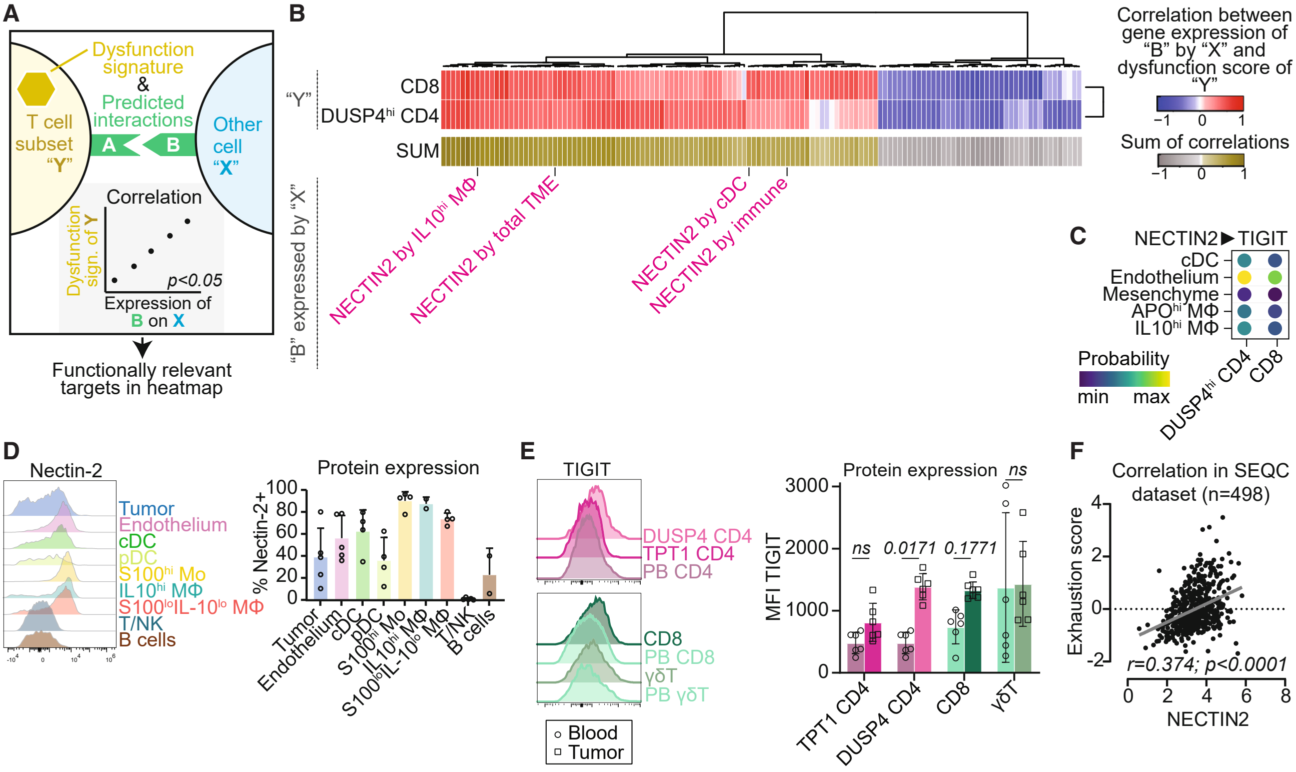


**Figure 6. Targetable immunoregulatory interactions in neuroblastoma**

(A) Graphical representation of analysis strategy for Figure 6B: selection of genes ("B") expressed by population "X" which are involved in a significant ligand-receptor interaction between population "X" and T cell subset "Y", of which the expression by population "X" also significantly correlates with the dysfunction score of T cell subset "Y". Genes with at least one significant correlation, with either *DUSP4*$^{hi}$ CD4 or CD8 T, were included.

(B) Heatmap showing Pearson correlation of expression of genes "B" by populations "X" with dysfunction score of T cell subsets "Y".

(C) All predicted *NECTIN2-TIGIT* interactions with *DUSP4*$^{hi}$ CD4 and CD8 T cells in neuroblastoma.

(D) Flow cytometric validation of nectin-2 protein expression on neuroblastoma tumor samples. *Mean ± SD.*

(E) Flow cytometric validation of TIGIT protein expression on T cell populations infiltrating neuroblastoma, compared to reference T cells from blood (PB). TPT1$^{hi}$ CD4 were gated as IL-7R$^{hi}$PD-1$^{lo}$ CD4$^{+}$ cells and DUSP4$^{hi}$ CD4 were gated as IL-7R$^{lo}$PD-1$^{hi}$ CD4$^{+}$ cells, as shown in Figure 3F. *Two-way ANOVA with Sidak's post-hoc test. Mean ± SD.*

(F) Correlation of *NECTIN2* gene expression with dysfunction score in bulk-RNAseq dataset of SEQC cohort consisting of 498 neuroblastomas (r2.amc.nl; Tumor Neuroblastoma–SEQC–498–RPM–seqcnb1; GSE49710). Also see Figure S7.

*CD274-PDCD1* (Figure 5C) —we also tested combined TIGIT/PD-L1 blockade. PD-L1 was highly expressed by myeloid cells, particularly *IL10*$^{hi}$ Mφ (Figures S8A and S8B). Moreover, neuroblastoma-infiltrating T cells, particularly *DUSP4*$^{+}$ CD4 T cells and CD8 T cells, displayed active downstream TIGIT and PD-1 signaling (Figure S8C). TIGIT blockade *in vitro* resulted in significantly enhanced killing of neuroblastoma tumoroids by immune cells, when combined with PD-L1 blockade (Figures 7A, 7B, and S8D–S8F). The rationale for this ICB combination was further substantiated by upregulation of nectin-2 and PD-L1 by tumor cells during co-culture (Figure 7C). To elucidate which immune cells contributed to the ICB-enhanced tumor-reactivity, we performed killing assays with isolated immune subsets: CD4, CD8, γδT, NK cells, and full peripheral blood mononuclear cells (PBMC) lacking each of these populations (Figure S8G). A trend of increased killing by the ICB combination was conserved in 3/3 donors in cultures with each of the four separate populations and with PBMC lacking CD4 or γδT cells. However, in cultures with PBMC lacking CD8 or NK cells, the enhanced killing efficacy was lost in 2/3 donors. CD8 T cells and NK cells might thus contribute to the ICB-induced response, and cultures/tumors without CD8 T cells and/or NK cells might benefit less from combined anti-TIGIT/PD-L1 therapy.

*In vivo*, combined TIGIT/PD-L1 blockade significantly improved survival and led to sustained CR in 2 out of 6 animals in two immunocompetent syngeneic murine models (N1E-115 and Neuro2a), a partial response in Neuro2a, and 1 transient CR and 1 sustained CR in a third model (N18) (Figures 7D–7G, S8H, and S8I). Moreover, the combination treatment significantly improved survival compared to anti-PD-L1 monotherapy in 2/3 models, suggesting a significant benefit of TIGIT blockade. None of the treatments led to changes in body weight or other toxicities (Figure S8J).

To understand the immunobiology of ICB *in vivo* we performed high-dimensional flow cytometry on the treated tumors. The percentage of CD45$^{+}$ tumor-infiltrating immune cells was not affected by ICB treatment (Figure S8K). CD11b$^{+}$ myeloid cells were highly abundant across models and treatments (Figures 7H and S8L–S8N). Intriguingly, infiltration of CD8$^{+}$ T cells, NK cells and DC correlated with a lower tumor volume in 2 out of 3 models, while infiltration with potentially

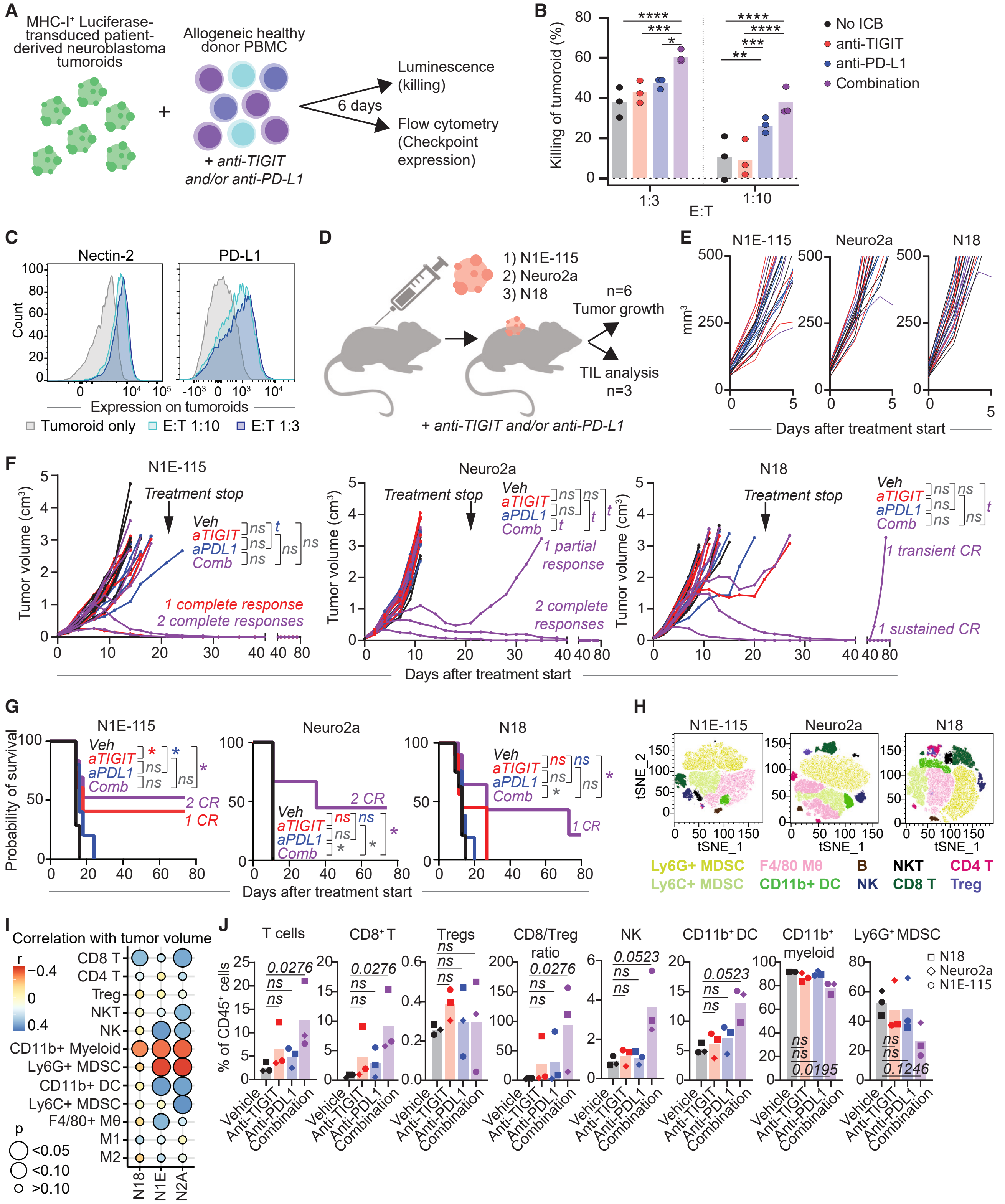


**Figure 7. Combined TIGIT/PD-L1 blockade enhances immune responses against neuroblastoma**

(A) *In vitro* killing assay with luciferase-transduced neuroblastoma tumoroids (AMC691T) and healthy donor PBMC. Tumor cells and PBMC were cocultured for 6 days ± anti-TIGIT and/or anti-PD-L1.

(legend continued on next page)

immunosuppressive $CD11b^+$ myeloid cells and, specifically, $Ly6G^+$ myeloid-derived suppressor cells, correlated with a higher tumor volume (Figure 7I). The combination treatment significantly increased the percentage of T cells, specifically $CD8^+$ T cells, leading to an increased CD8/Treg ratio (Figure 7J). A trend of increased NK cells and DC was also observed. On the other hand, the percentage of total $CD11b^+$ myeloid cells was decreased, with a trend of reduced $Ly6G^+$ MDSC (Figure 7J). Taken together, these results imply that TIGIT+PD-L1 blockade may engage CD8 T cells and NK cells, and may significantly modulate the immune microenvironment of neuroblastoma into a less immunosuppressive milieu. In conclusion, we identified combined TIGIT+PD-L1 blockade as a relevant intervention for neuroblastoma with therapeutic potential *in vivo*.

### TIGIT blockade improves survival in a chemotherapy-resistant neuroblastoma model

Tumor relapses are the major cause of death in patients with neuroblastoma, and more effective treatments are especially urgent for these patients. We therefore investigated the added effect of TIGIT blockade in an immunologically cold, chemotherapy-resistant syngeneic model mirroring relapsed/refractory patients. The model was generated by allograft of neurospheres derived from *Th-ALK*$^{F1174L}$/*MYCN* 129/SvJ transgenic spontaneous tumors subjected to repeat chemotherapy (Figures S9A–S9F).[57] TIGIT ± PD-L1 blockade was added to temozolomide and irinotecan (TEM/IRI) + anti-GD2 treatment, which is favored as the standard backbone treatment for relapsed/refractory patients in Europe (Figures 8A and 8B). The combination of TEM/IRI+anti-GD2 with ICB significantly improved survival compared to the vehicle control (Figure 8C). Remarkably, TEM/IRI+anti-GD2 was already highly efficacious, and addition of ICB did not significantly improve survival compared to this backbone. Addition of TIGIT ± PD-L1 blockade did however result in three sustained (>150 days) CR (Figures 8C and 8D). Notably, addition of anti-TIGIT to anti-PD-L1 significantly reduced tumor growth and effectuated a trend of increased survival, while PD-L1 blockade by itself did not effectuate a clear survival benefit, possibly due to relatively low expression of PD-L1 in the tumor (Figure S9G). This highlights the relevance of TIGIT as immune checkpoint in these tumors. None of the treatments led to toxicities (Figure S9H).

Since the tumors in this study were relatively small (<50 $mm^3$ at start of treatment), we also tested TIGIT blockade in animals with larger tumors (50–200 $mm^3$; Figure S9I). In these animals, TEM/IRI+anti-GD2 treatment alone was less efficacious, and addition of TIGIT blockade significantly improved animal survival compared to the backbone treatment alone ($p = 0.0299$; Figures 8E–8G, S9J, and S9K). To understand the immunobiology of the different responses in small and large tumors, we compared their tumor-microenvironments. Smaller tumors had significantly higher $CD45^+$ immune cell infiltration (Figure 8H), resulting in significantly higher $CD8^+$ and $CD8^-CD4^-$ (possibly $\gamma\delta$) T cell fractions, higher $CD11b^+$ myeloid fractions, including F4/$80^+$ M$\varphi$, and a trend of higher B cells and M2-like M$\varphi$ as fraction of total cells in the tumor (Figures 8I, 8J, and S9L). The higher presence of effector cells might explain why the ICB treatment induced CR in a number of animals with small tumors, but not in large tumors.

Lastly, to investigate whether TIGIT ± PD-L1 blockade could add value in the context of adoptive cell therapies, we analyzed single-cell RNA sequencing data from a recent clinical trial with anti-GD2 CAR-NKT cells in neuroblastoma. This publication included analyses of isolated CAR-NKT cells pre-infusion and post-infusion.[58] *TIGIT* expression significantly increased on CAR-NKT cells post-infusion and was highest in patients not responding to the treatment, while *PDCD1* expression did not differ (Figure 8K). This finding could provide a rationale to combine adoptive cell therapies with TIGIT blockade in neuroblastoma. Taken together, TIGIT blockade may add a survival benefit in the context of chemotherapy resistance in patients with neuroblastoma, when combined with the currently favored relapse treatment protocol.

## DISCUSSION

In this study we have generated a comprehensive single-cell atlas of the neuroblastoma immune environment, revealing the detailed composition and functional profile of immune cells in neuroblastoma. We exposed a vast immunoregulatory network affecting T and NK cell function and identified TGF-$\beta$1, CD161, PD-1, and TIGIT as potential targets for immunotherapy. With *in vitro* and *in vivo* studies, we demonstrated a significant benefit of anti-TIGIT/PD-L1 combination therapy in three models, a significant contribution of anti-TIGIT to this response, and a significant benefit of adding anti-TIGIT to the treatment of large chemotherapy-resistant tumors. These results nominate TIGIT(+PD-L1) blockade as a treatment option for patients with neuroblastoma, even in the context of chemotherapy resistance.

Our analyses revealed that effector lymphocytes in neuroblastoma are dysfunctional, as also observed in other tumors.[8,9,42] NK cells in neuroblastoma had reduced cytotoxicity, particularly pre-chemotherapy. This resting, immature profile of NK cells recapitulates their previously described state of "arrested

(B) Percentage of tumoroid killing (=100-normalized luminescence; normalized against tumoroid only) by healthy donor PBMC. n = *3 donors. Two-way ANOVA with Tukey.**$p < 0.05$; **$p < 0.01$, ***$p < 0.001$, ****$p < 0.0001$.

(C) Flow cytometric analysis of Nectin-2 and PD-L1 expression on tumoroids cultured with PBMC at different E:T ratios. Gating strategy in Figure S8D.

(D) Graphic representation of *in vivo* study. *TIL = tumor-infiltrating leukocytes*.

(E and F) Tumor volumes in N1E-115, Neuro2a and N18 mouse models (n = 6 per group) treated with anti-TIGIT and/or anti-PD-L1 from day 0–5 (E) and up to day 80 of follow-up (F). Treatment was discontinued after 3 weeks. *Linear Mixed-Effects Models. t = trend* ($0.05 < p < 0.1$).

(G) Survival analysis of N1E-115, Neuro2a and N18 models (n = 6 per group) treated with anti-TIGIT and/or anti-PD-L1. *Matched log rank (Mantel-Cox) test.* *$p < 0.05$.

(H–J) Flow cytometric analysis of the TME *in vivo* in n = 3 mice per treatment condition, treated with anti-TIGIT and/or anti-PD-L1 for 7 days (H) TSNE of $CD45^+$ cells in the three models, all treatment conditions combined. (I) Pearson correlations between tumor volumes and fraction of each immune cell population (of $CD45^+$ cells). (J) Fraction of each immune cell population (of $CD45^+$ cells) and CD8/Treg ratio. Data were combined for each treatment condition; the mean value of n = 3 mice from each model is shown. *Kruskal-Wallis with Dunn's*. Also see Figure S8.

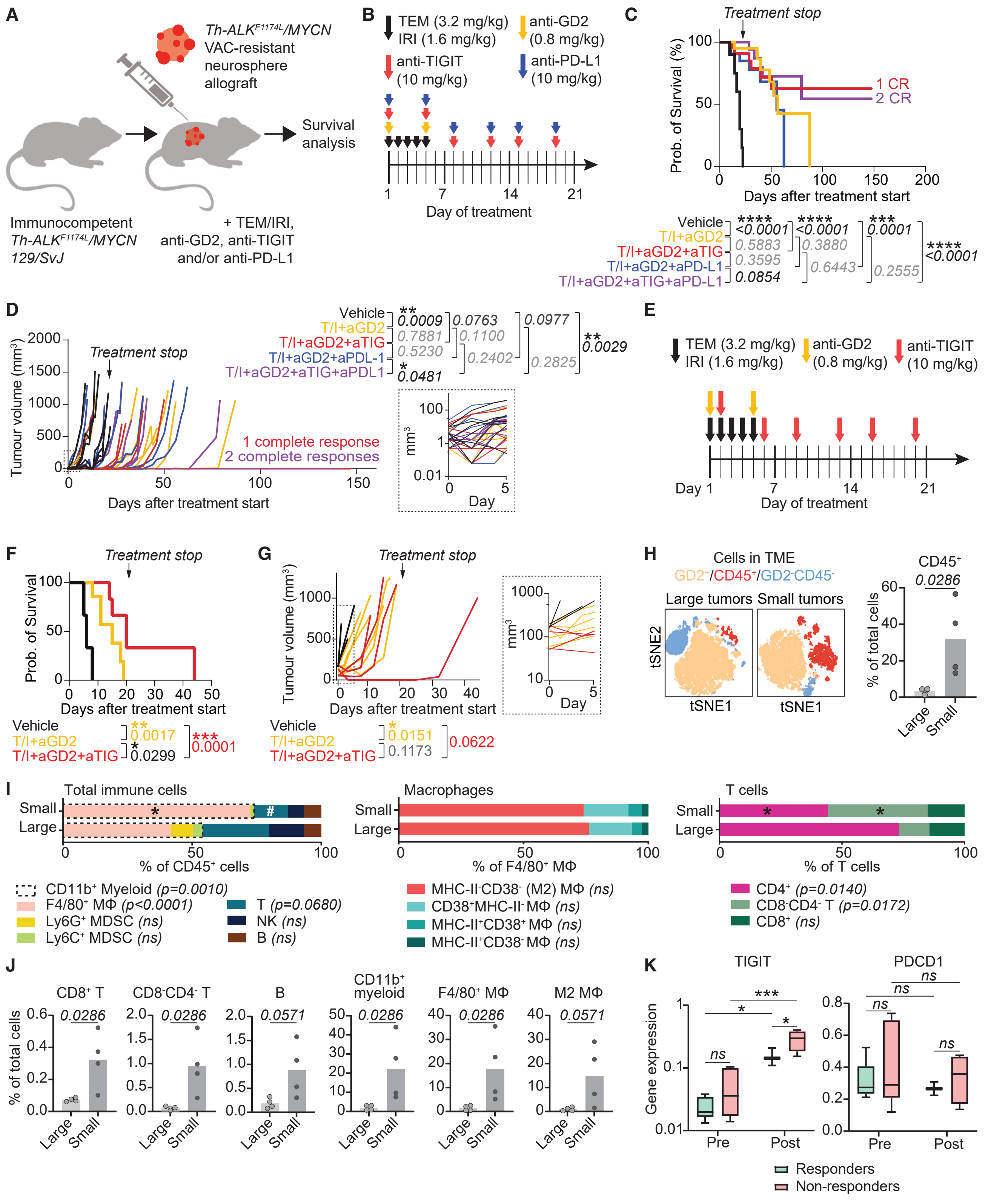


**Figure 8. TIGIT blockade improves survival in a chemotherapy-resistant neuroblastoma model**

(A) Graphic representation of study setup, adding anti-TIGIT and/or PD-L1 to the standard relapse backbone treatment (Temozolomide/Irinotecan (TEM/IRI) + anti-GD2) in a chemotherapy-resistant, immunologically cold model.

(legend continued on next page)

development" in other tumors.[44] NK cell dysfunction in neuroblastoma is not limited to the tumor but has also been observed in peripheral blood of patients, throughout the treatment course.[59] A recently published phase II trial adding anti-GD2, considered to rely at least partly on activity of NK cells,[43] to induction chemotherapy, demonstrated improved early responses and event-free survival in comparison with historic reference cohorts.[60] This added value of anti-GD2 during the induction phase—despite the dysfunctionality of NK cells in pre-treatment tumors—suggests that immunotherapy may be able to reverse or at least ameliorate the arrested state of NK cells,[60] as also previously observed in peripheral blood of patients.[59] Another explanation could be that not NK cells, but rather myeloid populations are the main effector cells mediating anti-GD2 efficacy.[61]

While T cells in neuroblastoma showed signs of tumor reactivity pre-treatment, they exhibited features of dysfunction post-treatment.[8,62] T cell dysfunction may result from chronic stimulation of T cells and/or immunomodulatory signals.[1,63] Considering the high expression of antigen-experienced T cell marker 4-1BB post-treatment, prolonged antigen exposure likely contributed to the development of T cell dysfunction[53,54]; months of induction chemotherapy may have increased tumor cell immunogenicity, eliciting chronic T cell activation with subsequent exhaustion.[64] However, we cannot rule out that chemotherapy may have also directly contributed to induction of dysfunctional features in T cells post-treatment.[65] Yet, the overall low *TOX*/*TOX2* expression suggests potential retained responsiveness to ICB.

We revealed an abundance of immunomodulatory interactions between T/NK cells and other cells in the TME, which included suppressive macrophages and effector-differentiated Tregs. Effector-differentiation of Tregs, observed in various tumors, has been related to enhanced suppressive capacity, which nominates Tregs as targets for immunotherapeutic interventions.[35,36] The identified M2-like-differentiated macrophages have recently moved into the spotlight of immunotherapy: combined anti-GD2/CD47 therapy to activate Mφ resulted in highly promising anti-tumor activity, accompanied by recruitment of M1-like Mφ and reduced M2-like Mφ.[28,29,61] Taken together, the abundance of immunosuppressive cells warrants exploration of immunotherapy combination strategies.

We identified the immunoregulatory *NECTIN2—TIGIT* axis as a target with high potential for therapeutic intervention. *NECTIN2—TIGIT* interaction has been reported in other solid tumors,[66,67] underlining its universality in tumor microenvironments. Blockade of TIGIT may not only increase T cell, but also NK cell antitumor immunity, which is likely partly mediated by decreasing the suppressive capacity of Tregs.[68–71] Moreover, combined TIGIT/PD-L1 blockade enhanced antitumor responses in various solid cancers *in vitro* and *in vivo* and the observed *in vivo* responses shared high similarity with ours, resulting in CR in a part of the animals.[68,69,71] This heterogeneous response to ICB in syngeneic animal models has been previously related to Mφ-driven ICB resistance,[72] which again highlights the potential of therapeutic interventions directed at Mφ to increase responses to ICB. Moreover, it underscores the importance of identifying biomarkers for treatment response to select eligible patients. Since the patient numbers in our study were too small to perform patient subgroup analyses, this would be a valuable additional analysis in future studies. Importantly, combining TIGIT+PD-L1 blockade with anti-GD2 was recently shown to be effective also in other neuroblastoma models, and recent clinical trials combining TIGIT and PD-1/PD-L1 blockade have produced encouraging results in advanced solid tumors in adults.[71,73–75]

Our ultimate effort of translation toward an early phase clinical trial, mimicking relapsed/refractory patients with chemotherapy-resistant tumors *in vivo*, demonstrated a survival benefit of adding TIGIT blockade to the currently favored relapse backbone treatment consisting of TEM/IRI+anti-GD2. This paves the way toward clinical development of TIGIT blockade as an alternative ICB in pediatric solid cancers. Moreover, the vast immunoregulatory network in neuroblastoma which we identified provides a rationale and direction for immunotherapy combination strategies. The number and complexity of the immunosuppressive signaling axes offers a likely explanation for the lacking efficacy of mono-immunotherapies.[19,20] To overcome immunosuppressive signaling in T and NK cells, a combination of immunotherapies targeting multiple immunosuppressive pathways simultaneously may be required. For example, the synergistic effect of TIGIT/PD-L1 blockade may be effectuated by their converging restoration of CD226 signaling.[76] The already described anti-GD2/CD47 combination, and a case report of two refractory

(B) Treatment schedule for mice with small tumors.

(C) Survival analysis in mice with small tumors. T/I = TEM/IRI, aGD2 = anti-GD2, aTIG = anti-TIGIT, aPD-L1 = anti-PD-L1. N = 7 mice per group. *Matched analysis, log rank (Mantel-Cox) test*.

(D) Tumor volume measured over time in mice with small tumors. N = 7 mice per group. *Linear Mixed-Effects Models.* Right panel: detailed view of tumor volumes on days 0–5.

(E) Treatment schedule for mice with large tumors.

(F) Survival analysis in mice with large tumors treated with vehicle (n = 3), TEM/IRI + anti-GD2 (n = 6) or TEM/IRI + anti-GD2 + anti-TIGIT (n = 4). *Matched analysis, log rank (Mantel-Cox) test*.

(G) Tumor volume measured over time in mice with large tumors treated with vehicle (n = 3), TEM/IRI + anti-GD2 (n = 6) or TEM/IRI + anti-GD2 + anti-TIGIT (n = 4). *Linear Mixed-Effects Models.* Right panel: detailed view of tumor volumes on days 0–5. (H–J) Flow cytometric analysis of TME in small and large tumors (vehicle condition). N = 4 animals per condition.

(H-J) High-dimensional flow cytometry comparing immune environment in large and small tumors. (H) TNSE representation of TME and percentage of $CD45^+$ cells. *Mann Whitney U test*.

(I) Detailed immune cell, macrophage, and T cell composition in small and large tumors. $^*p < 0.05$ *between small and large,* $^{\#}0.05 < p < 0.1$ *between small and large. Two-way ANOVA*.

(J) Immune cell populations as percentage of total live cells (vehicle condition). *Mann Whitney U test*.

(K) *TIGIT* and *PDCD1* expression in single-cell RNA sequencing data (extracted from R2 (r2.amc.nl; Metelitsa–124509–Seurat_cp10k–GSE223071), of isolated CAR-NKT cells (infusion products) pre- and post-infusion into neuroblastoma patients, in responding and non-responding patients[58]. *Two-way ANOVA.* $^{***}p < 0.001$, $^*p < 0.05$. *Boxplot represents mean, min, and max.* Also see Figure S9.

neuroblastoma patients treated with anti-GD2/PD-1 combination therapy illustrate the potential of such combinations.[61,77] Importantly, future clinical studies investigating combination strategies will have to balance immunotherapy efficacy with the risk of immunotoxicity, and consider biomarkers to predict treatment response.

In conclusion, we have constructed a comprehensive atlas of the neuroblastoma immune environment and identified functionally relevant targets, including the *NECTIN2-TIGIT* axis, for (combination) immunotherapies.

## STAR★METHODS

Detailed methods are provided in the online version of this paper and include the following:

- KEY RESOURCES TABLE
- RESOURCE AVAILABILITY
  - Lead contact
  - Materials availability
  - Data and code availability
- EXPERIMENTAL MODEL AND STUDY PARTICIPANT DETAILS
  - Human participants
  - Animals
  - Tumoroids
- METHOD DETAILS
  - Isolation of cells from tumor biopsies
  - Isolation of cells from peripheral blood
  - Immunohistochemistry
  - CEL-Seq2 library preparation, sequencing & mapping
  - CEL-Seq2 quality control
  - Single-cell RNA sequencing cluster identification
  - Cluster annotation
  - Differential gene expression analysis
  - Cellular composition analysis
  - Pathway and gene set enrichment analysis
  - Interaction analysis
  - Flow cytometry of neuroblastoma tumors
  - *In vitro* tumor killing assays
  - TIGIT and PD-L1 blockade *in vivo*
- QUANTIFICATION AND STATISTICAL ANALYSIS

## SUPPLEMENTAL INFORMATION

Supplemental information can be found online at https://doi.org/10.1016/j.ccell.2023.12.008.

## ACKNOWLEDGMENTS

We would like to acknowledge our FACS facility, particularly Tomasz Poplonski and Tom O'Toole, and the Multiplex facility of the UMC Utrecht, particularly Edward Knol and Rianne Scholman. This work received funding from the European Union's Horizon 2020 research and innovation program under Marie Skłodowska-Curie grant agreement, No. 956285 (VAGABOND), and grant agreement H2020-iPC-826121, European Research Council (ERC), No. 716079 (Predict), KiKa (Kika404 "Toward TIL therapy in neuroblastoma"), Villa Joep, the Alexander von Humboldt Foundation, and is part of research program Vernieuwingsimpuls Vidi (Combining targeted compounds in neuroblastoma tumors; is two better than one?), project 91716482, and Veni (Release the beast: Boosting CAR-T cell immunotherapy for neuroblastoma), project 09150162010022, which are partly financed by the Dutch Research Council (NWO) and by ZonMW. The first three *in vivo* studies were funded by Hoffman-La Roche. L.C. and E.P. received support from Cancer Research UK (CRUK) (C34648/A18339 and C34648/A28278). E.C. received funding from Children with Cancer UK. *In vivo* work at ICH was funded by Hoffman-La Roche and by Stand up to Cancer/CRUK grant RT6188. JA is further supported by GOSH NIHR BRC. TM and LV (single cell genomics facility of Máxima) are funded by KiKa. AK and Il-L were supported by grants from Institut Curie, Inserm, the Institut National Du Cancer (PEDIAC program, INCA_15670, IJ-L), ERC (ERC-2019-SyG, KILL or DIFFERENTIATE, Olivier Delattre) and the Association Hubert Gouin Enfance et Cancer. We sincerely thank Léonard Didelet, Cécile Thirant and Olivier Delattre for their helpful contributions. S.B. acknowledges funding for this research from the Wellcome Trust (grants 206194 and 223135/Z/21/Z). W.W. is supported by Singapore Immunology Network (SIgN), Agency for Science, Technology and Research (A*STAR); Biomedical Research Council (BMRC) Core Research Fund for use-inspired basic research (UIBR) and IAF-PP project H22J2a0043, and Singapore National Medical Research Council (NMRC) project MOH-001401-00.

## AUTHOR CONTRIBUTIONS

Conceptualization: J.W., L.V., W.K., K.K., M.B., E.P., A.R., R.B., R.K., L.C., J.A., H.C., T.M., M.N., and J.J. Data curation: J.W., L.V., W.K., K.K., M.B., E.P., A.R., S.H., Y.M., M.T., K.L., P.L., B.V., S.M., A.K., G.T., M.P.D., J.K., R.K., N.B., T.M., and M.N. Formal Analysis: J.W., L.V., M.B., S.H., P.L., S.M., A.K., R.R., A.S., W.W., and R.K. Funding acquisition: J.W., F.H., A.H., J.K., I.L., N.B., L.C., J.A., H.C., T.M., M.N., and J.M. Investigation: J.W., L.V., W.K., K.K., M.B., S.M., C.H., E.C., E.P., A.R., F.H., S.H., P.L., A.B., E.Z., F.B., J.S., B.V., S.M., A.K., R.R., A.S., W.W., and R.K. Methodology: J.W., L.V., W.K., K.K., M.B., S.M., C.H., E.C., E.P., A.R., R.B., M.V., S.M., A.K., R.R., A.C., W.W., A.H., S.N., L.C., J.A., H.C., T.M., and J.M. Administration: J.W., W.K., K.K., M.B., J.A., and J.M. Resources: J.W., L.V., A.C., F.H., J.C.G., G.T., M.S., M.W., M.P.D., K.S., S.B., S.N., I.J., R.K., N.B., L.C., J.A., H.C., T.M., M.N., and J.M. Software: J.W., L.V., and T.M. Supervision: J.W., A.R., F.H., J.C.G., K.S., S.B., W.W., A.H., S.N., I.J.L., N.B., L.C., J.A., H.C., T.M., M.M., and J.M. Validation: J.W. Visualization: J.W., L.V., M.B., S.H., A.K., R.K., and T.M. Writing – draft: J.W. Writing – review & editing: J.W., L.V., T.M., and J.M. All authors critically revised the manuscript.

## DECLARATION OF INTERESTS

H.C., A.R., and R.B. are employed at Hoffman-La Roche. R.B. also is an employee of Genentech, a member of the Roche Group. J.A. has founder shares in Autolus Ltd. J.A. and L.C. have received research funding from Roche for *in vivo* work. J.M. has received research funding from Roche for *in vitro* work. J.C.G is a member of a DMC for trials sponsored by YmAbs Therapeutics and University of Birmingham, and has had consulting roles for EUSA Pharma, YmAbs Therapeutics, Celgene, Servier and Norgine.

Received: February 15, 2023
Revised: November 10, 2023
Accepted: December 11, 2023
Published: January 4, 2024

## REFERENCES

1. Korman, A.J., Garrett-Thomson, S.C., and Lonberg, N. (2021). The foundations of immune checkpoint blockade and the ipilimumab approval decennial. Nat. Rev. Drug Discov. *21*, 509–528.
2. Yu, A.L., Gilman, A.L., Ozkaynak, M.F., London, W.B., Kreissman, S.G., Chen, H.X., Smith, M., Anderson, B., Villablanca, J.G., Matthay, K.K., et al. (2010). Anti-GD2 antibody with GM-CSF, interleukin-2, and isotretinoin for neuroblastoma. N. Engl. J. Med. *363*, 1324–1334.
3. Ladenstein, R., Pötschger, U., Valteau-Couanet, D., Luksch, R., Castel, V., Ash, S., Laureys, G., Brock, P., Michon, J.M., Owens, C., et al. (2020). Investigation of the Role of Dinutuximab Beta-Based

Immunotherapy in the SIOPEN High-Risk Neuroblastoma 1 Trial (HR-NBL1). Cancers *12*, 309.

4. Smith, M.A., Seibel, N.L., Altekruse, S.F., Ries, L.A.G., Melbert, D.L., O'Leary, M., Smith, F.O., and Reaman, G.H. (2010). Outcomes for children and adolescents with cancer: challenges for the twenty-first century. J. Clin. Oncol. *28*, 2625–2634.
5. Irwin, M.S., Naranjo, A., Zhang, F.F., Cohn, S.L., London, W.B., Gastier-Foster, J.M., Ramirez, N.C., Pfau, R., Reshmi, S., Wagner, E., et al. (2021). Revised Neuroblastoma Risk Classification System: A Report From the Children's Oncology Group. J. Clin. Oncol. *39*, 3229–3241.
6. Mody, R., Naranjo, A., Van Ryn, C., Yu, A.L., London, W.B., Shulkin, B.L., Parisi, M.T., Servaes, S.-E.-N., Diccianni, M.B., Sondel, P.M., et al. (2017). Irinotecan-temozolomide with temsirolimus or dinutuximab in children with refractory or relapsed neuroblastoma (COG ANBL1221): an open-label, randomised, phase 2 trial. Lancet Oncol. *18*, 946–957.
7. Laverdière, C., Liu, Q., Yasui, Y., Nathan, P.C., Gurney, J.G., Stovall, M., Diller, L.R., Cheung, N.-K., Wolden, S., Robison, L.L., and Sklar, C.A. (2009). Long-term outcomes in survivors of neuroblastoma: a report from the Childhood Cancer Survivor Study. J. Natl. Cancer Inst. *101*, 1131–1140.
8. Philip, M., and Schietinger, A. (2022). CD8(+) T cell differentiation and dysfunction in cancer. Nat. Rev. Immunol. *22*, 209–223.
9. Cózar, B., Greppi, M., Carpentier, S., Narni-Mancinelli, E., Chiossone, L., and Vivier, E. (2021). Tumor-Infiltrating Natural Killer Cells. Cancer Discov. *11*, 34–44.
10. Hou, A.J., Chen, L.C., and Chen, Y.Y. (2021). Navigating CAR-T cells through the solid-tumour microenvironment. Nat. Rev. Drug Discov. *20*, 531–550.
11. Del Bufalo, F., De Angelis, B., Caruana, I., Del Baldo, G., De Ioris, M.A., Serra, A., Mastronuzzi, A., Cefalo, M.G., Pagliara, D., Amicucci, M., et al. (2023). GD2-CART01 for Relapsed or Refractory High-Risk Neuroblastoma. N. Engl. J. Med. *388*, 1284–1295.
12. Park, J.R., Digiusto, D.L., Slovak, M., Wright, C., Naranjo, A., Wagner, J., Meechoovet, H.B., Bautista, C., Chang, W.-C., Ostberg, J.R., and Jensen, M.C. (2007). Adoptive transfer of chimeric antigen receptor re-directed cytolytic T lymphocyte clones in patients with neuroblastoma. Mol. Ther. *15*, 825–833.
13. Louis, C.U., Savoldo, B., Dotti, G., Pule, M., Yvon, E., Myers, G.D., Rossig, C., Russell, H.V., Diouf, O., Liu, E., et al. (2011). Antitumor activity and long-term fate of chimeric antigen receptor-positive T cells in patients with neuroblastoma. Blood *118*, 6050–6056.
14. Pule, M.A., Savoldo, B., Myers, G.D., Rossig, C., Russell, H.V., Dotti, G., Huls, M.H., Liu, E., Gee, A.P., Mei, Z., et al. (2008). Virus-specific T cells engineered to coexpress tumor-specific receptors: persistence and antitumor activity in individuals with neuroblastoma. Nat. Med. *14*, 1264–1270.
15. Heczey, A., Louis, C.U., Savoldo, B., Dakhova, O., Durett, A., Grilley, B., Liu, H., Wu, M.F., Mei, Z., Gee, A., et al. (2017). CAR T Cells Administered in Combination with Lymphodepletion and PD-1 Inhibition to Patients with Neuroblastoma. Mol. Ther. *25*, 2214–2224.
16. Yang, L., Ma, X., Liu, Y.-C., Zhao, W., Yu, L., Qin, M., Zhu, G., Wang, K., Shi, X., Zhang, Z., et al. (2017). Chimeric Antigen Receptor 4SCAR-GD2-Modified T Cells Targeting High-Risk and Recurrent Neuroblastoma: A Phase II Multi-Center Trial in China. Blood *130*, 3335.
17. Straathof, K., Flutter, B., Wallace, R., Jain, N., Loka, T., Depani, S., Wright, G., Thomas, S., Cheung, G.W.-K., Gileadi, T., et al. (2020). Antitumor activity without on-target off-tumor toxicity of GD2-chimeric antigen receptor T cells in patients with neuroblastoma. Sci. Transl. Med. *12*, eabd6169.
18. Pasqualini, C., Rubino, J., Brard, C., Cassard, L., André, N., Rondof, W., Scoazec, J.-Y., Marchais, A., Nebchi, S., Boselli, L., et al. (2021). Phase II and biomarker study of programmed cell death protein 1 inhibitor nivolumab and metronomic cyclophosphamide in paediatric relapsed/refractory solid tumours: Arm G of AcSé-ESMART, a trial of the European Innovative Therapies for Children With Canc. Eur. J. Cancer *150*, 53–62.
19. Geoerger, B., Kang, H.J., Yalon-Oren, M., Marshall, L.V., Vezina, C., Pappo, A., Laetsch, T.W., Petrilli, A.S., Ebinger, M., Toporski, J., et al. (2020). Pembrolizumab in paediatric patients with advanced melanoma or a PD-L1-positive, advanced, relapsed, or refractory solid tumour or lymphoma (KEYNOTE-051): interim analysis of an open-label, single-arm, phase 1-2 trial. Lancet Oncol. *21*, 121–133.
20. Davis, K.L., Fox, E., Merchant, M.S., Reid, J.M., Kudgus, R.A., Liu, X., Minard, C.G., Voss, S., Berg, S.L., Weigel, B.J., and Mackall, C.L. (2020). Nivolumab in children and young adults with relapsed or refractory solid tumours or lymphoma (ADVL1412): a multicentre, open-label, single-arm, phase 1-2 trial. Lancet Oncol. *21*, 541–550.
21. Huang, A.C., and Zappasodi, R. (2022). A decade of checkpoint blockade immunotherapy in melanoma: understanding the molecular basis for immune sensitivity and resistance. Nat. Immunol. *23*, 660–670.
22. Wienke, J., Dierselhuis, M.P., Tytgat, G.A.M., Künkele, A., Nierkens, S., and Molenaar, J.J. (2021). The immune landscape of neuroblastoma: Challenges and opportunities for novel therapeutic strategies in pediatric oncology. Eur. J. Cancer *144*, 123–150.
23. Verhoeven, B.M., Mei, S., Olsen, T.K., Gustafsson, K., Valind, A., Lindström, A., Gisselsson, D., Fard, S.S., Hagerling, C., Kharchenko, P.V., et al. (2022). The immune cell atlas of human neuroblastoma. Cell Rep. Med. *3*, 100657.
24. Costa, A., Thirant, C., Kramdi, A., Pierre-Eugène, C., Louis-Brennetot, C., Blanchard, O., Surdez, D., Gruel, N., Lapouble, E., Pierron, G., et al. (2022). Single-cell transcriptomics reveals shared immunosuppressive landscapes of mouse and human neuroblastoma. J. Immunother. Cancer *10*, e004807.
25. Cheung, N.-K.V., and Dyer, M.A. (2013). Neuroblastoma: developmental biology, cancer genomics and immunotherapy. Nat. Rev. Cancer *13*, 397–411.
26. Wölfl, M., Jungbluth, A.A., Garrido, F., Cabrera, T., Meyen-Southard, S., Spitz, R., Ernestus, K., and Berthold, F. (2005). Expression of MHC class I, MHC class II, and cancer germline antigens in neuroblastoma. Cancer Immunol. Immunother. *54*, 400–406.
27. Duraiswamy, J., Turrini, R., Minasyan, A., Barras, D., Crespo, I., Grimm, A.J., Casado, J., Genolet, R., Benedetti, F., Wicky, A., et al. (2021). Myeloid antigen-presenting cell niches sustain antitumor T cells and license PD-1 blockade via CD28 costimulation. Cancer Cell *39*, 1623–1642.e20.
28. Cheng, S., Li, Z., Gao, R., Xing, B., Gao, Y., Yang, Y., Qin, S., Zhang, L., Ouyang, H., Du, P., et al. (2021). A pan-cancer single-cell transcriptional atlas of tumor infiltrating myeloid cells. Cell *184*, 792–809.e23.
29. Biswas, S.K., and Mantovani, A. (2010). Macrophage plasticity and interaction with lymphocyte subsets: cancer as a paradigm. Nat. Immunol. *11*, 889–896.
30. Lamichhane, P., Karyampudi, L., Shreeder, B., Krempski, J., Bahr, D., Daum, J., Kalli, K.R., Goode, E.L., Block, M.S., Cannon, M.J., and Knutson, K.L. (2017). IL10 Release upon PD-1 Blockade Sustains Immunosuppression in Ovarian Cancer. Cancer Res. *77*, 6667–6678.
31. Gilson, R.C., Gunasinghe, S.D., Johannes, L., and Gaus, K. (2019). Galectin-3 modulation of T-cell activation: mechanisms of membrane remodelling. Prog. Lipid Res. *76*, 101010.
32. Juric, V., O'Sullivan, C., Stefanutti, E., Kovalenko, M., Greenstein, A., Barry-Hamilton, V., Mikaelian, I., Degenhardt, J., Yue, P., Smith, V., and Mikels-Vigdal, A. (2018). MMP-9 inhibition promotes anti-tumor immunity through disruption of biochemical and physical barriers to T-cell trafficking to tumors. PLoS One *13*, e0207255.
33. Li, H., van der Leun, A.M., Yofe, I., Lubling, Y., Gelbard-Solodkin, D., van Akkooi, A.C.J., van den Braber, M., Rozeman, E.A., Haanen, J.B.A.G., Blank, C.U., et al. (2019). Dysfunctional CD8 T Cells Form a Proliferative, Dynamically Regulated Compartment within Human Melanoma. Cell *176*, 775–789.e18.
34. Wherry, E.J., Ha, S.-J., Kaech, S.M., Haining, W.N., Sarkar, S., Kalia, V., Subramaniam, S., Blattman, J.N., Barber, D.L., and Ahmed, R. (2007).

Molecular signature of CD8+ T cell exhaustion during chronic viral infection. Immunity *27*, 670–684.

35. Cretney, E., Xin, A., Shi, W., Minnich, M., Masson, F., Miasari, M., Belz, G.T., Smyth, G.K., Busslinger, M., Nutt, S.L., and Kallies, A. (2011). The transcription factors Blimp-1 and IRF4 jointly control the differentiation and function of effector regulatory T cells. Nat. Immunol. *12*, 304–311.

36. De Simone, M., Arrigoni, A., Rossetti, G., Gruarin, P., Ranzani, V., Politano, C., Bonnal, R.J.P., Provasi, E., Sarnicola, M.L., Panzeri, I., et al. (2016). Transcriptional Landscape of Human Tissue Lymphocytes Unveils Uniqueness of Tumor-Infiltrating T Regulatory Cells. Immunity *45*, 1135–1147.

37. Levine, A.G., Arvey, A., Jin, W., and Rudensky, A.Y. (2014). Continuous requirement for the TCR in regulatory T cell function. Nat. Immunol. *15*, 1070–1078.

38. Haskett, S., Ding, J., Zhang, W., Thai, A., Cullen, P., Xu, S., Petersen, B., Kuznetsov, G., Jandreski, L., Hamann, S., et al. (2016). Identification of Novel CD4+ T Cell Subsets in the Target Tissue of Sjögren's Syndrome and Their Differential Regulation by the Lymphotoxin/LIGHT Signaling Axis. J. Immunol. *197*, 3806–3819.

39. Dias, S., D'Amico, A., Cretney, E., Liao, Y., Tellier, J., Bruggeman, C., Almeida, F.F., Leahy, J., Belz, G.T., Smyth, G.K., et al. (2017). Effector Regulatory T Cell Differentiation and Immune Homeostasis Depend on the Transcription Factor Myb. Immunity *46*, 78–91.

40. Bignon, A., Régent, A., Klipfel, L., Desnoyer, A., de la Grange, P., Martinez, V., Lortholary, O., Dalloul, A., Mouthon, L., and Balabanian, K. (2015). DUSP4-mediated accelerated T-cell senescence in idiopathic CD4 lymphopenia. Blood *125*, 2507–2518.

41. Seo, H., Chen, J., González-Avalos, E., Samaniego-Castruita, D., Das, A., Wang, Y.H., López-Moyado, I.F., Georges, R.O., Zhang, W., Onodera, A., et al. (2019). TOX and TOX2 transcription factors cooperate with NR4A transcription factors to impose CD8(+) T cell exhaustion. Proc. Natl. Acad. Sci. USA *116*, 12410–12415.

42. Zheng, L., Qin, S., Si, W., Wang, A., Xing, B., Gao, R., Ren, X., Wang, L., Wu, X., Zhang, J., et al. (2021). Pan-cancer single-cell landscape of tumor-infiltrating T cells. Science *374*, abe6474.

43. Tarek, N., Le Luduec, J.-B., Gallagher, M.M., Zheng, J., Venstrom, J.M., Chamberlain, E., Modak, S., Heller, G., Dupont, B., Cheung, N.-K.V., and Hsu, K.C. (2012). Unlicensed NK cells target neuroblastoma following anti-GD2 antibody treatment. J. Clin. Invest. *122*, 3260–3270.

44. Riggan, L., Shah, S., and O'Sullivan, T.E. (2021). Arrested development: suppression of NK cell function in the tumor microenvironment. Clin. Transl. Immunol. *10*, e1238.

45. Karlsson, G., Liu, Y., Larsson, J., Goumans, M.-J., Lee, J.-S., Thorgeirsson, S.S., Ringnér, M., and Karlsson, S. (2005). Gene expression profiling demonstrates that TGF-beta1 signals exclusively through receptor complexes involving Alk5 and identifies targets of TGF-beta signaling. Physiol. Genom. *21*, 396–403.

46. Chihara, N., Madi, A., Kondo, T., Zhang, H., Acharya, N., Singer, M., Nyman, J., Marjanovic, N.D., Kowalczyk, M.S., Wang, C., et al. (2018). Induction and transcriptional regulation of the co-inhibitory gene module in T cells. Nature *558*, 454–459.

47. Wagle, M.V., Vervoort, S.J., Kelly, M.J., Van Der Byl, W., Peters, T.J., Martin, B.P., Martelotto, L.G., Nüssing, S., Ramsbottom, K.M., Torpy, J.R., et al. (2021). Antigen-driven EGR2 expression is required for exhausted CD8(+) T cell stability and maintenance. Nat. Commun. *12*, 2782.

48. Yan, M., Hu, J., Yuan, H., Xu, L., Liao, G., Jiang, Z., Zhu, J., Pang, B., Ping, Y., Zhang, Y., et al. (2021). Dynamic regulatory networks of T cell trajectory dissect transcriptional control of T cell state transition. Mol. Ther. Nucleic Acids *26*, 1115–1129.

49. Petkau, G., Mitchell, T.J., Chakraborty, K., Bell, S.E., D Angeli, V., Matheson, L., Turner, D.J., Saveliev, A., Gizlenci, O., Salerno, F., et al. (2022). The timing of differentiation and potency of CD8 effector function is set by RNA binding proteins. Nat. Commun. *13*, 2274.

50. Cannarile, L., Delfino, D.V., Adorisio, S., Riccardi, C., and Ayroldi, E. (2019). Implicating the Role of GILZ in Glucocorticoid Modulation of T-Cell Activation. Front. Immunol. *10*, 1823.

51. Miller, B.C., Sen, D.R., Al Abosy, R., Bi, K., Virkud, Y.V., LaFleur, M.W., Yates, K.B., Lako, A., Felt, K., Naik, G.S., et al. (2019). Subsets of exhausted CD8(+) T cells differentially mediate tumor control and respond to checkpoint blockade. Nat. Immunol. *20*, 326–336.

52. Jin, S., Guerrero-Juarez, C.F., Zhang, L., Chang, I., Ramos, R., Kuan, C.-H., Myung, P., Plikus, M.V., and Nie, Q. (2021). Inference and analysis of cell-cell communication using CellChat. Nat. Commun. *12*, 1088.

53. Ye, Q., Song, D.-G., Poussin, M., Yamamoto, T., Best, A., Li, C., Coukos, G., and Powell, D.J., Jr. (2014). CD137 accurately identifies and enriches for naturally occurring tumor-reactive T cells in tumor. Clin. Cancer Res. *20*, 44–55.

54. Williams, J.B., Horton, B.L., Zheng, Y., Duan, Y., Powell, J.D., and Gajewski, T.F. (2017). The EGR2 targets LAG-3 and 4-1BB describe and regulate dysfunctional antigen-specific CD8+ T cells in the tumor microenvironment. J. Exp. Med. *214*, 381–400.

55. Jerby-Arnon, L., Shah, P., Cuoco, M.S., Rodman, C., Su, M.-J., Melms, J.C., Leeson, R., Kanodia, A., Mei, S., Lin, J.-R., et al. (2018). A Cancer Cell Program Promotes T Cell Exclusion and Resistance to Checkpoint Blockade. Cell *175*, 984–997.e24.

56. SEQC/MAQC-III Consortium (2014). A comprehensive assessment of RNA-seq accuracy, reproducibility and information content by the Sequencing Quality Control Consortium. Nat. Biotechnol. *32*, 903–914.

57. Berry, T., Luther, W., Bhatnagar, N., Jamin, Y., Poon, E., Sanda, T., Pei, D., Sharma, B., Vetharoy, W.R., Hallsworth, A., et al. (2012). The ALK(F1174L) mutation potentiates the oncogenic activity of MYCN in neuroblastoma. Cancer Cell *22*, 117–130.

58. Heczey, A., Xu, X., Courtney, A.N., Tian, G., Barragan, G.A., Guo, L., Amador, C.M., Ghatwai, N., Rathi, P., Wood, M.S., et al. (2023). Anti-GD2 CAR-NKT cells in relapsed or refractory neuroblastoma: updated phase 1 trial interim results. Nat. Med. *29*, 1379–1388.

59. Szanto, C.L., Cornel, A.M., Tamminga, S.M., Delemarre, E.M., de Koning, C.C.H., van den Beemt, D.A.M.H., Dunnebach, E., Tas, M.L., Dierselhuis, M.P., Tytgat, L.G.A.M., et al. (2021). Immune Monitoring during Therapy Reveals Activitory and Regulatory Immune Responses in High-Risk Neuroblastoma. Cancers *13*, 2096.

60. Furman, W.L., McCarville, B., Shulkin, B.L., Davidoff, A., Krasin, M., Hsu, C.-W., Pan, H., Wu, J., Brennan, R., Bishop, M.W., et al. (2022). Improved Outcome in Children With Newly Diagnosed High-Risk Neuroblastoma Treated With Chemoimmunotherapy: Updated Results of a Phase II Study Using hu14.18K322A. J. Clin. Oncol. *40*, 335–344.

61. Theruvath, J., Menard, M., Smith, B.A.H., Linde, M.H., Coles, G.L., Dalton, G.N., Wu, W., Kiru, L., Delaidelli, A., Sotillo, E., et al. (2022). Anti-GD2 synergizes with CD47 blockade to mediate tumor eradication. Nat. Med. *28*, 333–344.

62. Miggelbrink, A.M., Jackson, J.D., Lorrey, S.J., Srinivasan, E.S., Waibl-Polania, J., Wilkinson, D.S., and Fecci, P.E. (2021). CD4 T-Cell Exhaustion: Does It Exist and What Are Its Roles in Cancer? Clin. Cancer Res. *27*, 5742–5752.

63. Zhao, Y., Shao, Q., and Peng, G. (2020). Exhaustion and senescence: two crucial dysfunctional states of T cells in the tumor microenvironment. Cell. Mol. Immunol. *17*, 27–35.

64. Wu, J., and Waxman, D.J. (2018). Immunogenic chemotherapy: Dose and schedule dependence and combination with immunotherapy. Cancer Lett. *419*, 210–221.

65. Gassner, F.J., Zaborsky, N., Neureiter, D., Huemer, M., Melchardt, T., Egle, A., Rebhandl, S., Catakovic, K., Hartmann, T.N., Greil, R., et al. (2014). Chemotherapy-induced augmentation of T cells expressing inhibitory receptors is reversed by treatment with lenalidomide in chronic lymphocytic leukemia. Haematologica *99*, 67–69.

66. Zhang, Q., He, Y., Luo, N., Patel, S.J., Han, Y., Gao, R., Modak, M., Carotta, S., Haslinger, C., Kind, D., et al. (2019). Landscape and

CellPress
OPEN ACCESS

Dynamics of Single Immune Cells in Hepatocellular Carcinoma. Cell *179*, 829–845.e20.

67. Liu, Y., Feng, W., Dai, Y., Bao, M., Yuan, Z., He, M., Qin, Z., Liao, S., He, J., Huang, Q., et al. (2021). Single-Cell Transcriptomics Reveals the Complexity of the Tumor Microenvironment of Treatment-Naive Osteosarcoma. Front. Oncol. *11*, 709210.
68. Johnston, R.J., Comps-Agrar, L., Hackney, J., Yu, X., Huseni, M., Yang, Y., Park, S., Javinal, V., Chiu, H., Irving, B., et al. (2014). The immunoreceptor TIGIT regulates antitumor and antiviral CD8(+) T cell effector function. Cancer Cell *26*, 923–937.
69. Zhang, Q., Bi, J., Zheng, X., Chen, Y., Wang, H., Wu, W., Wang, Z., Wu, Q., Peng, H., Wei, H., et al. (2018). Blockade of the checkpoint receptor TIGIT prevents NK cell exhaustion and elicits potent anti-tumor immunity. Nat. Immunol. *19*, 723–732.
70. Kurtulus, S., Sakuishi, K., Ngiow, S.-F., Joller, N., Tan, D.J., Teng, M.W.L., Smyth, M.J., Kuchroo, V.K., and Anderson, A.C. (2015). TIGIT predominantly regulates the immune response via regulatory T cells. J. Clin. Invest. *125*, 4053–4062.
71. Siebert, N., Zumpe, M., Schwencke, C.H., Biskupski, S., Troschke-Meurer, S., Leopold, J., Zikoridse, A., and Lode, H.N. (2023). Combined Blockade of TIGIT and PD-L1 Enhances Anti-Neuroblastoma Efficacy of GD2-Directed Immunotherapy with Dinutuximab Beta. Cancers *15*, 3317.
72. Aslan, K., Turco, V., Blobner, J., Sonner, J.K., Liuzzi, A.R., Núñez, N.G., De Feo, D., Kickingereder, P., Fischer, M., Green, E., et al. (2020). Heterogeneity of response to immune checkpoint blockade in hypermutated experimental gliomas. Nat. Commun. *11*, 931.
73. Niu, J., Maurice-Dror, C., Lee, D.H., Kim, D.-W., Nagrial, A., Voskoboynik, M., Chung, H.C., Mileham, K., Vaishampayan, U., Rasco, D., et al. (2022). First-in-human phase 1 study of the anti-TIGIT antibody vibostolimab as monotherapy or with pembrolizumab for advanced solid tumors, including non-small-cell lung cancer(☆). Ann. Oncol. *33*, 169–180.
74. Mettu, N.B., Ulahannan, S.V., Bendell, J.C., Garrido-Laguna, I., Strickler, J.H., Moore, K.N., Stagg, R., Kapoun, A.M., Faoro, L., and Sharma, S. (2022). A Phase 1a/b Open-Label, Dose-Escalation Study of Etigilimab Alone or in Combination with Nivolumab in Patients with Locally Advanced or Metastatic Solid Tumors. Clin. Cancer Res. *28*, 882–892.
75. Cho, B.C., Abreu, D.R., Hussein, M., Cobo, M., Patel, A.J., Secen, N., Lee, K.H., Massuti, B., Hiret, S., Yang, J.C.H., et al. (2022). Tiragolumab plus atezolizumab versus placebo plus atezolizumab as a first-line treatment for PD-L1-selected non-small-cell lung cancer (CITYSCAPE): primary and follow-up analyses of a randomised, double-blind, phase 2 study. Lancet Oncol. *23*, 781–792.
76. Banta, K.L., Xu, X., Chitre, A.S., Au-Yeung, A., Takahashi, C., O'Gorman, W.E., Wu, T.D., Mittman, S., Cubas, R., Comps-Agrar, L., et al. (2022). Mechanistic convergence of the TIGIT and PD-1 inhibitory pathways necessitates co-blockade to optimize anti-tumor CD8(+) T cell responses. Immunity *55*, 512–526.e9.
77. Ehlert, K., Hansjuergens, I., Zinke, A., Otto, S., Siebert, N., Henze, G., and Lode, H. (2020). Nivolumab and dinutuximab beta in two patients with refractory neuroblastoma. J. Immunother. Cancer *8*, e000540.
78. Man, K., Gabriel, S.S., Liao, Y., Gloury, R., Preston, S., Henstridge, D.C., Pellegrini, M., Zehn, D., Berberich-Siebelt, F., Febbraio, M.A., et al. (2017). Transcription Factor IRF4 Promotes CD8(+) T Cell Exhaustion and Limits the Development of Memory-like T Cells during Chronic Infection. Immunity *47*, 1129–1141.e5.
79. Percie du Sert, N., Hurst, V., Ahluwalia, A., Alam, S., Avey, M.T., Baker, M., Browne, W.J., Clark, A., Cuthill, I.C., Dirnagl, U., et al. (2020). The ARRIVE guidelines 2.0: Updated guidelines for reporting animal research. PLoS Biol. *18*, e3000410.
80. Workman, P., Aboagye, E.O., Balkwill, F., Balmain, A., Bruder, G., Chaplin, D.J., Double, J.A., Everitt, J., Farningham, D.A.H., Glennie, M.J., et al. (2010). Guidelines for the welfare and use of animals in cancer research. Br. J. Cancer *102*, 1555–1577.
81. Bate-Eya, L.T., Ebus, M.E., Koster, J., den Hartog, I.J.M., Zwijnenburg, D.A., Schild, L., van der Ploeg, I., Dolman, M.E.M., Caron, H.N., Versteeg, R., and Molenaar, J.J. (2014). Newly-derived neuroblastoma cell lines propagated in serum-free media recapitulate the genotype and phenotype of primary neuroblastoma tumours. Eur. J. Cancer *50*, 628–637.
82. M Kholosy, W., Derieppe, M., van den Ham, F., Ober, K., Su, Y., Custers, L., Schild, L., M J van Zogchel, L., M Wellens, L., R Ariese, H., et al. (2021). Neuroblastoma and DIPG Organoid Coculture System for Personalized Assessment of Novel Anticancer Immunotherapies. J. Pers. Med. *11*, 869.
83. Muraro, M.J., Dharmadhikari, G., Grün, D., Groen, N., Dielen, T., Jansen, E., van Gurp, L., Engelse, M.A., Carlotti, F., de Koning, E.J.P., and van Oudenaarden, A. (2016). A Single-Cell Transcriptome Atlas of the Human Pancreas. Cell Syst. *3*, 385–394.e3.
84. Candelli, T., Lijnzaad, P., Muraro, M.J., Kerstens, H., Kemmeren, P., van Oudenaarden, A., Margaritis, T., and Holstege, F. (2018). Sharq, A Versatile Preprocessing and QC Pipeline for Single Cell RNA-Seq. Preprint at bioRxiv. https://doi.org/10.1101/250811.
85. Yang, S., Corbett, S.E., Koga, Y., Wang, Z., Johnson, W.E., Yajima, M., and Campbell, J.D. (2020). Decontamination of ambient RNA in single-cell RNA-seq with DecontX. Genome Biol. *21*, 57.
86. Calandrini, C., Schutgens, F., Oka, R., Margaritis, T., Candelli, T., Mathijsen, L., Ammerlaan, C., van Ineveld, R.L., Derakhshan, S., de Haan, S., et al. (2020). An organoid biobank for childhood kidney cancers that captures disease and tissue heterogeneity. Nat. Commun. *11*, 1310.
87. Aran, D., Looney, A.P., Liu, L., Wu, E., Fong, V., Hsu, A., Chak, S., Naikawadi, R.P., Wolters, P.J., Abate, A.R., et al. (2019). Reference-based analysis of lung single-cell sequencing reveals a transitional profibrotic macrophage. Nat. Immunol. *20*, 163–172.
88. Novershtern, N., Subramanian, A., Lawton, L.N., Mak, R.H., Haining, W.N., McConkey, M.E., Habib, N., Yosef, N., Chang, C.Y., Shay, T., et al. (2011). Densely interconnected transcriptional circuits control cell states in human hematopoiesis. Cell *144*, 296–309.
89. Monaco, G., Lee, B., Xu, W., Mustafah, S., Hwang, Y.Y., Carré, C., Burdin, N., Visan, L., Ceccarelli, M., Poidinger, M., et al. (2019). RNA-Seq Signatures Normalized by mRNA Abundance Allow Absolute Deconvolution of Human Immune Cell Types. Cell Rep. *26*, 1627–1640.e7.
90. Zavidij, O., Haradhvala, N.J., Mouhieddine, T.H., Sklavenitis-Pistofidis, R., Cai, S., Reidy, M., Rahmat, M., Flaifel, A., Ferland, B., Su, N.K., et al. (2020). Single-cell RNA sequencing reveals compromised immune microenvironment in precursor stages of multiple myeloma. Nat. Cancer *1*, 493–506.
91. Szabo, P.A., Levitin, H.M., Miron, M., Snyder, M.E., Senda, T., Yuan, J., Cheng, Y.L., Bush, E.C., Dogra, P., Thapa, P., et al. (2019). Single-cell transcriptomics of human T cells reveals tissue and activation signatures in health and disease. Nat. Commun. *10*, 4706.
92. Tang-Huau, T.-L., Gueguen, P., Goudot, C., Durand, M., Bohec, M., Baulande, S., Pasquier, B., Amigorena, S., and Segura, E. (2018). Human *in vivo*-generated monocyte-derived dendritic cells and macrophages cross-present antigens through a vacuolar pathway. Nat. Commun. *9*, 2570.
93. Zhao, J., Guo, C., Xiong, F., Yu, J., Ge, J., Wang, H., Liao, Q., Zhou, Y., Gong, Q., Xiang, B., et al. (2020). Single cell RNA-seq reveals the landscape of tumor and infiltrating immune cells in nasopharyngeal carcinoma. Cancer Lett. *477*, 131–143.
94. Tirosh, I., Izar, B., Prakadan, S.M., Wadsworth, M.H., 2nd, Treacy, D., Trombetta, J.J., Rotem, A., Rodman, C., Lian, C., Murphy, G., et al. (2016). Dissecting the multicellular ecosystem of metastatic melanoma by single-cell RNA-seq. Science *352*, 189–196.
95. Subramanian, A., Tamayo, P., Mootha, V.K., Mukherjee, S., Ebert, B.L., Gillette, M.A., Paulovich, A., Pomeroy, S.L., Golub, T.R., Lander, E.S., and Mesirov, J.P. (2005). Gene set enrichment analysis: A knowledge-based approach for interpreting genome-wide expression profiles. Proc. Natl. Acad. Sci. USA *102*, 15545–15550.

96. Edgar, R., Domrachev, M., and Lash, A.E. (2002). Gene Expression Omnibus: NCBI gene expression and hybridization array data repository. Nucleic Acids Res. *30*, 207–210.

97. Uhlén, M., Fagerberg, L., Hallström, B.M., Lindskog, C., Oksvold, P., Mardinoglu, A., Sivertsson, Å., Kampf, C., Sjöstedt, E., Asplund, A., et al. (2015). Proteomics. Tissue-based map of the human proteome. Science *347*, 1260419.

98. Cornel, A.M., Dunnebach, E., Hofman, D.A., Das, S., Sengupta, S., van den Ham, F., Wienke, J., Strijker, J.G.M., van den Beemt, D.A.M.H., Essing, A.H.W., et al. (2022). Epigenetic modulation of neuroblastoma enhances T cell and NK cell immunogenicity by inducing a tumor-cell lineage switch. J. Immunother. Cancer *10*, e005002.

99. Scholman, R.C., Giovannone, B., Hiddingh, S., Meerding, J.M., Malvar Fernandez, B., van Dijk, M.E.A., Tempelman, M.J., Prakken, B.J., and de Jager, W. (2018). Effect of anticoagulants on 162 circulating immune related proteins in healthy subjects. Cytokine *106*, 114–124.

100. Dobin, A., Davis, C.A., Schlesinger, F., Drenkow, J., Zaleski, C., Jha, S., Batut, P., Chaisson, M., and Gingeras, T.R. (2013). STAR: ultrafast universal RNA-seq aligner. Bioinformatics *29*, 15–21.

101. Harrow, J., Frankish, A., Gonzalez, J.M., Tapanari, E., Diekhans, M., Kokocinski, F., Aken, B.L., Barrell, D., Zadissa, A., Searle, S., et al. (2012). GENCODE: the reference human genome annotation for The ENCODE Project. Genome Res. *22*, 1760–1774.

102. Dessau, R.B., and Pipper, C.B. (2008). [''R"–project for statistical computing]. Ugeskr. Laeger *170*, 328–330.

103. D'Eustachio, P. (2011). Reactome knowledgebase of human biological pathways and processes. Methods Mol. Biol. *694*, 49–61.

104. Efremova, M., Vento-Tormo, M., Teichmann, S.A., and Vento-Tormo, R. (2020). CellPhoneDB: inferring cell-cell communication from combined expression of multi-subunit ligand-receptor complexes. Nat. Protoc. *15*, 1484–1506.

## STAR★METHODS

### KEY RESOURCES TABLE

| REAGENT or RESOURCE | SOURCE | IDENTIFIER |
|---|---|---|
| Antibodies | | |
| Fixable viability dye efluor 506 | ThermoFisher | Cat# 65-0866-14 |
| Mouse Anti-CCL2 PE-Cy7 | Biolegend | Cat# 502613; RRID: AB_2734490 |
| Mouse Anti-CCR7 PE-Fire810 | Biolegend | Cat# 353269; RRID: AB_2894572 |
| Mouse Anti-CD123 APC-Fire810 | Biolegend | Cat# 306053; RRID: AB_2904334 |
| Mouse Anti-CD127 Spark NIR 685 | Biolegend | Cat# 351361; RRID: AB_2860913 |
| Mouse Anti-CD163 BUV563 | BD Biosciences | Cat# 752879; RRID: AB_2917834 |
| Mouse Anti-CD19 BV480 | BD Biosciences | Cat# 566164; RRID: AB_2739561 |
| Mouse Anti-CD1c APC-R700 | BD Biosciences | Cat# 566615; RRID: AB_2869795 |
| Mouse Anti-CD3 APC-Cy7 | Biolegend | Cat# 344817; RRID: AB_10644011 |
| Mouse Anti-CD3 BUV615 | BD Biosciences | Cat# 612993; RRID: AB_2870264 |
| Mouse Anti-CD31 BV711 | Biolegend | Cat# 303135; RRID: AB_2716212 |
| Mouse Anti-CD3 PE | BioLegend | Cat# 317308; RRID: AB_571913 |
| Mouse Anti-CD4 BUV496 | BD Biosciences | Cat# 612937; RRID: AB_2916881 |
| Mouse Anti-CD4 cYG584 | Cytek | Cat# SKU R7-20042 |
| Mouse Anti-CD4 FITC | Biolegend | Cat# 357406; RRID: AB_2562357 |
| Mouse Anti-CD45 BUV395 | BD Biosciences | Cat# 563791; RRID: AB_2744400 |
| Mouse Anti-CD56 PE-Cy5 | Biolegend | Cat# 318308; RRID: AB_604105 |
| Mouse Anti-CD56 APC | Biolegend | Cat# 318310; RRID: AB_604098 |
| Recombinant Anti-CD68 APC-Vio770 | Miltenyi | Cat# 130-114-654; RRID: AB_2726730 |
| Mouse Anti-CD74 BV786 | BD Biosciences | Cat# 743736; RRID: AB_2741709 |
| Mouse Anti-CD8 PerCP | Biolegend | Cat# 980916; RRID: AB_2890877 |
| Mouse Anti-CD8 PE | Biolegend | Cat# 344706; RRID: AB_1953244 |
| Mouse Anti-CD96 BV421 | Biolegend | Cat# 338417; RRID: AB_2629536 |
| Mouse Anti-CTLA-4 APC | Biolegend | Cat# 349907; RRID: AB_10680785 |
| Mouse Anti-GD2 FITC | Biolegend | Cat# 563439; RRID: AB_2738206 |
| Mouse Anti-GD2 BV750 | BD Biosciences | Cat# 746863; RRID: AB_2871665 |
| Mouse Anti-GZMB RY586 | BD Biosciences | Cat# 568134 |
| Mouse Anti-ICOS PE-Cy5.5 | ThermoFisher | Cat# 35-9948-41; RRID: AB_2815129 |
| Rat Anti-IL10 BB700 | BD Biosciences | Cat# 566568; RRID: AB_2869786 |
| Mouse Anti-IL1B BV421 | BD Biosciences | Cat# 567792 |
| Mouse Anti-Ki-67 BV650 | BD Biosciences | Cat# 563757; RRID: AB_2688008 |
| Mouse Anti-LAG3 APC-R700 | BD Biosciences | Cat# 565775; RRID: AB_2744329 |
| Mouse Anti-cMAF eF450 | ThermoFisher | Cat# 48-9855-42; RRID: AB_2762608 |
| Recomb Anti-NECTIN2 PE-Vio615 | Miltenyi | Cat# 130-122-784; RRID: AB_2819430 |
| Mouse Anti-PD-1 BV605 | BD Biosciences | Cat# 563245; RRID: AB_2738091 |
| Mouse Anti-PD-L1 PE-Fire810 | Biolegend | Cat# 329755; RRID: AB_2894668 |
| Mouse Anti-Perforin PE-Cy7 | Biolegend | Cat# 308125; RRID: AB_2572049 |
| Mouse Anti-PVR AF647 | Biolegend | Cat# 748275; RRID: AB_2872703 |
| Mouse Anti-S100A9 APC | Biolegend | Cat# 565833; RRID: AB_2739373 |
| Mouse Anti-TCRgd APC-Vio770 | Miltenyi | Cat# 130-114-027; RRID: AB_2751186 |
| Mouse Anti-TCRgd BV421 | Biolegend | Cat# 331217; RRID: AB_2562317 |
| Mouse Anti-TIGIT PE | Biolegend | Cat# 372704; RRID: AB_2632729 |
| Mouse Anti-TIGIT BB700 | BD Biosciences | Cat# 747846; RRID: AB_2872309 |
| Mouse Anti-TIM-3 AF647 | BD Biosciences | Cat# 565559; RRID: AB_2744367 |

(*Continued on next page*)

*Continued*

| REAGENT or RESOURCE | SOURCE | IDENTIFIER |
|---|---|---|
| Mouse Anti-CD25 APC-Fire810 | Biolegend | Cat# 356149; RRID: AB_2876679 |
| Rat Anti-FOXP3 eF450 | ThermoFisher | Cat# 48-4776-42; RRID: AB_1834364 |
| Fixable viability dye UV blue | Thermofisher | Cat# L23105 |
| Anti-CD19 BUV563 | BD Biosciences | Cat# 749028; RRID: AB_2873425 |
| Anti-CD4 BUV737 | BD Biosciences | Cat# 612761; RRID: AB_2870092 |
| Anti-MHCII Vioblue | Miltenyi | Cat# 130-112-394; RRID: AB_2652908 |
| Anti-CD11b BV605 | Biolegend | Cat# 101257; RRID: AB_2565431 |
| Anti-F4/80 BV711 | Biolegend | Cat# 123147; RRID: AB_2564588 |
| Anti-Ly6C BV785 | Biolegend | Cat# 128041 |
| Anti-CD45 FITC | Biolegend | Cat# 103107; RRID: AB_312972 |
| Anti-CD8a NovaFluor™ Blue 660-120S | ThermoFisher | Cat# M003T02B08; RRID: AB_2896728 |
| Anti-CD38 PE | Miltenyi | Cat# 130-102-607; RID:AB_2657877 |
| Anti-LY6G PE-dazzle594 | Biolegend | Cat# 127647; RRID: AB_2566318 |
| Anti-CD3 PE-Fire700 | Biolegend | Cat# 100271; RRID: AB_2876394 |
| Anti-muB7H3 PE-Cy7 | Biolegend | Cat# 135613; RRID: AB_2800636 |
| Anti-GD2 APC | Biolegend | Cat# 357305; RRID: AB_2563083 |
| Anti-CD206 Alexa Fluor 700 | Biolegend | Cat# 141734; RRID: AB_2629637 |
| Anti-CD49b APC-Vio770 | Miltenyi | Cat# 130-105-249; RRID: AB_2660464 |
| Anti-CD3 (IHC) | Leica | Cat# PA0553 |
| Anti-SOX10 (IHC) | Cell Marque | Cat# 383R-18 |
| Anti-TIGIT | Abcam | Cat# 233404; RRID: AB_2827380 |
| Anti-PD-1 | Cell Signaling Technology | Cat# 64651 |
| Anti-PD-L1 | Cell Signaling Technology | Cat# 64988s |
| Mouse Anti-TGFβ | eBioscience | Cat# 16-9243-85; RRID: AB_2573124 |
| Anti-TIGIT (Tiragolumab) | Roche | Cat# N/A |
| Anti-PD-1 (Atezolizumab) | Roche | Cat# N/A |
| Anti-GD2 (Dinutuximab) | BioXcell | Cat# BE0318; RRID: AB_2819045 |
| **Biological samples** | | |
| Pediatric patient tumor tissue | This paper, Princess Máxima Center for Pediatric Oncology | N/A |
| Healthy donor blood samples | This paper, University Medical Center Utrecht | N/A |
| Healthy donor buffy coats | Sanquin | N/A |
| PRAME-TCR transduced T cells | Dr. Stefan Nierkens; Cornel et al.[98] | N/A |
| **Chemicals, peptides, and recombinant proteins** | | |
| Recombinant Human TGFβ | Preprotech | Cat# 100-21-10UG |
| Haematoxylin | BOOM BV | Cat# 840.154.912.500 |
| Eosine Y alcoholic | VWR International | Cat# 10047103 |
| Collagenase I | ThermoFisher | Cat# 17100017 |
| Collagenase II | ThermoFisher | Cat# 17100015 |
| Collagenase IV | ThermoFisher | Cat# 17100019 |
| Dulbecco's modified Eagle's medium (DMEM) with low glucose and Glutamax™ | ThermoFisher | Cat# 21885-108 |
| Ham's F-12 Nutrient Mix | ThermoFisher | Cat# 31765027 |
| B-27 supplement minus vitamin A (50×) | ThermoFisher | Cat# 12587010 |
| N-2 supplement (100×) | ThermoFisher | Cat# 17502048 |
| Recombinant human EGF | Peprotech | Cat# AF-100-15 |
| Recombinant human FGF-2 | Peprotech | Cat# 100-18B |
| Recombinant human IGF-1 | R&D | Cat# 100-11 |

(*Continued on next page*)

*Continued*

| REAGENT or RESOURCE | SOURCE | IDENTIFIER |
|---|---|---|
| Recombinant human PDGF-AA | Peprotech | Cat# 100-13A |
| Recombinant human PDGF-BB | Peprotech | Cat# 100-14B |
| NeuroCult dissociation kit | Stemcell™ Technologies | Cat# 05707 |
| DAPI | Miltenyi Biotec | Cat# 130-111-570 |
| DRAQ5 | Sigma Aldrich | Cat# GE17-1440-03 |
| Ficoll-Paque | Sigma Aldrich | Cat# GE17-1440-03 |
| Accutase | Sigma Aldrich | Cat# A6964-100ML |
| RPMI 1640 | Gibco | Cat# 21875091 |
| L-Glutamine | ThermoFisher | Cat# 25030 |
| D-luciferin | Perkin Elmer | Cat# 122799 |
| Temozolomide | Sigma-Aldrich | Cat# #T2577 |
| Irinotecan | Sigma-Aldrich | Cat# #I1406 |
| Vincristine sulfate | Sigma-Aldrich | Cat# V8879 |
| Doxorubicin hydrochloride | Sigma-Aldrich | Cat# D1515 |
| Cyclophosphamide monohydrate | Sigma-Aldrich | Cat# C0768 |
| IL-2 | Miltenyi | Cat# 130-097-744 |
| IL-15 | Miltenyi | Cat# 130-095-762 |
| **Critical commercial assays** | | |
| Foxp3/Transcription Factor Staining Buffer Set | eBioscience | Cat# 00-5523-00 |
| **Deposited data** | | |
| Single-cell RNA sequencing data of 24 tumors and 5 healthy controls | This paper | GSE218003; https://www.ncbi.nlm.nih.gov/geo/query/acc.cgi?&acc=GSE218003 |
| Single-cell RNA sequencing data of 24 tumors | This paper | EGAD00001009766; https://ega-archive.org/datasets/EGAD00001009766 |
| **Experimental models: Cell lines** | | |
| Neuro-2a cell line | Hamprecht | RRID: CVCL_0470 |
| N1E-115 cell line | Hamprecht | RRID: CVCL_0451 |
| N18 cell line | Hamprecht | RRID: CVCL_4724 |
| Patient-derived tumoroids AMC691B transduced with eGFP-luciferase | Amsterdam University Medical Center and Princess Máxima Center Utrecht; Bate-Eya et al.; Kholosy et al.[81,82] | N/A |
| Patient-derived tumoroid AMC691T transduced with eGFP-luciferase | Amsterdam University Medical Center and Princess Máxima Center Utrecht; Bate-Eya et al.; Kholosy et al.[81,82] | N/A |
| **Experimental models: Organisms/strains** | | |
| Mouse: A/Jax-000646 | The Jackson Laboratory | RRID: IMSR_JAX:000646 |
| Mouse: Th-ALK$^{F1174L}$/MYCN 129/SvJ | ICR[57] | N/A |
| **Software and algorithms** | | |
| Sharq pipeline | Candelli et al.[84] | N/A |
| STAR version 2.6.1 | http://code.google.com/p/rna-star/; Dobin et al.[100] | RRID: SCR_004463 |
| GENCODE version 26 | https://www.gencodegenes.org; Harrow et al.[101] | RRID: SCR_014966 |
| DecontX | Yang et al.[85] | N/A |
| R version 4.0.2 | http://www.r-project.org/; Dessau et al.[102] | RRID: SCR_001905 |
| Seurat version 3.2.2 | http://seurat.r-forge.r-project.org/ | RRID: SCR_007322 |
| SingleR version 1.2.4 | Aran et al.[87] | N/A |

(*Continued on next page*)

*Continued*

| REAGENT or RESOURCE | SOURCE | IDENTIFIER |
|---|---|---|
| ggplot2 version 3.3.2 | https://cran.r-project.org/web/packages/ggplot2/index.html | RRID: SCR_014601 |
| Reactome | https://reactome.org; D'Eustachio et al.[103] | RRID: SCR_003485 |
| GSEA broad institute | http://www.broadinstitute.org/gsea/; Subramanian et al.[95] | RRID: SCR_003199 |
| GEO2R | Edgar et al.[96] | N/A |
| CellChat | https://github.com/sqjin/CellChat; Williams et al.[54] | RRID: SCR_021946 |
| CellPhoneDB | http://www.cellphonedb.org/; Efremova et al.[104] | RRID: SCR_017054 |
| Flowjo version 10.8.1 | LLC | RRID: SCR_008520 |
| Graphpad Prism | GraphPad Software | RRID: SCR_002798 |
| Code | This paper | https://bitbucket.org/princessmaximacenter/neuroblastoma_nectin2_tigit/src/master/ |

## RESOURCE AVAILABILITY

### Lead contact

Further information and requests for resources and reagents should be directed to and will be fulfilled by the lead contact, Judith Wienke (j.wienke-4@prinsesmaximacentrum.nl).

### Materials availability

This study did not generate new unique reagents.

### Data and code availability

Single-cell RNA-seq data have been deposited at GEO and EGA and are publicly available as of the date of publication. EGA only includes the patient samples, GEO also includes the healthy control data. Accession numbers are listed in the key resources table. All original code has been deposited at Bitbucket and is publicly available as of the date of publication. DOIs are listed in the key resources table. Any additional information required to reanalyze the data reported in this paper is available from the lead contact upon request.

## EXPERIMENTAL MODEL AND STUDY PARTICIPANT DETAILS

### Human participants

From the Princess Máxima Center in Utrecht, The Netherlands, 19 patients with neuroblastoma were included between September 2017 and November 2020. Samples were obtained from patients during diagnostic biopsy pre-treatment (n=10) or during surgical resection after ca. 5 months of chemotherapeutic treatment (n=14). Demographic (e.g. sex, age, race), clinical (e.g. treatment) and histological information, including tumor staging by INRG, INRGSS and INSS was collected (Table S1). Information on socioeconomic status was unavailable as this is not regularly recorded in patient files. We did not perform separate analyses based on sex or gender as the relatively small sample size did not allow for this stratification. This might limit the generalizability of our study. The presence of MYCN amplification was determined by fluorescence *in situ* hybridization at the pathology department. 5 adult healthy volunteers donated blood for analysis of healthy immune cells and 8 buffy coats from the blood bank Sanquin were used for *in vitro* assays.

Studies underlying this paper have received appropriate approval by ethics review boards as per national legislation. Dutch tumor samples were obtained through an institutionally approved research study by the institutional review board of the Erasmus Medical Center Rotterdam (MEC-2016-793), the institutional review board for biobank at the University Medical Center Utrecht (TC18-774) and the biobank committee of the Princess Máxima Center (PMCLAB2020.093, PMCLAB2020.124 and PMCLAB2019.067). The studies were conducted in patients in accordance with the Declaration of Helsinki. Age-appropriate written informed consent from patients and/or parents was obtained prior to inclusion of each patient in the study.

### Animals

All experiments were approved by the Institute of Cancer Research Animal Welfare and Ethical Review Body and performed in accordance with the UK Home Office Animals (Scientific Procedures) Act 1986, the United Kingdom National Cancer Research Institute guidelines for the welfare of animals in cancer research and the ARRIVE guidelines.[79,80]

For *in vivo* experiments with syngeneic mouse tumor models Neuro-2a, N1E-115 and N18, approximately six to eight-week-old female A/Jax-000646 mice were ordered from The Jackson Laboratory. The animals had a starting body weight of 21±1.7 (mean±SD) gram.

The chemotherapy-resistant model was generated by allograft of neurospheres derived from *Th-ALK*$^{F1174L}$/*MYCN* 129/SvJ transgenic spontaneous tumors into syngeneic animals, which were then subjected to repeat and escalating chemotherapy (VAC – Vincristine, Adriamycin/Doxorubicin, Cyclophosphamide) (detailed in Figures S9A–S9F). The Th-ALKF1174L mutation was introduced as described previously[57] and Th-ALKF1174L founders were derived from CBA× C57BL/6J mice and genetically crossed with Th-MYCN mice of the 129/SvJ strain. Mice were bred at ICR. For treatment with ICB, the 129 SVJ mouse colony was maintained at UCL ICH Animal Facility. Animals were randomized for treatment using a Latin Square based on tumor volume, as measured by callipers. Mice were all female, and tumors were engrafted between 6-12 weeks of age.

Animals were housed in autoclaved, individually ventilated cages on racks, with a maximum stocking density of 5 animals per cage, 4 for males. Rooms were light cycled, temperature and humidity controlled, and quarterly health screening was performed to maintain a specific-pathogen-free status. Mice were allowed access to sterile food and water *ad libitum*. For the duration of the treatment with TEM/IRI, mice were given a damp diet in addition to the standard chow, to minimize the likelihood of chemotherapy-driven gastrointestinal toxicity. The damp diet was started on the day before TEM/IRI treatment start and terminated on the final day of TEM/IRI treatment. Animals were then returned to a diet of standard chow only.

### Tumoroids

Patient-derived tumoroids (MHC-1$^+$ AMC691T and MHC-1$^-$ AMC691B) were generated as described previously[81] from a female patient. In short, tumor pieces were minced, enzymatically digested with collagenase I, II and IV to achieve a tumor digest. This digest was cultured at 37°C with 5% $CO_2$ in neuroblastoma tumoroid medium, consisting of Dulbecco's modified Eagle's medium (DMEM) with low glucose and Glutamax™, supplemented with 20% Ham's F-12 Nutrient Mix, B-27 supplement minus vitamin A (50×), N-2 supplement (100×), 100 U/mL penicillin, 100 μg/mL streptomycin, 20 ng/mL animal-free recombinant human EGF, 40 ng/mL recombinant human FGF-basic, 200 ng/mL recombinant human IGF-I, 10 ng/mL recombinant human PDGF-AA and 10 ng/mL recombinant human PDGF-BB, until successful sphere formation and expansion of tumor cells. After generation, the tumoroids were frozen in medium with 10% DMSO until further use. For experiments, tumoroids were thawed and cultured in neuroblastoma tumoroid medium at 37°C with 5% $CO_2$ which was refreshed twice per week. Depending on the growth rate, cells were subcultured 1:2 once or twice per week. Cell authentication was performed regularly by STR profiling. The patient-derived tumoroids AMC691T and AMC691B with GFP-luciferase constructs were generated from these lines previously and viably frozen until further use.[82]

## METHOD DETAILS

### Isolation of cells from tumor biopsies

Tumor material was collected in the operating room by Tru-cut biopsy (at diagnosis) or surgical resection (after induction chemotherapy). Preparation of the tumor pieces was started within 4 hours after surgery. The material was minced into pieces <1mm$^3$ and dissolved by enzymatic digestion with collagenase I, II and IV (2.5 mg/mL) at 37°C with agitation for max. 1 hour, filtered through a 70 μm cell strainer and washed in DMEM. Cells were further separated into a single cell suspension with the NeuroCult dissociation kit according to the manufacturer's protocol (Stemcell™ Technologies, cat#05707), washed and stained with DAPI and DRAQ5. Some samples were additionally stained with antibodies to enrich for T cells (NB124 and NB125 with CD3-PE; NB124 with TCRγδ-BV421) or for tumor cells (000GGU, 000GXF, NB106, NB107, NB098, NB125, NB125, NB130, NB152 with anti-GD2). Single live cells were FACS sorted into 384-well plates containing 10 μL of mineral oil, 50 nL of Reverse Transcription primers, deoxynucleotide triphosphates (dNTPs) and synthetic mRNA Spike-Ins on a FACSJazz, FACSAria II or Sony SH800S machine and subsequently spun down, snap-frozen on dry ice, and stored at -80°C until further use.

### Isolation of cells from peripheral blood

For single-cell RNA sequencing, peripheral blood mononuclear cells (PBMC) from 5 healthy young adult donors (mean age 28, 2 male, 3 female) were freshly isolated by Ficoll-Paque density centrifugation and stained with fixable viability dye efluor506, CD3-PE and CD56-APC antibodies. Live CD3$^+$ T cells and CD56$^+$ NK cells were FACS sorted into 384-well plates containing 10 μl of mineral oil, 50 nl of RT primers, deoxynucleotide triphosphates (dNTPs) and synthetic mRNA Spike-Ins on a Sony SH800S machine. The plates were spun down, snap-frozen on dry ice, and stored at -80°C until further use.

For killing assays, PBMC from 8 healthy donors (3 for full PBMC, 3 for immune subsets, and 2 for NK cells) were isolated from buffy coats by Ficoll-Paque density centrifugation. The latter cells were either stained with TCRγδ-BV421, CD4-FITC, CD8-PE, CD56-APC, and CD3-APC-Cy7 antibodies (for immune subsets) or with CD3-PE and CD56-APC (for NK cells) and FACS sorted on a Sony SH800S machine.

### Immunohistochemistry

Immunohistochemistry of human neuroblastoma paraffin-embedded tissue slides with hematoxylin & eosine, anti-CD3 and anti-SOX10 was performed by the pathology department of the PMC. For CD3 and SOX10 staining, tissue slides were pre-treated with a citrate solution for 20 minutes at 100°C, and incubated with the antibodies for 15 minutes at room temperature. The stainings

were performed on a BOND immunostainer and visualized with the BOND polymer refine detection kit with a DAB enhancer. The stained slides were subsequently annotated by our neuroblastoma-specialized pathologist. Immunohistochemical staining of mouse neuroblastoma tumors was performed using anti-TIGIT, anti-PD1 and anti-PD-L1 antibodies at WuXi Biologics.

### CEL-Seq2 library preparation, sequencing & mapping

All samples were processed for total transcriptome amplification, library preparation and sequencing into Illumina sequencing libraries as previously described.[83] Paired-end 2x75 bp sequencing read length was used to sequence the prepared libraries using the Illumina NextSeq sequencer. Sharq preprocessing and QC pipeline were applied to process the single-cell RNA-seq data as described.[84] Read mapping was done using STAR version 2.6.1 (RRID: SCR_004463) on the hg38 Patch 10 human genome. Function featureCounts (RRID: SCR_012919) of the *subread* package (version 1.5.2) was used to assign reads based on GENCODE version 26 (RRID: SCR_014966).

### CEL-Seq2 quality control

Failed reactions were identified by low levels of ERCC external RNA controls and excluded.[84] Furthermore, a liveness threshold was calculated for each plate based on the wells with no cell added, in order to distinguish live cells from dead and/or apoptotic cells.[84] This threshold was set to a minimum of 500 transcripts. Genomic:protein-coding read ratio (GPratio) was calculated based on raw counts and cells with a GPratio <20 were removed.[84]

Next, the percentage of transcripts mapping to the mitochondrial genome was calculated and cells with more mitochondrial-encoded transcripts over nuclear ones were removed. Mitochondrial and ERCC transcripts were removed from the dataset, as well as cells with <1000 nuclear-encoded transcripts or <500 genes. In addition, cells with >150.000 nuclear-encoded transcripts were removed. Pseudogenes were removed, as well as genes with low expression – that is either having less than 5 cells expressing the gene or less than two cells with less than two transcripts. A distinct cluster of erythroid lineages was identified based on high levels of hemoglobin complex genes, and was removed. Between-sample variation was minimized by using a standard operating procedure for sample preparation (described above). To further improve cross-sample comparisons, ambient mRNA contamination in individual cells was estimated and removed using DecontX.[85] DecontX was run for all samples (batches) individually. Removal of cells with less than 1000 nuclear-encoded transcripts was repeated on the decontaminated counts matrix outputted by DecontX. Afterwards, one cluster still showed a higher contamination score (>0.2), as compared to the other clusters, and was additionally removed.

### Single-cell RNA sequencing cluster identification

All subsequent analyses were performed using R (version 4.0.2) and the package Seurat (version 3.2.2) with default parameters unless stated otherwise. The *SCTransform*() function was used to normalize and scale the data, and to identify variable genes. To avoid clustering of cells based on specific cell processes, genes associated with sex (*XIST*, *TSIX*, and Y chromosome-specific genes), cell cycle phase, dissociation stress (heat shock proteins; GO:0006986), and activity (ribosomal protein genes; GO:0022626), were removed as described before.[86]

Principal component analyses were performed using the filtered lists of variable genes. To study the main cell types in the tumor, the first 35 principal components (PCs) were used to calculate dimensionality reduction using UMAP, and a resolution of 0.5 was used to define clusters using the Louvain method. For immune cell-focused analysis immune cell clusters were subset based on PTPRC gene expression. For in-depth analyses, the respective clusters were subset and UMAP was rerun with an optimal number of PCs and resolution to define subclusters (T/NK: 45 PCs, resolution 0.6; myeloid: 35 PCs and resolution 0.8; mesenchyme: 40 PCs, resolution 0.2).

For the peripheral blood healthy control dataset the first 8 PCs were used for dimensionality reduction, and a resolution of 0.5 was used to define the clusters. For combined analysis of tumor and blood, the two Seurat objects were merged, after which 50 PCs and a resolution of 0.4 were used for dimensionality reduction and defining clusters. For in-depth analysis of the T and NK cells the respective clusters were subset from this combined dataset, UMAP was rerun using 35 PCs and a resolution of 0.5.

### Cluster annotation

Cluster annotation was performed with R package SingleR (version 1.2.4),[87] using the HumanPrimaryCellAtlas reference dataset for main cell types, and the *NovershternHematopoieticData*[88] and *MonacoImmuneData*[89] reference datasets for immune cell (sub)clusters. Cell annotations were further refined by consulting cluster-specific (up-regulated) differentially expressed marker genes using Seurat's *FindAllMarkers* function. The resulting genes were compared to known cell-type specific marker genes from previous studies.[33,90–93] Malignant and non-malignant cells were distinguished according to three criteria: (1) their inferred CNV profiles (*see below*);[94] (2) under-expression or absence of different non-malignant cell type marker genes; and (3) high expression of published neuroblastoma-associated genes (Figures 1D and S1B). For tumor cell identification by copy number variation inference the R package inferCNV was run (with default settings except cutoff=0.1, denoise=T and HMM=F) using the immune and endothelial cell clusters as a reference.

To mirror our identified subsets against the subsets of Costa et al. and Verhoeven et al., we used gene signatures from their datasets (see Table S2) and performed gene set enrichment analysis with the differentially expressed genes between subsets in our dataset (*Findallmarkers* with adjusted parameters, see code).

### Differential gene expression analysis

Cluster-specific genes were identified using the *Findallmarkers* function in Seurat (RRID:SCR_007322) and genes with padj<0.05 were considered differentially expressed. Differentially expressed genes between two groups were identified using the *Findmarkers* function and genes with padj<0.05 were considered differentially expressed, as indicated in the figure legends. Volcanoplots were created with the R package *EnhancedVolcano*.

### Cellular composition analysis

For analyses determining the composition of cell types of the individual tumor samples, we only included samples which were sorted in an unbiased manner (DAPI & DRAQ5 for total cells, overall immune cells, and myeloid cells; DAPI & DRAQ5 or anti-CD3 for T cell subsets). For immune cell composition analyses, samples with <10 immune cells were excluded. Barplots were generated using ggplot2 (version 3.3.2). Differences in composition between treatment-naive and treatment-exposed tumor samples were statistically tested using the Wilcoxon rank-sum test.

### Pathway and gene set enrichment analysis

Pathway enrichment analysis was conducted with the online Reactome portal (https://reactome.org) using differentially expressed genes with padj<0.05. Pathways with >10 identified genes and Bonferroni-corrected $P$<0.05 were considered statistically significant.

For GSEA, differential expression analysis was performed with *Findmarkers* for two groups, or *Findallmarkers* for >2 groups, using adjusted parameters (see code). Genes were pre-ranked by their Fold Change and GSEA was performed using Broad Institute software, by 1000 random permutations of the phenotypic subgroups to establish a null distribution of enrichment score, against which a normalized enrichment scores and multiple testing FDR-corrected $q$ values were calculated.[95] Gene sets with an FDR<0.25 were considered significantly enriched, as recommend by Broad Institute. Gene sets were either obtained from MSigDB, from provided data in publications or by analyzing raw data using GEO2R (NCBI tool).[96] An overview of used signatures is provided in Table S2.

### Interaction analysis

The CellChat algorithm was applied to perform an unbiased ligand-receptor interaction analysis, using the curated ligand-receptor database of CellPhoneDB.[52] To identify functionally relevant interactions, we overlaid 1) genes B expressed by cell subset X which were predicted to be membrane-expressed or secreted in the Human Protein Atlas database[97] and had a significant correlation (p<0.05) with the cytotoxicity score or exhaustion score of T/NK cell subset Y and 2) were predicted to have a significant interaction (p<0.05) between cell subset X and T/NK cell subset Y.

### Flow cytometry of neuroblastoma tumors

Tumor samples were minced, enzymatically digested as described above, but only with collagenase IV to preserve surface marker expression, and stored as cellular digest in FCS with 10% DMSO in liquid nitrogen until further use. Samples were thawed, washed twice with PBS, stained for 20 minutes at -4°C with Fixable viability dye eFluor 506 (eBioscience) subsequently washed in PBS, stained with the surface antibodies (see key resources table) for 20 minutes at 4°C, washed in staining buffer (PBS with 2% FCS and 2mM EDTA) and fixed and permeabilized with the Foxp3/Transcription Factor Staining Buffer Set (eBioscience) according to manufacturer's instructions. Cells were then stained with the intracellular and intranuclear antibodies for 30 minutes at 4°C, washed and measured on an Aurora (Cytek) instrument.

### *In vitro* tumor killing assays

Cultured tumoroids were dissociated into single cells with Accutase (Sigma) and mechanical dissociation by pipetting. 5000 cells per well were seeded in 100 µl tumoroid medium in a white flat-bottom TC-treated 96-well plate (Corning, cat#3917) and rested for 2-3 days to reform spheres at 37°C and 5% $CO_2$. Effector cells from healthy donors, i.e. isolated allogeneic PBMCs, immune subsets, NK cells or PRAME-TCR transduced T cells (the latter were kindly provided by the Nierkens group[98]), were added to the tumoroids at the indicated effector:target (E:T) ratios in RPMI 1640 (Gibco) supplemented with 10% FBS, 100 U/mL Penicillin/Streptomycin and 2mM% L-Glutamine, with or without anti-TIGIT (clone 10A7) and anti-PD-L1 (clone 6E11; both kindly provided by Roche; 10 ng/mL), recombinant human TGF-β (10 ng/mL), or anti-TGF-β antibody (1 µg/mL). NK cells were primed before the co-culture with IL-2 (1000 IU/mL) and IL-15 (50 ng/mL) overnight. After 6 days (PBMC, immune subsets and PRAME TCR-T cells) or 24 hours (NK cells), supernatants were collected for multiplex immunoassay (for measurement of granzyme B expression by NK cells)[99] and D-luciferin (150 µg/mL) was added to the culture and incubated for 5 minutes at 37°C. The produced luminescence signal was detected with the FLUOstar Omega microplate reader and normalized against an untreated tumoroid only control to calculate the percentage of tumor killing.

### TIGIT and PD-L1 blockade *in vivo*

*In vivo* experiments were carried out in Neuro-2a, N1E-115 and N18 syngeneic tumor models. Neuroblastoma cells were routinely subcultured twice weekly. The cells growing in an exponential growth were subcutaneously inoculated at $1x10^6$ cells/ml in 0.1 ml PBS for tumor development, and tumors were grown for 7 (N1E-115), 6 (Neuro2a) and 5 (N18) days before start of treatment, until average tumor volume was ~100 $mm^3$. On day 0 of treatment, body weight and tumor volume were measured, and mice were randomized into study groups with nine animals per arm. Mice were dosed IP with anti-mu-TIGIT (10 mg/kg, first dosing done i.v.) and

anti-PDL1 clone 6E11 (10mg/kg), supplied by Roche. Both molecules were administrated 3 times per week for 3 weeks. For TIL analysis tumors were harvested, weighed, and processed to obtain single-cell suspensions; tumor tissues were dissociated using MACS Dissociator (Miltenyi-130-093-235). All samples were analyzed by flow cytometry (BD FACS LSR Fortessa) and >10,000 $CD45^{+}$ cells were recorded for further analysis.

The chemotherapy-resistant model was generated by allograft of neurospheres derived from *Th-ALK*$^{F1174L}$/*MYCN* 129/SvJ transgenic spontaneous tumors into syngeneic animals, which were then subjected to repeat and escalating chemotherapy (VAC – Vincristine, Adriamycin/Doxorubicin, Cyclophosphamide). These chemotherapy-resistant tumors were subsequently allografted into untreated syngeneic animals which underwent treatment with TEM/IRI (Temozolomide: 3.2 mg/kg IP; Irinotecan: 1.6 mg/kg IP; daily on days 1 through 5:) + anti-GD2 (14G2a, 0.8 mg/kg IP on day 1 and day 5) with or without anti-TIGIT and/or anti-PD-L1 (10 mg/kg IP; twice weekly for three weeks). Mice with tumor volumes <50 mm3 (small tumors) or tumor volumes 50-200 mm3 (large tumors) at the start of treatment (day 10 after engraftment for small tumors and day 14-20 after engraftment for large tumors) were included in the analysis. For TME analysis, mice were culled by humane methods, tumors were harvested and dissected, and tumor pieces were cut by scalpel, washed twice with PBS, and put on a rotator in 5 mL accumax for 10 minutes. Cells were then pressed through a cell strainer and washed twice with PBS. The pellet was resuspended in 1 mL K lysis buffer for 5 minutes and washed twice with PBS. The cells were then counted using a CellCountess and stained according to manufacturer's instructions with antibodies, and run on a BD Symphony cytometer and matched software.

## QUANTIFICATION AND STATISTICAL ANALYSIS

Apart from the differential gene expression analysis described above, comparisons between two groups (e.g. for signatures) were made by Mann-Whitney U test. Comparisons between 3 or more groups were analyzed by Kruskal-Wallis with Dunn's post hoc test for multiple comparisons or by 2-way ANOVA for multi-level analyses, as indicated in the figure legends. For heatmap analysis, normalized gene expression was extracted from DotPlot analysis in Seurat, and hierarchical clustering was performed with Ward's method and Euclidian distance. For correlation analyses, Pearson correlation coefficients were computed. FACS data were analyzed using FlowJo V10.8.1 software (LLC). Statistical analysis of FACS data was performed with Graphpad Prism (GraphPad Software). For survival analysis, matched analysis using the log-rank (Mantel-Cox) test was performed. Differences in tumor volumes between treatment groups in *in vivo* studies were calculated by linear mixed-effects models analysis (see code).

Cancer Cell, Volume *42*

## Supplemental information

# Integrative analysis of neuroblastoma by single-cell RNA sequencing identifies the NECTIN2-TIGIT axis as a target for immunotherapy

**Judith Wienke, Lindy L. Visser, Waleed M. Kholosy, Kaylee M. Keller, Marta Barisa, Evon Poon, Sophie Munnings-Tomes, Courtney Himsworth, Elizabeth Calton, Ana Rodriguez, Ronald Bernardi, Femke van den Ham, Sander R. van Hooff, Yvette A.H. Matser, Michelle L. Tas, Karin P.S. Langenberg, Philip Lijnzaad, Anne L. Borst, Elisa Zappa, Francisca J. Bergsma, Josephine G.M. Strijker, Bronte M. Verhoeven, Shenglin Mei, Amira Kramdi, Restuadi Restuadi, Alvaro Sanchez-Bernabeu, Annelisa M. Cornel, Frank C.P. Holstege, Juliet C. Gray, Godelieve A.M. Tytgat, Marijn A. Scheijde-Vermeulen, Marc H.W.A. Wijnen, Miranda P. Dierselhuis, Karin Straathof, Sam Behjati, Wei Wu, Albert J.R. Heck, Jan Koster, Stefan Nierkens, Isabelle Janoueix-Lerosey, Ronald R. de Krijger, Ninib Baryawno, Louis Chesler, John Anderson, Hubert N. Caron, Thanasis Margaritis, Max M. van Noesel, and Jan J. Molenaar**

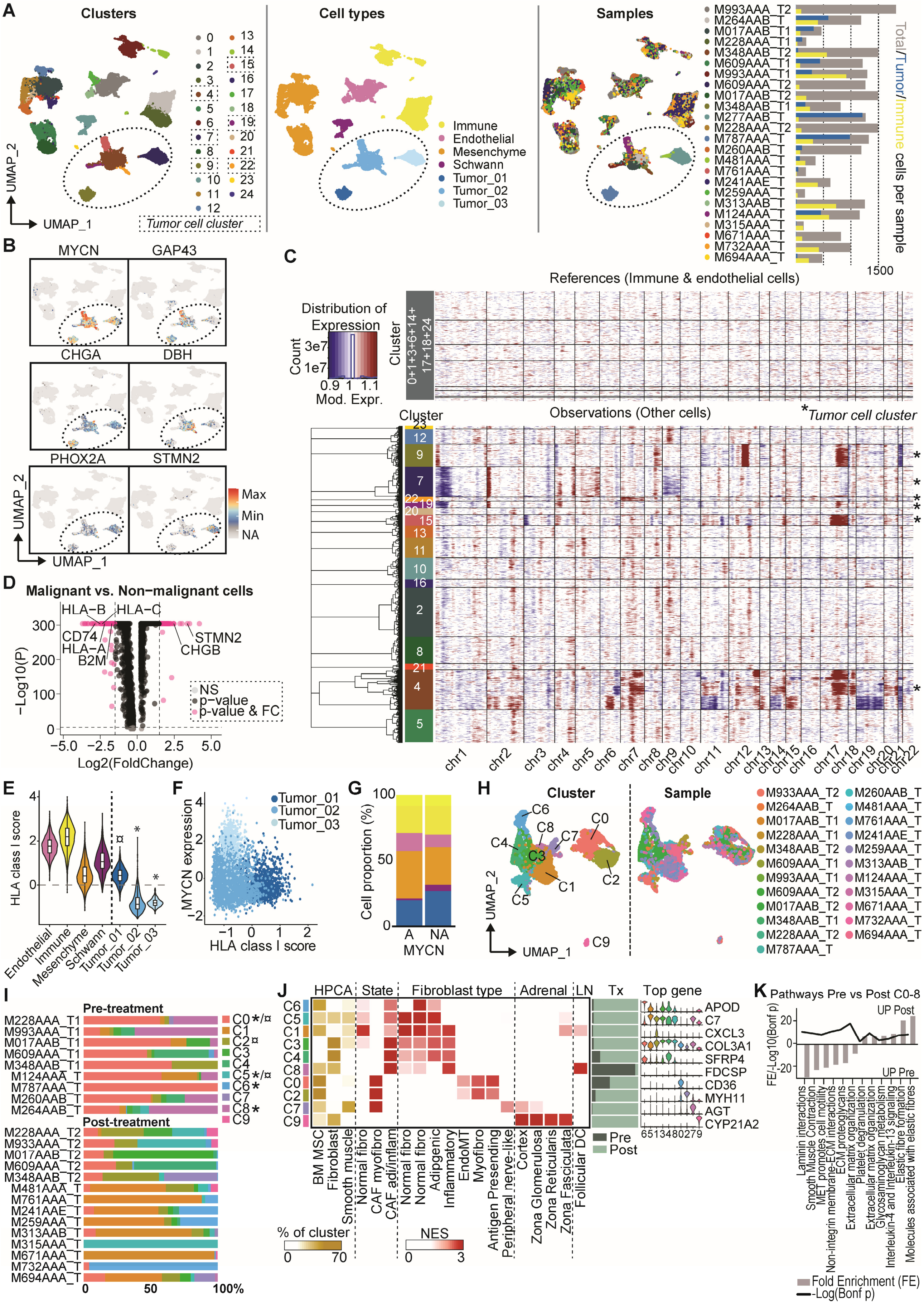

A
Clusters
Cell types
Samples
Immune
Endothelial
Mesenchyme
Schwann
Tumor_01
Tumor_02
Tumor_03
Tumor cell cluster
Total/Tumor/Immune cells per sample
B
MYCN
GAP43
CHGA
DBH
PHOX2A
STMN2
C
References (Immune & endothelial cells)
Distribution of Expression
Observations (Other cells)
*Tumor cell cluster
D
Malignant vs. Non−malignant cells
Log2(FoldChange)
−Log10(P)
E
HLA class I score
F
MYCN expression
G
Cell proportion (%)
H
Cluster
Sample
I
Pre-treatment
Post-treatment
J
HPCA
State
Fibroblast type
Adrenal
LN
Tx
Top gene
% of cluster
NES
K
Pathways Pre vs Post C0-8
UP Post
UP Pre
Fold Enrichment (FE)
-Log(Bonf p)

**Figure S1. The single-cell landscape of neuroblastoma, related to figure 1. (A)** UMAP of identified cell clusters, main cell types and sample distribution. Dotted circles highlight the tumor cell clusters. Right panel: number of total cells, tumor cells and immune cells per sample. **(B)** UMAP with expression of typical neuroblastoma genes (extension of Figure 1D). **(C)** Copy number profile of the samples, generated using *inferCNV*, to confirm tumor cell identity. The identified cell clusters (Figure S1A) were used as cell groups, in which immune and endothelial cell clusters were used as reference. *Clusters annotated as tumor cells based on gene expression profile. **(D)** Volcanoplot of differentially expressed genes (padj<0.05) between tumor cells (malignant) and all other cells (non-malignant). **(E)** Violin plot showing the modulescore of HLA class I genes *HLA-A*, *HLA-B*, *HLA-C*, *HLA-E*, *HLA-F* and *B2M* in the main cell types. *Kruskall-Wallis + Dunn's. °p<0.0001 versus all non-malignant clusters except mesenchyme, which was not signficant. *p<0.0001 versus all non-malignant clusters.* **(F)** Correlation between *MYCN* expression and HLA class I gene score for the three tumor clusters. **(G)** Cell proportion in MYCN-A versus MYCN-NA samples. **(H)** UMAP of mesenchyme clusters and sample distribution**. (I)** Proportion of different mesenchymal clusters per sample. "T1" and "T2" refer to paired samples before and after treatment, respectively. *Proportion significantly differs pre vs post treatment over all samples (p<0.05; Mann-Whitney U test). ¤Proportion significantly differs pre- vs post-treatment in paired samples (p<0.05; Wilcoxon matched-ranks test). **(J)** Annotation of mesenchyme clusters with signatures from human protein/cell atlas (HPCA), GSEA using signatures of CAF states and fibroblast types[1], signatures of adrenal cell types[2], and signatures of follicular dendritic cells (FDC)[3]. Distribution pre- vs post-treatment (Tx) per cluster and top differentially expressed gene per cluster. *NES = normalized enrichment score.* **(K)** Pathway analysis (Reactome; Bonferroni p-value<0.05) of combined clusters C0-8 of genes which were significantly upregulated (p<0.05) pre-treatment vs post-treatment using all samples.

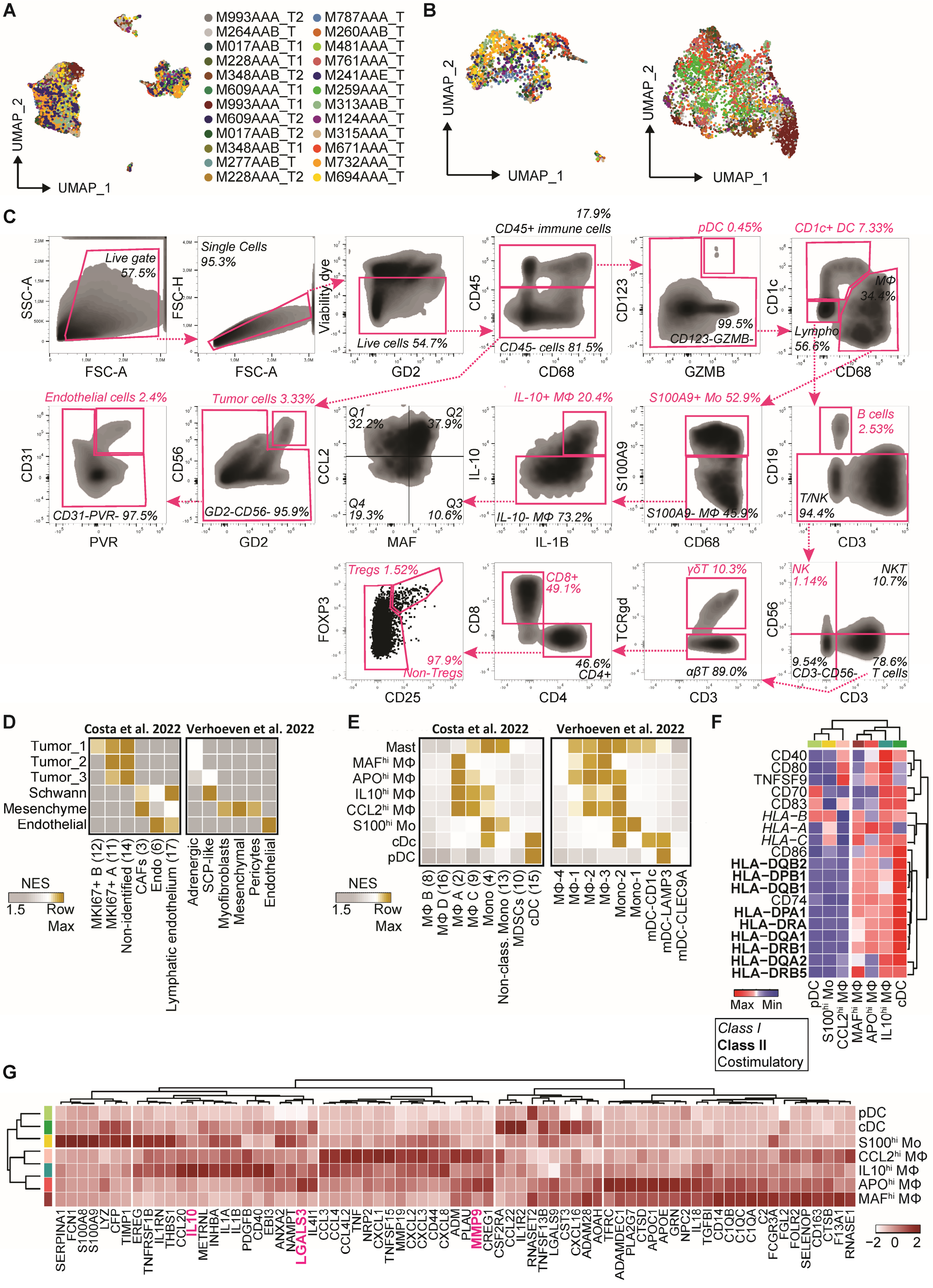
A
M993AAA_T2
M264AAB_T
M017AAB_T1
M228AAA_T1
M348AAB_T2
M609AAA_T1
M993AAA_T1
M609AAA_T2
M017AAB_T2
M348AAB_T1
M277AAB_T
M228AAA_T2
M787AAA_T
M260AAB_T
M481AAA_T
M761AAA_T
M241AAE_T
M259AAA_T
M313AAB_T
M124AAA_T
M315AAA_T
M671AAA_T
M732AAA_T
M694AAA_T
UMAP_1
UMAP_2
B
C
Live gate 57.5%
Single Cells 95.3%
Live cells 54.7%
17.9% CD45+ immune cells
CD45- cells 81.5%
pDC 0.45%
99.5% CD123-GZMB-
CD1c+ DC 7.33%
MΦ 34.4%
Lympho 56.6%
Endothelial cells 2.4%
CD31-PVR- 97.5%
Tumor cells 3.33%
GD2-CD56- 95.9%
Q1 32.2%
Q2 37.9%
Q4 19.3%
Q3 10.6%
IL-10+ MΦ 20.4%
IL-10- MΦ 73.2%
S100A9+ Mo 52.9%
S100A9- MΦ 45.9%
B cells 2.53%
T/NK 94.4%
Tregs 1.52%
97.9% Non-Tregs
CD8+ 49.1%
46.6% CD4+
γδT 10.3%
αβT 89.0%
NK 1.14%
NKT 10.7%
9.54% CD3-CD56-
78.6% T cells
D
Costa et al. 2022
Verhoeven et al. 2022
Tumor_1
Tumor_2
Tumor_3
Schwann
Mesenchyme
Endothelial
MKI67+ B (12)
MKI67+ A (11)
Non-identified (14)
CAFs (3)
Endo (6)
Lymphatic endothelium (17)
Adrenergic
SCP-like
Myofibroblasts
Mesenchymal
Pericytes
Endothelial
NES
1.5 Row Max
E
Mast
MAFhi MΦ
APOhi MΦ
IL10hi MΦ
CCL2hi MΦ
S100hi Mo
cDc
pDC
MΦ B (8)
MΦ D (16)
MΦ A (2)
MΦ C (9)
Mono (4)
Non-class. Mono (13)
MDSCs (10)
cDC (15)
MΦ-4
MΦ-1
MΦ-2
MΦ-3
Mono-2
Mono-1
mDC-CD1c
mDC-LAMP3
mDC-CLEC9A
F
CD40
CD80
TNFSF9
CD70
CD83
HLA-B
HLA-A
HLA-C
CD86
HLA-DQB2
HLA-DPB1
HLA-DQB1
CD74
HLA-DPA1
HLA-DRA
HLA-DQA1
HLA-DRB1
HLA-DQA2
HLA-DRB5
Max Min
pDC
S100hi Mo
CCL2hi MΦ
MAFhi MΦ
APOhi MΦ
IL10hi MΦ
cDC
Class I
Class II
Costimulatory
G
pDC
cDC
S100hi Mo
CCL2hi MΦ
IL10hi MΦ
APOhi MΦ
MAFhi MΦ
SERPINA1
FCN1
S100A8
S100A9
LYZ
CFP
TIMP1
EREG
TNFRSF1B
IL1RN
THBS1
CCL20
IL10
METRNL
INHBA
IL1A
IL1B
PDGFB
CD40
EBI3
ANXA2
NAMPT
LGALS3
IL4I1
CCL3
CCL4
CCL4L2
TNF
NRP2
CXCL1
TNFSF15
MMP19
CXCL2
CXCL3
CD44
CXCL8
ADM
PLAU
MMP9
CREG1
CSF2RA
CCL22
IL1R2
RNASET2
TNFSF13B
LGALS9
CST3
CXCL16
ADAM28
AOAH
TFRC
ADAMDEC1
PLA2G7
CTSD
APOC1
APOE
GRN
NPC2
IL18
TGFBI
CD14
C1QB
C1QC
C1QA
C2
FCGR3A
FGL2
FOLR2
SELENOP
CD163
CTSB
F13A1
RNASE1
-2 0 2

**Figure S2. The immune environment of neuroblastoma, related to figure 2. (A)** UMAP of sample distribution in immune compartment. **(B)** UMAP of sample distribution in myeloid cell (*left*) and lymphoid cell (*right*) compartments. **(C)** Flow cytometry gating strategy to identify cell populations in the tumor microenvironment. Merged data from 5 individual tumor samples are shown. **(D-E)** Gene set enrichment analysis comparing the identified non-immune **(D)** and myeloid **(E)** populations in our dataset to identified populations in the datasets of Costa et al. and Verhoeven et al.[4,5]**.** Gene signatures were derived from FindAllMarkers analysis in Seurat (genes with padj<0.05). NES=normalized enrichment score. **(F)** Heatmap of genes representing antigen presenting/co-stimulatory capacity. Genes are included in module score shown in Figure 2C. **(G)** Heatmap with secreted factors which were 1) specifically upregulated in myeloid cells compared to all other cells (padj<0.05) and 2) among the differentially expressed genes between the myeloid clusters (padj<0.05).

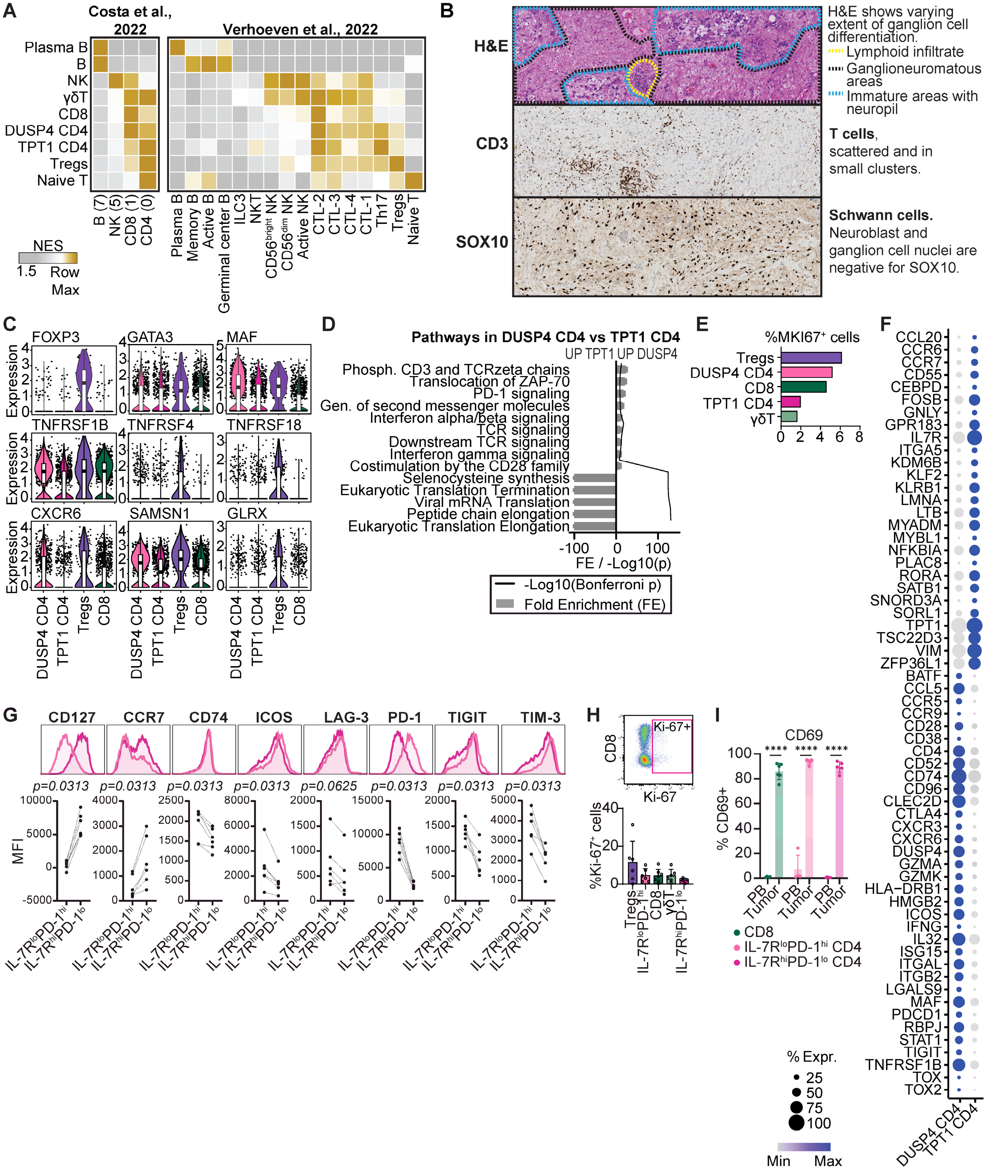

**Figure S3. Neuroblastoma is characterized by immunosuppressive and dysfunctional lymphoid populations, related to figure 3.** **(A)** Gene set enrichment analysis comparing the identified lymphoid populations in our dataset to identified lymphoid populations in the datasets of Costa et al. and Verhoeven et al.[4,5]**.** Gene signatures were derived from FindAllMarkers analysis in Seurat (genes with padj<0.05). NES=normalized enrichment score. **(B)** Immunohistochemical staining of hematoxylin & eosin (H&E), CD3 and SOX10 showing T cell infiltration in a representative neuroblastoma lesion. **(C)** Expression of effector Treg marker genes which are

significantly upregulated (*padj<0.05 in FindAllMarkers analysis among lymphocytes*) in neuroblastoma-infiltrating Tregs. **(D)** Pathway analysis (Reactome; Bonferroni-corrected p-value<0.05) of differentially expressed genes (padj<0.05) between the *DUSP4*$^{hi}$ and *TPT1*$^{hi}$ CD4$^{+}$ T cell populations. **(E)** Percentage of T cells expressing proliferation marker *MKI67*. **(F)** Dotplot with selected differentially expressed genes (*padj<0.05 in FindMarkers analysis*) between the two CD4$^{+}$ T effector populations. **(G)** Flow cytometric analysis of the two neuroblastoma-infiltrating CD4$^{+}$ T cell subsets showing memory, activation and exhaustion markers. *Mann-Whitney U test*; *MFI = median fluorescent intensity.* **(H)** Flow cytometric analysis of proliferation marker Ki-67 in neuroblastoma-infiltrating T cell subsets. **(I)** Flow cytometric analysis of tissue-resident T cell marker CD69 in neuroblastoma-infiltrating T cell subsets compared to their counterparts in healthy donor peripheral blood. TPT1$^{hi}$ CD4 were gated as IL-7R$^{hi}$PD-1$^{lo}$ CD4$^{+}$ cells and DUSP4$^{hi}$ CD4 were gated as IL-7R$^{lo}$PD-1$^{hi}$ CD4$^{+}$ cells, as shown in figure 3F. 2-way ANOVA. ****P<0.0001.

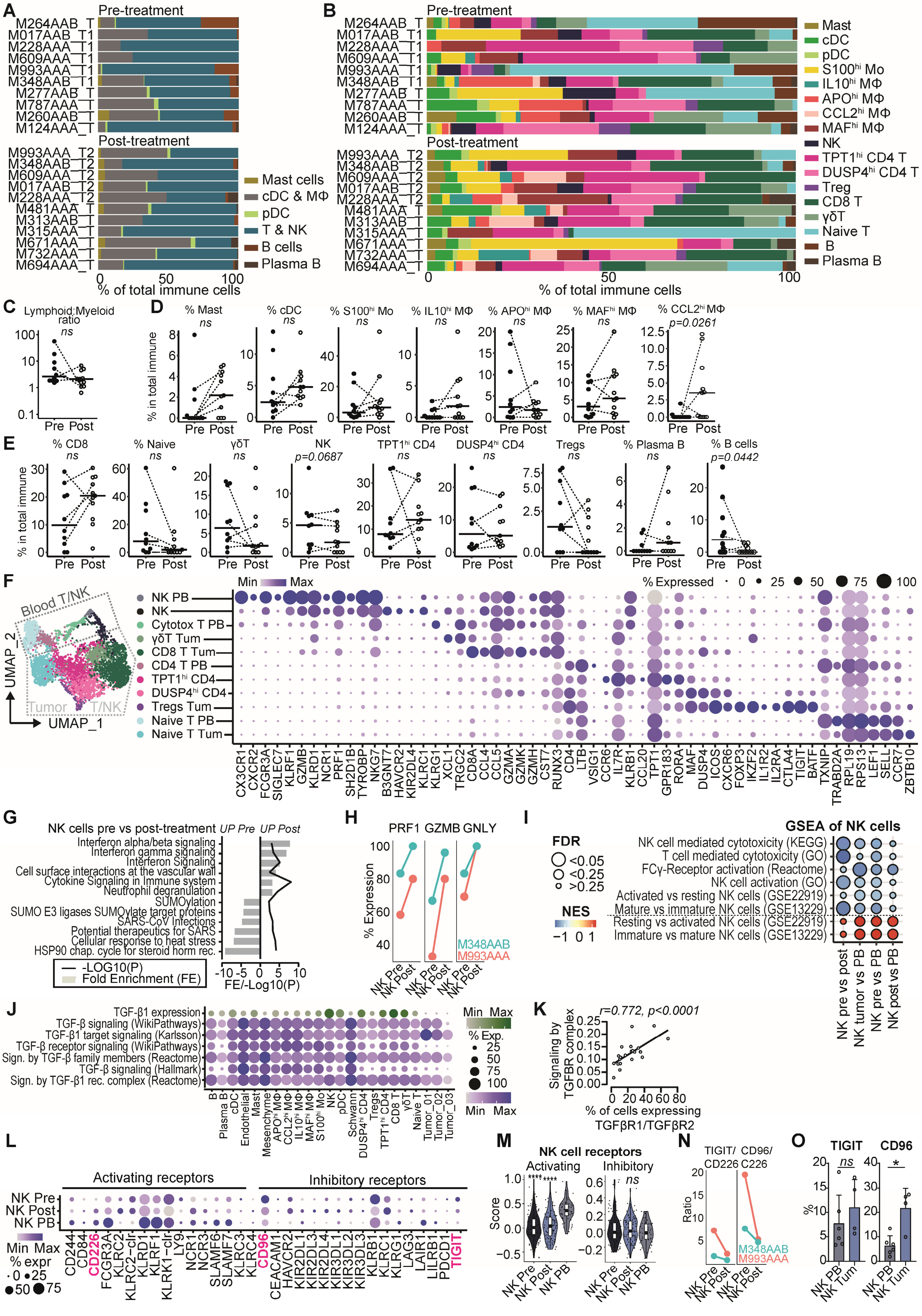

A
Pre-treatment
M264AAB_T
M017AAB_T1
M228AAA_T1
M609AAA_T1
M993AAA_T1
M348AAB_T1
M277AAB_T
M787AAA_T
M260AAB_T
M124AAA_T
Post-treatment
M993AAA_T2
M348AAB_T2
M609AAA_T2
M017AAB_T2
M228AAA_T2
M481AAA_T
M313AAB_T
M315AAA_T
M671AAA_T
M732AAA_T
M694AAA_T
Mast cells
cDC & MΦ
pDC
T & NK
B cells
Plasma B
% of total immune cells
B
Mast
cDC
pDC
S100hi Mo
IL10hi MΦ
APOhi MΦ
CCL2hi MΦ
MAFhi MΦ
NK
TPT1hi CD4 T
DUSP4hi CD4 T
Treg
CD8 T
γδT
Naive T
B
Plasma B
C
Lymphoid:Myeloid ratio
ns
Pre Post
D
% Mast
% cDC
% S100hi Mo
% IL10hi MΦ
% APOhi MΦ
% MAFhi MΦ
% CCL2hi MΦ
p=0.0261
% in total immune
E
% CD8
% Naive
γδT
NK
p=0.0687
TPT1hi CD4
DUSP4hi CD4
Tregs
% Plasma B
% B cells
p=0.0442
F
Blood T/NK
Tumor T/NK
UMAP_1
UMAP_2
NK PB
NK
Cytotox T PB
γδT Tum
CD8 T Tum
CD4 T PB
TPT1hi CD4
DUSP4hi CD4
Tregs Tum
Naive T PB
Naive T Tum
Min Max
% Expressed 0 25 50 75 100
CX3CR1 CXCR2 FCGR3A SIGLEC7 KLRF1 GZMB KLRD1 NCR1 PRF1 SH2D1B TYROBP NKG7 B3GNT7 HAVCR2 KIR2DL4 KLRC1 KLRG1 XCL1 TRGC2 CD8A CCL4 CCL5 GZMA GZMK GZMH CST7 RUNX3 CD4 LTB VSIG1 CCR6 IL7R KLRB1 CCL20 TPT1 GPR183 RORA MAF DUSP4 ICOS CXCR6 FOXP3 IKZF2 IL1R2 IL2RA CTLA4 TIGIT BATF TXNIP TRABD2A RPL19 RPS13 LEF1 SELL CCR7 ZBTB10
G
NK cells pre vs post-treatment
UP Pre UP Post
Interferon alpha/beta signaling
Interferon gamma signaling
Interferon Signaling
Cell surface interactions at the vascular wall
Cytokine Signaling in Immune system
Neutrophil degranulation
SUMOylation
SUMO E3 ligases SUMOylate target proteins
SARS-CoV Infections
Potential therapeutics for SARS
Cellular response to heat stress
HSP90 chap. cycle for steroid horm rec.
-LOG10(P)
Fold Enrichment (FE)
FE/-Log10(P)
H
PRF1 GZMB GNLY
% Expression
M348AAB
M993AAA
NK Pre
NK Post
I
GSEA of NK cells
FDR
<0.05
<0.25
>0.25
NES
-1 0 1
NK cell mediated cytotoxicity (KEGG)
T cell mediated cytotoxicity (GO)
FCγ-Receptor activation (Reactome)
NK cell activation (GO)
Activated vs resting NK cells (GSE22919)
Mature vs immature NK cells (GSE13229)
Resting vs activated NK cells (GSE22919)
Immature vs mature NK cells (GSE13229)
NK pre vs post
NK tumor vs PB
NK pre vs PB
NK post vs PB
J
TGF-β1 expression
TGF-β signaling (WikiPathways)
TGF-β1 target signaling (Karlsson)
TGF-β receptor signaling (WikiPathways)
Sign. by TGF-β family members (Reactome)
TGF-β signaling (Hallmark)
Sign. by TGF-β1 rec. complex (Reactome)
B Plasma B cDC Endothelial Mast Mesenchyme APOhi MΦ CCL2hi MΦ IL10hi MΦ MAFhi MΦ S100hi Mo NK pDC Schwann DUSP4hi CD4 Tregs TPT1hi CD4 CD8 T γδT Naive T Tumor_01 Tumor_02 Tumor_03
Min Max
% Exp. 25 50 75 100
K
r=0.772, p<0.0001
Signaling by TGFBR complex
% of cells expressing TGFβR1/TGFβR2
L
Activating receptors
Inhibitory receptors
NK Pre
NK Post
NK PB
Min Max
% expr 0 25 50 75
CD244 CD84 CD226 FCGR3A KLRC2 KLRC2-clr KLRD1 KLRF1 KLRK1-clr LY9 NCR1 NCR3 SLAMF6 SLAMF7 KLRC3 KLRC4 CD96 CEACAM1 HAVCR2 KIR2DL1 KIR2DL3 KIR2DL4 KIR3DL1 KIR3DL2 KIR3DL3 KLRB1 KLRC1 KLRG1 LAG3 LAIR1 LILRB1 PDCD1 TIGIT
M
NK cell receptors
Activating
Inhibitory
ns
Score
NK Pre NK Post NK PB
N
TIGIT/CD226
CD96/C226
Ratio
M348AAB
M993AAA
NK Pre NK Post
O
TIGIT
CD96
ns
*
%
NK PB NK Tum

**Figure S4. The immune cell composition and function in neuroblastoma before and after induction chemotherapy, related to figure 4. (A)** Composition of overall immune cells per patient before and after induction chemotherapy. "T1" and "T2" indicate paired samples before and after treatment. **(B)** Composition of detailed immune cell clusters per patient before and after induction chemotherapy. "T1" and "T2" indicate paired samples before and after treatment. **(C)** Lymphoid:myeloid ratio before and after chemotherapy. Dotted lines indicate paired samples. *Mann-Whitney U test.* **(D)** Myeloid cell percentages before and after induction chemotherapy. Dotted lines indicate paired samples. *Mann-Whitney U test.* **(E)** Lymphoid cell percentages before and after induction chemotherapy. Dotted lines indicate paired samples. *Mann-Whitney U test.* **(F)** UMAP and dotplot of integrated T/NK subsets from both tumor and reference peripheral blood (PB) of adult healthy donors showing a selection of their differentially expressed and marker genes. **(G)** Pathway analysis of NK cells with differentially expressed genes pre- versus post-treatment (nominal p<0.05; Reactome, Bonferroni-corrected p-values<0.05). **(H)** PRF1, GZMB and GNLY expression in two paired samples before and after treatment. The other 3 paired samples did not have sufficient NK cells for a reliable analysis. **(I)** Gene set enrichment analysis of NK cells in pre-treatment tumors compared to NK cells from post-treatment tumors and blood. *NK tumor = pre- and post-treatment NK cells combined. NES=normalized enrichment score*. **(J)** DotPlot of TGF-β1 expression and *modulescores* of curated signatures for TGF-β downstream signaling in the neuroblastoma TME[6]**. (K)** Pearson correlation between expression of signature for downstream signaling by the TGF-β receptor (TGFBR) complex (Reactome) in each population shown in figure S4J and the percentage of cells expressing a *modulescore* of TGF-β receptor 1/2 in those respective populations. **(L)** Dotplot of NK cell activating and inhibitory receptor expression. **(M)** Modulescore of NK cell activating and inhibitory receptors shown in Figure S4L. *Kruskall-Wallis with Dunn's, ****P<0.0001 vs NK PB, ns=not significant*. **(N)** The TIGIT/CD226 and CD96/CD226 gene expression ratio in two paired samples before and after treatment. The other 3 paired samples did not have sufficient NK cells for a reliable analysis. **(O)** Flow cytometric analysis of TIGIT and CD96 expression in neuroblastoma-infiltrating NK cells compared to blood NK cells. *Mann-Whitney U test; ns=not significant.*

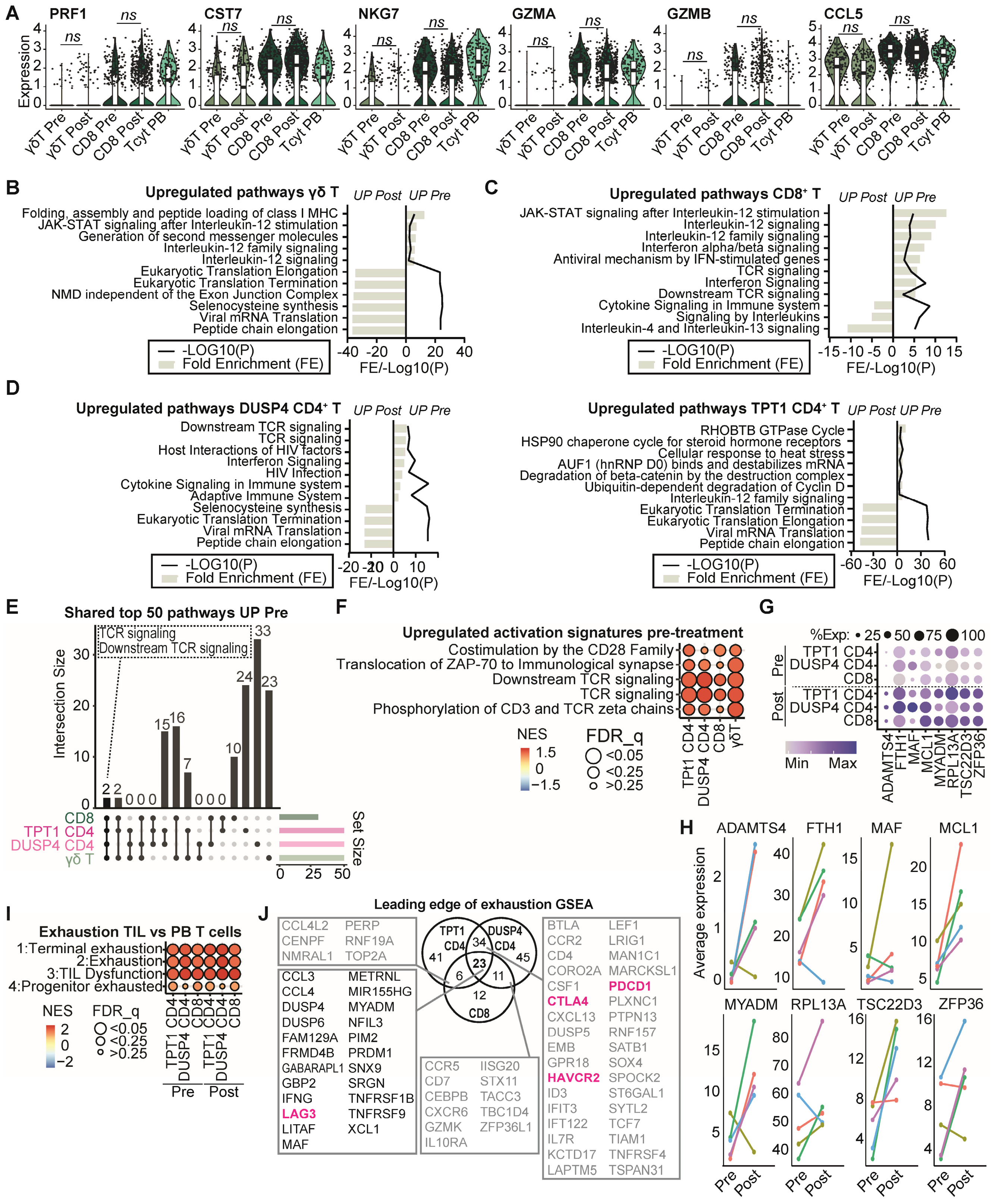


**Figure S5. T cells in neuroblastoma show increased dysfunctionality post-treatment, related to figure 4. (A)** Violin plots of genes associated with cytotoxicity in γδ T and CD8 T cells pre- and post-treatment compared to cytotoxic T cells in reference blood (Tcyt PB). *ns=not significant.* **(B-D)** Pathway analysis of differentially expressed genes (padj<0.05) in γδ T **(B)**, CD8 T **(C)** and CD4 T **(D)** pre-versus post-treatment (Reactome, Bonferroni-corrected p-values<0.05). **(E)** UpsetR 'Venn' diagram of top 50 upregulated pathways pre-treatment compared to post-treatment in the T cell subsets. **(F)** Gene set enrichment analysis (GSEA) with gene signatures for

TCR signaling in T cells pre-treatment vs post-treatment (all Reactome signatures). **(G)** Dotplot of shared upregulated genes comparing expression in CD4 and CD8 T cells pre- and post-treatment. **(H)** Analysis of gene expression of the eight shared upregulated genes post-treatment in αβ T cells in five paired samples pre-treatment and post-treatment. **(I)** GSEA of exhaustion signatures (terminal & progenitor: GSE84105, exhaustion: [7], dysfunction: [8]) in indicated CD4 and CD8 T cell clusters versus their respective counterparts from reference blood. **(J)** Venn diagram of shared upregulated genes post-treatment versus pre-treatment, which are present in leading edges (core enriched) of GSEA analysis with exhaustion/dysfunction signatures from Figure 4N.

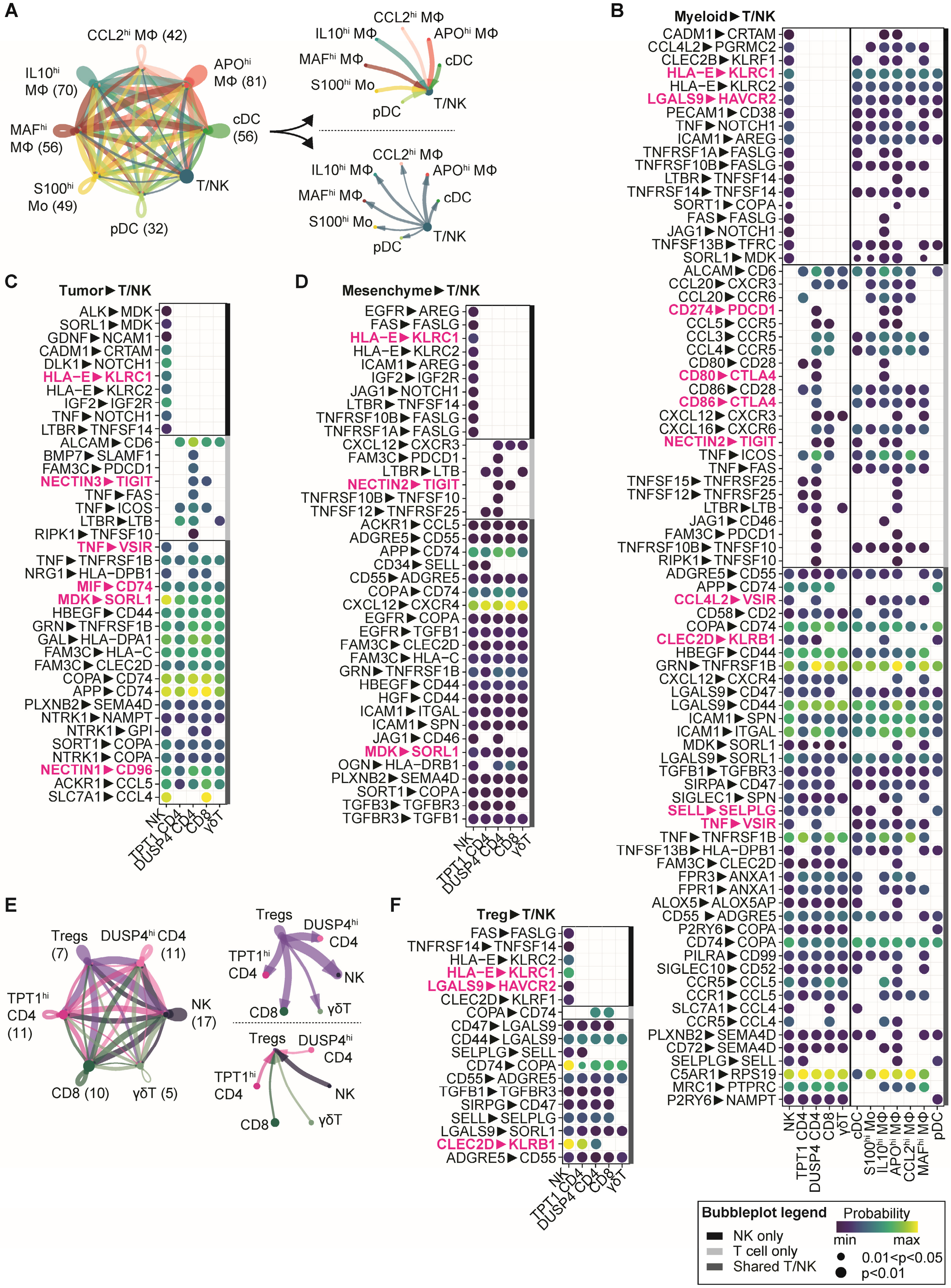

A
CCL2hi MΦ (42)
APOhi MΦ (81)
IL10hi MΦ (70)
MAFhi MΦ (56)
cDC (56)
S100hi Mo (49)
pDC (32)
T/NK
CCL2hi MΦ
IL10hi MΦ
APOhi MΦ
MAFhi MΦ
cDC
S100hi Mo
pDC
T/NK
B
Myeloid►T/NK
CADM1►CRTAM
CCL4L2►PGRMC2
CLEC2B►KLRF1
HLA−E►KLRC1
HLA−E►KLRC2
LGALS9►HAVCR2
PECAM1►CD38
TNF►NOTCH1
ICAM1►AREG
TNFRSF1A►FASLG
TNFRSF10B►FASLG
LTBR►TNFSF14
TNFRSF14►TNFSF14
SORT1►COPA
FAS►FASLG
JAG1►NOTCH1
TNFSF13B►TFRC
SORL1►MDK
ALCAM►CD6
CCL20►CXCR3
CCL20►CCR6
CD274►PDCD1
CCL5►CCR5
CCL3►CCR5
CCL4►CCR5
CD80►CD28
CD80►CTLA4
CD86►CD28
CD86►CTLA4
CXCL12►CXCR3
CXCL16►CXCR6
NECTIN2►TIGIT
TNF►ICOS
TNF►FAS
TNFSF15►TNFRSF25
TNFSF12►TNFRSF25
LTBR►LTB
JAG1►CD46
FAM3C►PDCD1
TNFRSF10B►TNFSF10
RIPK1►TNFSF10
ADGRE5►CD55
APP►CD74
CCL4L2►VSIR
CD58►CD2
COPA►CD74
CLEC2D►KLRB1
HBEGF►CD44
GRN►TNFRSF1B
CXCL12►CXCR4
LGALS9►CD47
LGALS9►CD44
ICAM1►SPN
ICAM1►ITGAL
MDK►SORL1
LGALS9►SORL1
TGFB1►TGFBR3
SIRPA►CD47
SIGLEC1►SPN
SELL►SELPLG
TNF►VSIR
TNF►TNFRSF1B
TNFSF13B►HLA−DPB1
FAM3C►CLEC2D
FPR3►ANXA1
FPR1►ANXA1
ALOX5►ALOX5AP
CD55►ADGRE5
P2RY6►COPA
CD74►COPA
PILRA►CD99
SIGLEC10►CD52
CCR5►CCL5
CCR1►CCL5
SLC7A1►CCL4
CCR5►CCL4
PLXNB2►SEMA4D
CD72►SEMA4D
SELPLG►SELL
C5AR1►RPS19
MRC1►PTPRC
P2RY6►NAMPT
NK
TPT1 CD4
DUSP4 CD4
CD8
γδT
cDC
S100hi Mo
IL10hi MΦ
APOhi MΦ
CCL2hi MΦ
MAFhi MΦ
pDC
C
Tumor►T/NK
ALK►MDK
SORL1►MDK
GDNF►NCAM1
CADM1►CRTAM
DLK1►NOTCH1
HLA−E►KLRC1
HLA−E►KLRC2
IGF2►IGF2R
TNF►NOTCH1
LTBR►TNFSF14
ALCAM►CD6
BMP7►SLAMF1
FAM3C►PDCD1
NECTIN3►TIGIT
TNF►FAS
TNF►ICOS
LTBR►LTB
RIPK1►TNFSF10
TNF►VSIR
TNF►TNFRSF1B
NRG1►HLA−DPB1
MIF►CD74
MDK►SORL1
HBEGF►CD44
GRN►TNFRSF1B
GAL►HLA−DPA1
FAM3C►HLA−C
FAM3C►CLEC2D
COPA►CD74
APP►CD74
PLXNB2►SEMA4D
NTRK1►NAMPT
NTRK1►GPI
SORT1►COPA
NTRK1►COPA
NECTIN1►CD96
ACKR1►CCL5
SLC7A1►CCL4
NK
TPT1 CD4
DUSP4 CD4
CD8
γδT
D
Mesenchyme►T/NK
EGFR►AREG
FAS►FASLG
HLA−E►KLRC1
HLA−E►KLRC2
ICAM1►AREG
IGF2►IGF2R
JAG1►NOTCH1
LTBR►TNFSF14
TNFRSF10B►FASLG
TNFRSF1A►FASLG
CXCL12►CXCR3
FAM3C►PDCD1
LTBR►LTB
NECTIN2►TIGIT
TNFRSF10B►TNFSF10
TNFSF12►TNFRSF25
ACKR1►CCL5
ADGRE5►CD55
APP►CD74
CD34►SELL
CD55►ADGRE5
COPA►CD74
CXCL12►CXCR4
EGFR►COPA
EGFR►TGFB1
FAM3C►CLEC2D
FAM3C►HLA−C
GRN►TNFRSF1B
HBEGF►CD44
HGF►CD44
ICAM1►ITGAL
ICAM1►SPN
JAG1►CD46
MDK►SORL1
OGN►HLA−DRB1
PLXNB2►SEMA4D
SORT1►COPA
TGFB3►TGFBR3
TGFBR3►TGFB1
NK
TPT1 CD4
DUSP4 CD4
CD8
γδT
E
Tregs (7)
DUSP4hi CD4 (11)
TPT1hi CD4 (11)
NK (17)
CD8 (10)
γδT (5)
Tregs
DUSP4hi CD4
TPT1hi CD4
NK
CD8
γδT
Tregs
DUSP4hi CD4
TPT1hi CD4
NK
CD8
γδT
F
Treg►T/NK
FAS►FASLG
TNFRSF14►TNFSF14
HLA−E►KLRC2
HLA−E►KLRC1
LGALS9►HAVCR2
CLEC2D►KLRF1
COPA►CD74
CD47►LGALS9
CD44►LGALS9
SELPLG►SELL
CD74►COPA
CD55►ADGRE5
TGFB1►TGFBR3
SIRPG►CD47
SELL►SELPLG
LGALS9►SORL1
CLEC2D►KLRB1
ADGRE5►CD55
NK
TPT1 CD4
DUSP4 CD4
CD8
γδT
Bubbleplot legend
Probability
min
max
NK only
T cell only
Shared T/NK
0.01<p<0.05
p<0.01

**Figure S6. Immunoregulatory interactions in the neuroblastoma tumor-microenvironment, related to figure 5. (A)** Interaction network involving interactions between myeloid cells and T/NK cells constructed with CellChat[9]. **(B)** Bubbleplot of predicted interactions between myeloid cells and indicated T/NK subsets. Interactions with each specific T/NK subset and each specific myeloid subset were evaluated and subsequently merged, with the highest probability of each interaction pair depicted in the plot (*left: myeloid merged; right: T/NK merged*) **(C)** Bubbleplot of predicted interactions between tumor cells and indicated T/NK subsets. **(D)** Bubbleplot of predicted interactions between mesenchymal cells and indicated T/NK subsets. **(E)** Interaction network of interactions among Tregs and all other T/NK cell subsets. **(F)** Bubbleplot involving interactions between Tregs and indicated T/NK cell subsets.

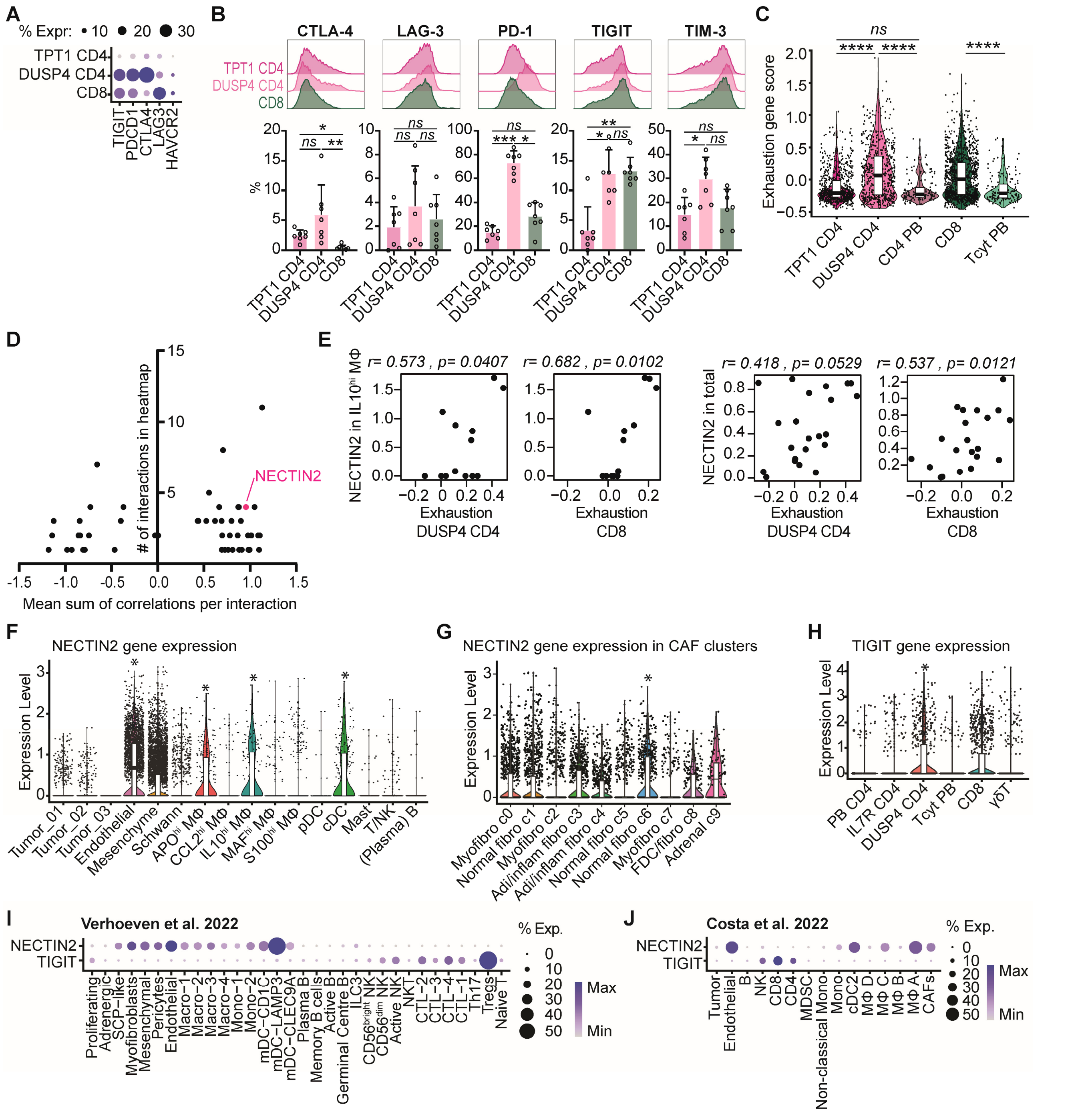


**Figure S7. The *NECTIN2-TIGIT* axis is associated with T cell dysfunction, related to figure 6. (A)** Dotplot with expression of dysfunction/exhaustion markers *CTLA4*, *LAG3*, *TIGIT*, *PDCD1* and *HAVCR2* in the indicated T cell subsets. **(B)** Flow cytometric analysis of CTLA-4, LAG-3, TIGIT, PD-1 and TIM-3 expression on neuroblastoma-infiltrating T cell subsets. TPT1$^{hi}$ CD4 were gated as IL-7R$^{hi}$PD-1$^{lo}$ CD4$^{+}$ cells and DUSP4$^{hi}$ CD4 were gated as IL-7R$^{lo}$PD-1$^{hi}$ CD4$^{+}$ cells, as shown in figure 3F. *Kruskall Wallis + Dunn's. *P<0.05, **P<0.01, ***P<0.001, ns=not significant.* **(C)** Modulescore of the dysfunction/exhaustion genes in the indicated T cell subsets. *Kruskall Wallis + Dunn's. ****P<0.0001*. **(D)** NECTIN2 'ranking' among genes "B" from figure 6B which correlate positively with dysfunction score of T cells, showing the mean sum of correlations of all interactions involving gene "B" in figure 6B, and the total number of interactions involving gene "B" in figure 6B. **(E)** Correlation between *NECTIN2* expression in IL10hi Mϕ and total cells in the TME with exhaustion scores of *DUSP4*$^{hi}$ CD4 and CD8 T cells. **(F)** NECTIN2 gene expression in neuroblastoma TME. **P<0.0001* from *Findallmarkers* analysis. **(G)** NECTIN2 gene expression in detailed mesenchyme clusters. **P<0.0001* from *Findallmarkers* analysis. **(H)** TIGIT gene expressing on T cells infiltrating neuroblastoma compared to T cells from healthy donor blood. **P<0.0001* from *Findallmarkers* analysis. **(I)** TIGIT and NECTIN2 expression in dataset of Verhoeven et al.[5] **(J)** TIGIT and NECTIN2 expression in dataset of Costa et al.[4]

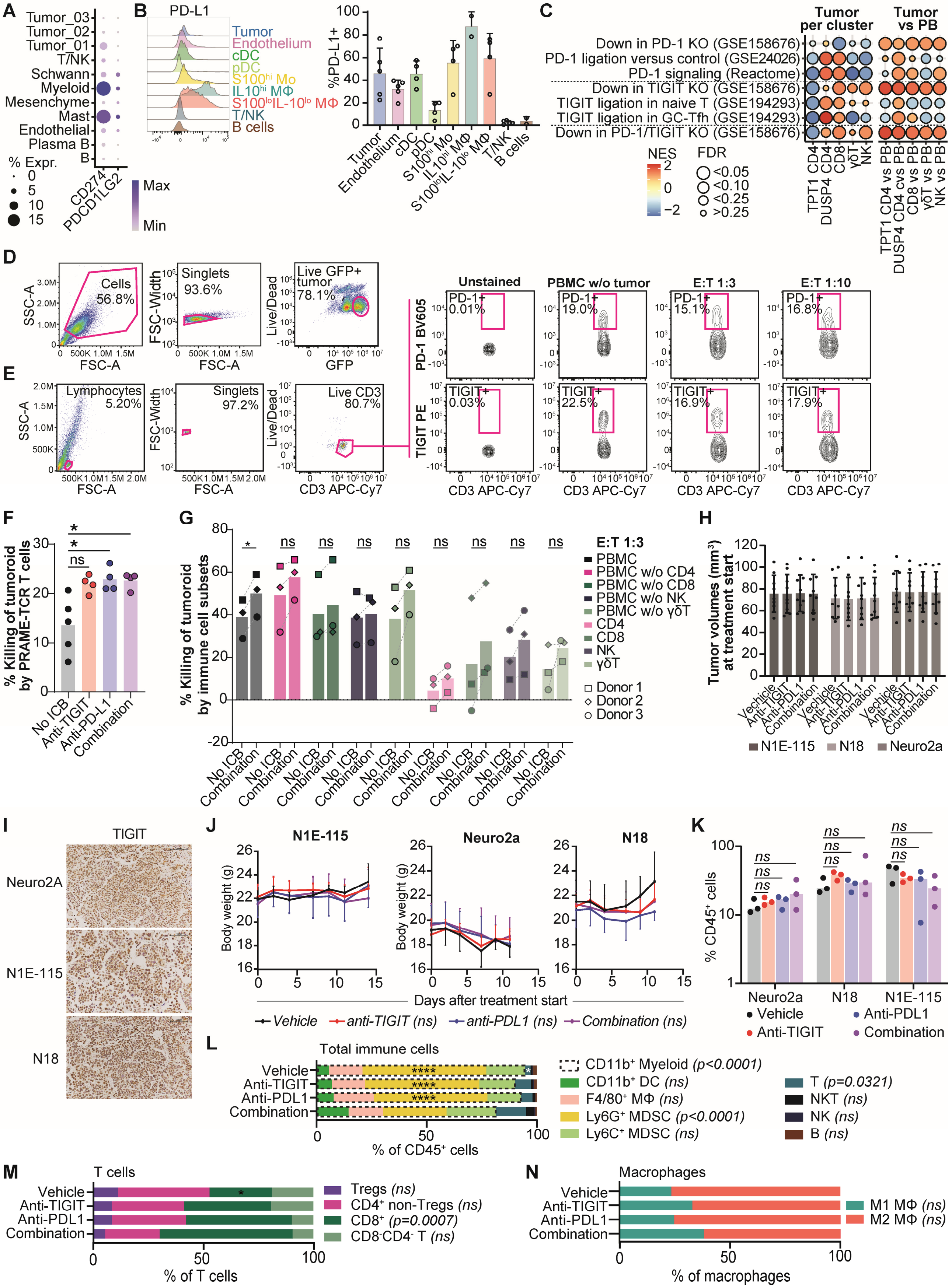

A
Tumor_03
Tumor_02
Tumor_01
T/NK
Schwann
Myeloid
Mesenchyme
Mast
Endothelial
Plasma B
B
% Expr.
0
5
10
15
CD274
PDCD1LG2
Max
Min
B
PD-L1
Tumor
Endothelium
cDC
pDC
S100hi Mo
IL10hi MΦ
S100loIL-10lo MΦ
T/NK
B cells
%PD-L1+
C
Tumor per cluster
Tumor vs PB
Down in PD-1 KO (GSE158676)
PD-1 ligation versus control (GSE24026)
PD-1 signaling (Reactome)
Down in TIGIT KO (GSE158676)
TIGIT ligation in naive T (GSE194293)
TIGIT ligation in GC-Tfh (GSE194293)
Down in PD-1/TIGIT KO (GSE158676)
NES
FDR
<0.05
<0.10
<0.25
>0.25
TPT1 CD4
DUSP4 CD4
CD8
γδT
NK
TPT1 CD4 vs PB
DUSP4 CD4 cvs PB
CD8 vs PB
γδT vs PB
NK vs PB
D
Cells 56.8%
Singlets 93.6%
Live GFP+ tumor 78.1%
SSC-A
FSC-A
FSC-Width
Live/Dead
GFP
Unstained
PBMC w/o tumor
E:T 1:3
E:T 1:10
PD-1+ 0.01%
PD-1+ 19.0%
PD-1+ 15.1%
PD-1+ 16.8%
PD-1 BV605
E
Lymphocytes 5.20%
Singlets 97.2%
Live CD3 80.7%
TIGIT+ 0.03%
TIGIT+ 22.5%
TIGIT+ 16.9%
TIGIT+ 17.9%
TIGIT PE
CD3 APC-Cy7
F
% Killing of tumoroid by PRAME-TCR T cells
No ICB
Anti-TIGIT
Anti-PD-L1
Combination
ns
G
% Killing of tumoroid by immune cell subsets
E:T 1:3
PBMC
PBMC w/o CD4
PBMC w/o CD8
PBMC w/o NK
PBMC w/o γδT
CD4
CD8
NK
γδT
Donor 1
Donor 2
Donor 3
No ICB
Combination
H
Tumor volumes (mm³) at treatment start
Vechicle
Anti-TIGIT
Anti-PDL1
Combination
N1E-115
N18
Neuro2a
I
TIGIT
Neuro2A
N1E-115
N18
J
N1E-115
Neuro2a
N18
Body weight (g)
Days after treatment start
Vehicle
anti-TIGIT (ns)
anti-PDL1 (ns)
Combination (ns)
K
% CD45+ cells
Neuro2a
N18
N1E-115
Vehicle
Anti-TIGIT
Anti-PDL1
Combination
L
Total immune cells
Vehicle
Anti-TIGIT
Anti-PDL1
Combination
% of CD45+ cells
CD11b+ Myeloid (p<0.0001)
CD11b+ DC (ns)
F4/80+ MΦ (ns)
Ly6G+ MDSC (p<0.0001)
Ly6C+ MDSC (ns)
T (p=0.0321)
NKT (ns)
NK (ns)
B (ns)
M
T cells
Tregs (ns)
CD4+ non-Tregs (ns)
CD8+ (p=0.0007)
CD8-CD4- T (ns)
% of T cells
N
Macrophages
M1 MΦ (ns)
M2 MΦ (ns)
% of macrophages

**Figure S8. Combined TIGIT/PD-L1 blockade enhances immune responses against neuroblastoma *in vitro* and *in vivo*, related to figure 7. (A)** DotPlot with expression of PD-1 ligands *CD274* (PD-L1) and *PDCD1LG2* (PD-L2) in neuroblastoma. **(B)** Flow cytometric analysis of PD-L1 expression in the neuroblastoma tumor-microenvironment. **(C)** Gene set enrichment analysis of selected signatures for PD-1 and/or TIGIT downstream signaling. KO=knock-out, GC-Tfh=germinal center follicular helper T cells. NES=normalized enrichment score. **(D)** Gating strategy for flow cytometric expression analysis of Nectin-2 and PD-L1 expression on GFP-positive tumoroids as shown in Figure 7C. **(E)** Flow cytometric analysis of PD-1 and TIGIT expression on healthy donor PBMC cocultured with neuroblastoma tumoroids for 6 days. 'PBMC w/o tumor' was measured at timepoint 0 before coculture. **(F)** Percentage of tumoroid (AMC691T) killing (=100-normalized luminescence) *in vitro* after 6 days of co-culture with PRAME-TCR transduced T cells at E:T ratio 1:300. Luminescence values were normalized against condition with untreated tumoroids only. n=4-5 technical replicates. *Kruskall-Wallis with Dunn's.*$p<0.05$; ns=not significant.* **(G)** Percentage of tumoroid (AMC691T) killing (=100-normalized luminescence) *in vitro* after 6 days of co-culture with FACS-sorted immune subsets from healthy donor peripheral blood at E:T ratio 1:3. Luminescence values were normalized against condition with untreated tumoroids only. n=3 donors. *Two-way ANOVA with Sidak*. **$p<0.05$; ns=not significant. w/o=without.* **(H)** Tumor volumes at treatment start per condition for the three *in vivo* models. **(I)** Immunohistochemical staining of TIGIT, PD-1 and PD-L1 in the three *in vivo* models (vehicle condition). **(J)** Body weight of N1E-115, Neuro2a and N18 mouse models (n=6 per group) treated with anti-TIGIT and/or anti-PD-L1 up to day 15 of treatment. **(K-N)** Flow cytometric analysis of the TME *in vivo* in n=3 mice per treatment condition, treated with anti-TIGIT and/or anti-PD-L1 for 7 days. **(K)** Percentage of CD45+ immune cells of total live cells. **(L)** Total immune cell composition across treatments. Models were pooled. **$P<0.05$ versus combination, ****$P<0.0001$ versus combination. 2-Way ANOVA.* **(M)** T cell composition across treatments. Models were pooled. **$P<0.05$ versus combination. 2-Way ANOVA.* **(N)** Macrophage composition across treatments. Models were pooled. *2-Way ANOVA.*

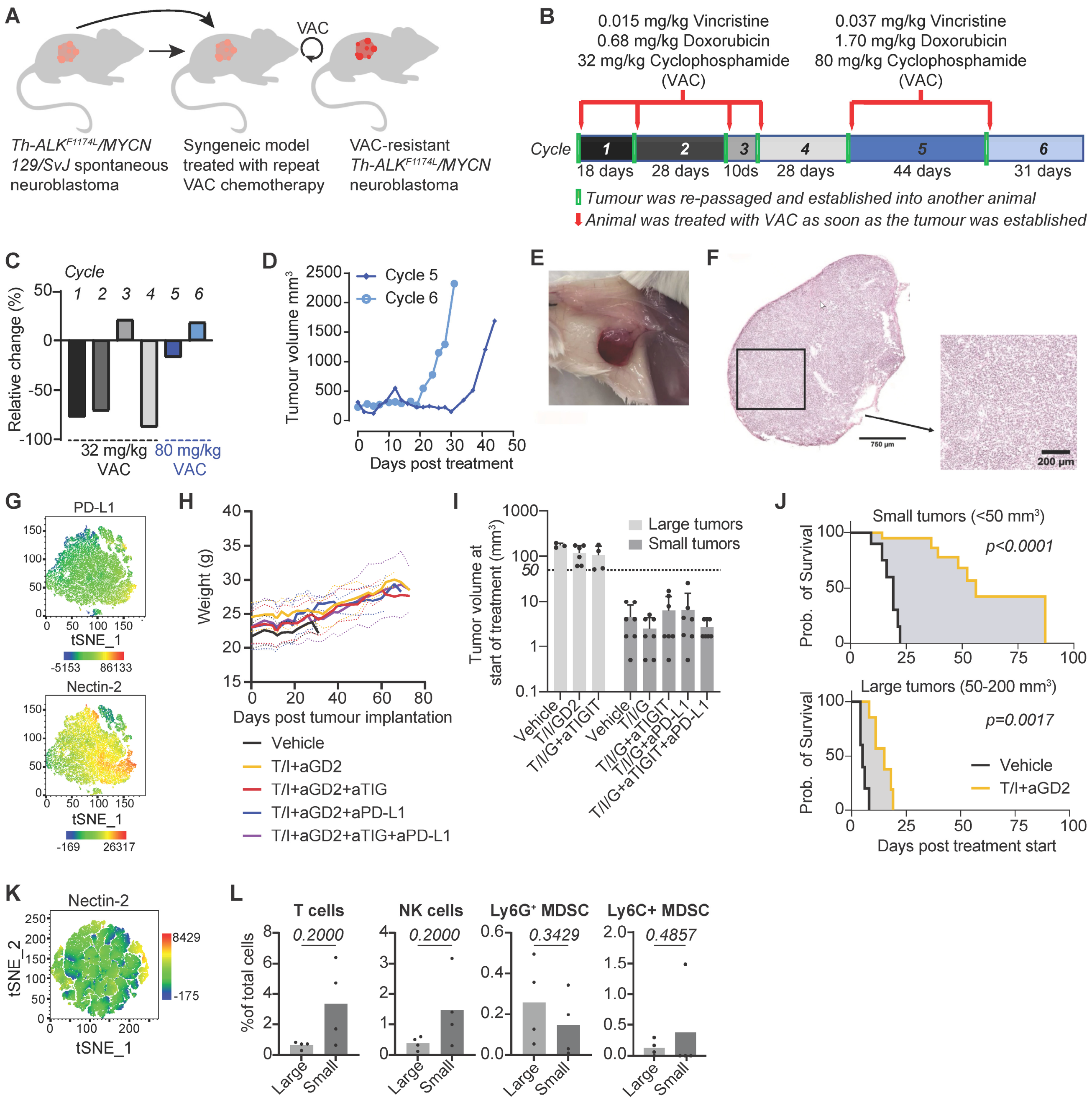

**Figure S9. TIGIT blockade improves survival in a chemotherapy-resistant neuroblastoma model, related to figure 8. (A)** Generation of chemotherapy-resistant neuroblastoma model from *Th-ALK*$^{F1174L}$/*MYCN* 129/SvJ models repeatedly treated with VAC (Vincristine, Adriamycin/Doxorubicin, Cyclophosphamide). **(B)** Summary of dose escalation treatment schedule to achieve resistance. Red arrows indicate when single dose VAC was administered. The number of days taken for the tumor to relapse is indicated below each cycle. At the end of each cycle, the allograft tumor was re-passaged into another animal before treatment started. **(C)** Percentage relative tumor volume change 7 days after treatment with single dose of VAC at 32 mg/kg or 80 mg/kg. **(D)** Time taken for allograft tumor to relapse after treatment with single high dose of VAC (80 mg/kg VAC) in Cycle 5 and Cycle 6. Tumor cells after Cycle 6 were used to establish allografts for therapeutic studies. **(E)** Macroscopic confirmation of tumor presence for small tumors. **(F)** Histological confirmation of tumor cell presence for small tumors. **(G)** TSNE representation of flow cytometric analysis of PD-L1 and Nectin-2 expression in the TME of small tumors (vehicle condition). **(H)** Body weight of mice with small tumors (n=7 per group) treated with TEM/IRI, anti-GD2 and/or anti-TIGIT and/or anti-PD-L1 up to day 80 of treatment. **(I)** Tumor volumes at start of treatment per treatment condition for mice with large and small tumors. **(J)** Survival analysis comparing response to TEM/IRI (T/I) +anti-GD2 (aGD2) in small and large tumors. **(K)** TSNE representation of flow cytometric analysis of Nectin-2 expression in the TME of large tumors (vehicle condition). **(L)** Flow cytometric analysis of immune cell subsets as a fraction of total live cells in the tumor. *Mann Whitney U test. N=4 animals per condition.*

**References**


1. Luo, H., Xia, X., Huang, L.-B., An, H., Cao, M., Kim, G.D., Chen, H.-N., Zhang, W.-H., Shu, Y., Kong, X., et al. (2022). Pan-cancer single-cell analysis reveals the heterogeneity and plasticity of cancer-associated fibroblasts in the tumor microenvironment. Nat. Commun. *13*, 6619. 10.1038/s41467-022-34395-2.
2. Bedoya-Reina, O.C., Li, W., Arceo, M., Plescher, M., Bullova, P., Pui, H., Kaucka, M., Kharchenko, P., Martinsson, T., Holmberg, J., et al. (2021). Single-nuclei transcriptomes from human adrenal gland reveal distinct cellular identities of low and high-risk neuroblastoma tumors. Nat. Commun. *12*, 5309. 10.1038/s41467-021-24870-7.
3. Abe, Y., Sakata-Yanagimoto, M., Fujisawa, M., Miyoshi, H., Suehara, Y., Hattori, K., Kusakabe, M., Sakamoto, T., Nishikii, H., Nguyen, T.B., et al. (2022). A single-cell atlas of non-haematopoietic cells in human lymph nodes and lymphoma reveals a landscape of stromal remodelling. Nat. Cell Biol. *24*, 565–578. 10.1038/s41556-022-00866-3.
4. Costa, A., Thirant, C., Kramdi, A., Pierre-Eugène, C., Louis-Brennetot, C., Blanchard, O., Surdez, D., Gruel, N., Lapouble, E., Pierron, G., et al. (2022). Single-cell transcriptomics reveals shared immunosuppressive landscapes of mouse and human neuroblastoma. J. Immunother. cancer *10*. 10.1136/jitc-2022-004807.
5. Verhoeven, B.M., Mei, S., Olsen, T.K., Gustafsson, K., Valind, A., Lindström, A., Gisselsson, D., Fard, S.S., Hagerling, C., Kharchenko, P. V, et al. (2022). The immune cell atlas of human neuroblastoma. Cell reports. Med. *3*, 100657. 10.1016/j.xcrm.2022.100657.
6. Karlsson, G., Liu, Y., Larsson, J., Goumans, M.-J., Lee, J.-S., Thorgeirsson, S.S., Ringnér, M., and Karlsson, S. (2005). Gene expression profiling demonstrates that TGF-beta1 signals exclusively through receptor complexes involving Alk5 and identifies targets of TGF-beta signaling. Physiol. Genomics *21*, 396–403. 10.1152/physiolgenomics.00303.2004.
7. Man, K., Gabriel, S.S., Liao, Y., Gloury, R., Preston, S., Henstridge, D.C., Pellegrini, M., Zehn, D., Berberich-Siebelt, F., Febbraio, M.A., et al. (2017). Transcription Factor IRF4 Promotes CD8(+) T Cell Exhaustion and Limits the Development of Memory-like T Cells during Chronic Infection. Immunity *47*, 1129-1141.e5. 10.1016/j.immuni.2017.11.021.
8. Li, H., van der Leun, A.M., Yofe, I., Lubling, Y., Gelbard-Solodkin, D., van Akkooi, A.C.J., van den Braber, M., Rozeman, E.A., Haanen, J.B.A.G., Blank, C.U., et al. (2019). Dysfunctional CD8 T Cells Form a Proliferative, Dynamically Regulated Compartment within Human Melanoma. Cell *176*, 775-789.e18. 10.1016/j.cell.2018.11.043.
9. Jin, S., Guerrero-Juarez, C.F., Zhang, L., Chang, I., Ramos, R., Kuan, C.-H., Myung, P., Plikus, M. V, and Nie, Q. (2021). Inference and analysis of cell-cell communication using CellChat. Nat. Commun. *12*, 1088. 10.1038/s41467-021-21246-9.
